# Supplementary material for: ‘They’ve all endorsed it…but I’m just not there:’ a qualitative exploration of COVID-19 vaccine hesitancy reported by Black and Latinx individuals
Source: BMJ Open. 2023 Jul 20;13(7):e072619. doi: 10.1136/bmjopen-2023-072619 (PMC10360437; doi:10.1136/bmjopen-2023-072619)
Supplement: Supplementary data [file bmjopen-2023-072619supp001.pdf]

Critica Bulletin Board Discussion Guide:
Exploring Hesitancy to Vaccinate Among Latinx and African-American Communities
May 27, 2021

All questions will be partially masked (respondents can only see others' responses once they have submitted their own response). All questions will be text response (open-ended) questions unless otherwise indicated.

Introduction and Instructions:

[Multiple choice question] Welcome! My name is **Marsha**, and I will be guiding you through our online discussion over the next three days. Before we get started, please read the following information carefully and consent to participate in the discussion.

> **[INSERT CONSENT TEXT]**
>
> ( ) Yes
> ( ) No [Terminate]

[Notice] Here's how the discussion will work…

> Over the next three days, I will ask you questions related to the topic of vaccines. My job is to get your thoughts and opinions about this topic, and your job is to share them by typing your responses, and then submitting them. I am interested in your **honest** opinions, be they positive or negative, so please share freely and honestly throughout the discussion. There are no "right" or "wrong" answers, and you don't have to worry about your spelling or sentence structure. If you share your honest opinions and attitudes, then you will be providing the "right" answers.

Once you submit your response to a question, you will be able to see how the rest of the group has responded and you can "like" or comment on their posts if you have anything to add. We are all different individuals in this discussion, so I expect there will be a variety of different experiences and opinions. That's great! If it looks to you like you have a different opinion than most others, please be sure to share it! It is important that you all be honest, and respectful of others who might think differently than you do.

I'm hoping to learn as much from you as possible, and encourage you not only tell me **what** you think, but **WHY**. The more details you share to explain your opinions, the better! This is not like Twitter or other social media where there's a limit to how much you can write. In fact, it's just the opposite! Tell me as much as you want in response to each question, especially WHY your thoughts and opinions are what they are. Please share examples or experiences that you have had that help to explain your response. For example, "Just last week, I was going to the grocery store, and…." Or "My niece told me that one of her teachers said...." Stories or examples like this really help me to understand what shapes your opinions.

I will be posting new questions each day – once in the morning, and once in the early afternoon, so it is important that you visit the website at least twice every day. It will probably take you about 30 minutes per day to answer the questions. You can spend as much time on the site as you desire.

To answer a question or respond to a post, simply click on the button in the bottom left corner that says "You have not replied. Click here," type your response in the space provided, and click on the "Submit" button. Please completely answer each question fully and be as conversational as possible, details are great! Any questions that you have not answered will have a button stating "You have not replied. Click here". This way you can easily tell what you still need to answer.

Keep an eye out for follow-up questions from me. I may ask questions of you specifically or to the group as a whole. To easily see if you have follow-ups you can

look at your Project Alerts box on the left of your screen and if you see a number beside "Unanswered Follow Up Questions" you'll know I have put something in specifically for you. Just click on that number and it will be brought up on the screen for you.

Here are some more tips for you to make this process easy and enjoyable:

- Please watch the "Participant Intro Video" found on the "Dashboard (Home)" page of this site. There is a lot of really great information in there to help you out along the way!
- Please upload a photo or an avatar of yourself so that I can associate a face with your name. It just makes our discussion a little more personable. You can do this under "My Profile" in the upper left-hand corner of the site.
- You can click "Highlight Unanswered Questions" on the top of the Navigation Section to see if there are any questions from me that you may have missed. To make things even easier…on the left-hand side of the screen you'll see the number of questions or follow ups that I may have left for you! Just look for the blue box titled "Project Alerts."
- On the left of your screen you'll see a tab titled "Message Center". Click on that feature to see any emails that I might have sent to you that you might have missed in your personal email inbox.
- There are some really good help articles and "How-To's" HERE if you run into questions about how to use QualBoard (you can also click "Help Desk" on the top right of your screen to get to this site).

If you have technical difficulties of any kind while participating in this discussion, please click "Chat with QualBoard Support" at the bottom left of your screen. A technical representative will reply promptly during normal business hours (within 24 hours of your request).

One other thing -- typos don't matter, we all make them!

DAY 1:

Health and Well-being Concerns

- Please take a moment to introduce yourself. Your first name only is fine. I'd like to know where you live (city, state), and who all lives at home with you, if anyone.
  - Do you have any children who live with you?
  - Do you have any elderly people who live with you, or who you take care of?

- When it comes to your health, what sorts of things are you concerned about? Please describe all of the concerns that you think about, specific to your own health, from time to time.

- What kinds of actions or precautions do you take to address these concerns, if any? Please explain for each health concern that you named.

- What about your family's health – what heath concerns, if any, do you have for others in your family? Please explain in detail.

- What kinds of actions or precautions do you take to address these concerns, if any? Please explain for each health concern that you named.

Sources of Health and Medical Information and Advice

- When it comes to your personal health, where do you turn for information and advice? Please list all of the sources you turn to.

- Who or what do you trust when it comes to health or medical advice? What makes this source/these sources trustworthy? Please explain in detail for each one.

- Who, if anyone, do you trust about health and medical issues aside from doctors or nurses? Please explain what makes you trust each of these people.

- Do you ever do your own research on health topics? If so, where do you start and what sources do you like to use? Please walk me through your process, in detail.

- What happens when you come across health-related information that contradicts other sources of health information? How do you decide what to believe or what to act upon? Please explain in detail, and share examples that may come to mind.

- Do you have a personal or family physician that you go to? If so, what are your thoughts about your doctor? Please describe your level of comfort sharing health concerns with your doctor.

  - If you do not have a regular family doctor or primary care physician, where do you go for medical advice or for medical treatment?

- How would you describe your level of trust with your personal physician? Why is that? Please explain your answer.

- Are there times you find your doctor's advice convincing and other times you question what he or she tells you? Please describe these situations in detail.

- Can you think of a time when you questioned your doctor's advice? Please explain that situation in detail.
  - Did you talk to your doctor about your concerns about this advice?
  - Did you follow the advice anyway, or did you do something else?

- Are there products or home remedies you like to use to stay healthy or to recover from illness? If so, please share examples of these, including when and why you use them.
  - How did you decide that this product or remedy was right for you? Please explain in detail.

Experiences and Attitudes with Respect to Vaccines in General

Welcome back! I'd like to learn about your experiences with getting vaccines, and what you think of vaccines in general. We will talk specifically about the Covid vaccine later in this discussion. For now, I'm interested in **other kinds of vaccines, not the Covid vaccine**, starting with ones you may have gotten when you were a child.

- Think back to when you were a child. Do you recall your parents taking you to get vaccinations – such as vaccinations for measles, tetanus, diptheria, etc.? What do you remember about these experiences?
  - Were these vaccines required where you grew up?

- For those of you with young children, have you taken them to get the standard vaccines for their age group?
  - What are your thoughts about these recommended vaccines for children?
  - How important do you find such vaccines to be?

- As an adult, which vaccines have you received (other than the Covid vaccine)? Please list them, and explain why you chose to get each one.

- How necessary have you found flu vaccines to be for your own health and well-being during the flu season?

- How regularly have you gotten flu shots in recent years?

- If you get the flu shot some years, but not others, what is it that makes you decide to get it some years, but not others?

  - If anything prevented you from getting a flu shot in past years, please explain what it was?

- How much do you typically worry about getting the flu?

- Is there anything that makes you hesitant to get a flu shot? If so, what? Please explain in detail.

- Have you ever had a bad experience with a vaccine? If so, please describe what happened in detail.

- Have you read or heard anything in the past about the safety of vaccines (not including the COVID vaccine) or the need for vaccines, in general? If so, please explain in detail what the safety concerns are that you have heard or read.

  - Where did you hear or read this information?

  - How credible did you find this information about the safety of vaccines or the need for vaccines? Why is that?

  - What, if anything, did you do to try to confirm what you heard or double-check the information?

- Have you ever heard contradictory messages on the safety of vaccines? For example, some sources saying that they are safe and others saying that they are unsafe? Please explain.

- Where or from whom do you usually get your news or information on vaccines? Please be as specific as possible.

  - How much do you trust these sources of information? Please explain why you may or may not trust each one.

- What social media platforms, if any, do you use regularly?

- What impact do you think these social media platforms have had on your feelings and concerns around vaccines? Please explain and use examples, if relevant.

- Do you know of or follow any social media groups that have been talking about vaccines, or sharing information about them?

  - If so, what do you think about the information shared in these groups? How do these groups make you feel?

- When it comes to the need for vaccines and the safety of vaccines, how do you decide which side of the debate to trust? Please explain in detail.

- How do you decide if someone talking about vaccines is an expert in this area or not?
    - What makes someone an expert in the area of vaccines and vaccine safety? Please explain your opinion in detail.
- For those of you with a primary care physician, do you consider that doctor to be an expert on vaccines? Why or why not?
- Are there some vaccines you trust and others you don't? If so, how do you decide? Please explain in detail.

That's all of the questions for today. Thank you for sharing your thoughts and opinions so far! I look forward to learning more from you in the next session!

DAY 2:

Welcome back! At this point, I'd like to turn our conversation to the COVID vaccines that are available.

When it comes to advice and information on Covid vaccines, you may be hearing different things from many different sources – from news stations, local health officials, national health officials, church leaders, community groups, community leaders, websites, and YouTube channels, among others. We'd like to explore which of these sources of information you rely on or trust more and which you rely on or trust less.

- What kinds of things have you heard about the COVID-19 vaccines that have been developed?
- Have you heard or read anything that makes you concerned regarding the safety or the effectiveness of any of the new COVID vaccines?
    - If so, what specifically have you heard that concerns you? Please explain in detail, and give examples.
    - Where did you hear or read this?
- What about the information or source made you trust it?
- What most frightens you about getting a COVID vaccine?
- Which to you is more frightening: getting a Covid vaccine or getting the COVID-19 virus itself?

- How frightened are you that you might pass Covid along to people you care about?

- Does what you've heard make you think you might want to get the vaccine eventually – if your concerns are addressed? Why or why not?

- MULTIPLE CHOICE. Based on what you have seen and heard about the Covid vaccines so far, which of the following describes your attitude towards getting a Covid vaccine?

  - ❑ I will definitely get it as soon as I can.
  - ❑ I will probably get it as soon as I can.
  - ❑ I will probably get it eventually but want to wait until I know it definitely works.
  - ❑ I will probably get it eventually but want to wait until I know it is safe.
  - ❑ I will probably not get it.
  - ❑ I will definitely not get it.

- How necessary is getting vaccinated in preventing you from getting sick or dying from Covid?

- How effective do you think the vaccine would be in protecting you from Covid?

- Are there other, practical reasons that are preventing you or people you know from getting a Covid vaccine, or making it harder to do so?

- Do you have people in your family or community who feel they may not be eligible to get a Covid vaccine? If so, why do they think they wouldn't be eligible?

- Who else is involved in your decision and ability to get a Covid vaccine? Would you need to get permission from anyone else in the family before you are could get a vaccine?

- Is there disagreement within your family – some family members who want to get a Covid vaccine and others who don't? If so, how will that be resolved?

- Have you ever discussed the Covid vaccine or expressed your concerns about it with a medical professional, such as a doctor or pharmacist? If not, why not? If so, did they advise you to get a vaccine? What reasons did they give?

- Do you know people who have been vaccinated against Covid? Do you have friends who have been vaccinated? If so, how has this affected your thinking about the vaccine?

- If you have a church you belong to, have church leaders made any recommendations about the Covid vaccine? If so, what have they recommended?

- How important to you is the advice of church leaders on this subject?

- How do you weigh their recommendations against those of health professionals if they contradict one another?

- What about local community groups? What kinds of messages have you heard from community centers or other local organizations about the vaccine?

- How important or relevant to you are their recommendations in this regard?

- What kind of things do you hear from people in your neighborhood/community? Are many people in your community outspoken about the Covid vaccine and whether or not it is safe?

- And what about social media, what do you hear about the Covid vaccine on the social media sites you use?

- How much do you trust things people are posting on social media about the Covid vaccine?

- Can you give an example of a source you trust on social media?

- What about the Pharmaceutical companies that developed the Covid vaccines – how much do you generally trust their products?

- Have you heard anything that makes you question their development of the Covid vaccines?

- What about your local or state public health officials? What has been their advice regarding the Covid vaccines?

- Do you find their statements trustworthy or re-assuring? Why or why not?

- Do you find advice from local health officials to be more relevant or compelling than advice from national health officials? Why or why not?

- How much do you trust the recommendations or advice of public health leaders in the government, such as Dr. Anthony Fauci, Director of the National Institute of Allergy and Infectious Diseases and member of the White House Coronavirus Task Force?

- What about the Centers for Disease Control (CDC)? How familiar are you with this organization?

Scales D, *et al. BMJ Open* 2023; 13:e072619. doi: 10.1136/bmjopen-2023-072619

- Had you heard of it before the Covid pandemic?

- Do you trust its recommendations on how to protect yourself against Covid?

- Has your trust in the recommendations of the CDC changed over the course of the pandemic? Is so, how?

- How important is it to you to know that the FDA (the U.S. Food and Drug Administration) had authorized the new vaccine to feel it would be safe for you to get?
  Does this reassure you as to the safety of the Covid vaccines?

- Have you heard anything that makes you question the FDA's authorization of the Covid vaccines?

- Are there any reasons why you find government reassurances about health issues like Covid to be unconvincing?

DAY 3:

- How has your community been affected by Covid?

- How has Covid affected the neighborhood you live in? Has it seen a lot of cases?

- Have you heard of people around you getting seriously ill or dying from Covid?

- How has your family been affected? Do you have family members or close friends who have gotten seriously ill from Covid? How does this affect your thinking about the value of getting a vaccine?

- How worried are you about catching Covid? Does the idea of getting Covid frighten you?
- How frightening is the idea of your parents or other older family members getting it?

- Have you caught Covid? If so, how bad was it? If so, how does this make you feel about getting vaccinated?

- How do you feel that being Black/African-American affects your risk of catching Covid or of having serious reactions to Covid?

- Do you know of people here who want to get a Covid vaccine but are having trouble getting it?

- How does this influence your thinking about the Covid vaccine?

- Does this make you feel lucky or privileged to live in a country and a time when it is possible to be vaccinated against a potentially deadly disease?

- How much do you feel that it is a privilege to be able to get vaccinated, given that there are many countries where people desperately want it and can't get it.

- Do you have any religious objections to the vaccines? Has any clergy person discouraged you from getting vaccinated? IF SO, PROBE RE: nature of the objection or the reason for the discouragement

- How important do you feel it is to sooner or later get a Covid vaccine for your own health?

- How important do you feel it is to get a vaccine to help protect your family members or others you come in contact with who might be very vulnerable to having a serious reaction to Covid?

- How important do you feel it is for people to get a Covid vaccine to help restore normalcy??

- Do you think certain people should be required to get them? Why or why not?

- Do you think everyone should be required to get them? Why or why not?

- Who or what do you think would convince you that a Covid vaccine is safe?

- What are the best arguments you have heard for getting the vaccine?

- Even if you don't think you personally need it, are there other reasons you might want to get it? If so, please explain.

- To what extent should it be a matter of individual need or choice versus a matter of community responsibility?

- What would make you feel better or more confident of the safety of Covid vaccines?

- Whose endorsement of vaccination would you need to see?

- Would it have to be a leader in your community? Someone in your family? Someone in your church?

- As an African American, whose endorsement of a Covid vaccine would carry the most weight for you? Is there an individual or a group or organization that place a great deal of trust in with regards to this issue?

- Now I'm going to present some facts about the Covid vaccine, and I'd like to get your reaction acts and hear how these facts might affect your thinking.

- How does it affect your thinking about Covid vaccines that it helps protect the most vulnerable family members and community members?

- How does it affect your thinking about Covid vaccines that doctors and healthcare workers trust the vaccine and are getting them.

- How does it affect your thinking that hundreds of millions have now gotten the vaccine safely?

- How does it affect your thinking to see that getting vaccinated is helping us get our normal lives back, and ending social isolation?

- What part do you think vaccination should play in helping us open up and return to normal, and eliminating ongoing fear of Covid.

Thank you all for your participation and for sharing your thoughts. Is there anything else about the Covid vaccine that you would like to share before we close?

Scales D, *et al*. *BMJ Open* 2023; 13:e072619. doi: 10.1136/bmjopen-2023-072619
