# Supplementary material for: ‘They’ve all endorsed it…but I’m just not there:’ a qualitative exploration of COVID-19 vaccine hesitancy reported by Black and Latinx individuals
Source: BMJ Open. 2023 Jul 20;13(7):e072619. doi: 10.1136/bmjopen-2023-072619 (PMC10360437; doi:10.1136/bmjopen-2023-072619)
Supplement: Author's manuscript [file bmjopen-2023-072619.draft_revisions.pdf]

BMJ Open is committed to open peer review. As part of this commitment we make the peer review history of every article we publish publicly available.

When an article is published we post the peer reviewers' comments and the authors' responses online. We also post the versions of the paper that were used during peer review. These are the versions that the peer review comments apply to.

The versions of the paper that follow are the versions that were submitted during the peer review process. They are not the versions of record or the final published versions. They should not be cited or distributed as the published version of this manuscript.

BMJ Open is an open access journal and the full, final, typeset and author-corrected version of record of the manuscript is available on our site with no access controls, subscription charges or pay-per-view fees (http://bmjopen.bmj.com).

If you have any questions on BMJ Open's open peer review process please email info.bmjopen@bmj.com

BMJ Open

"They've all endorsed it…but I'm just not there:" A qualitative exploration of Covid-19 vaccine hesitancy reported by Black and Latinx members of online bulletin board groups

Journal:	*BMJ Open*
Manuscript ID	bmjopen-2023-072619
Article Type:	Original research
Date Submitted by the Author:	09-Feb-2023

Complete List of Authors:	Scales, David; Weill Cornell Medical College; Critica<br>Gorman, Sara; Critica<br>Windham, Savannah; Critica<br>Sandy, William; Fluent Research<br>Gregorian, Nellie; Fluent Research<br>Hurth, Lindsay; Critica<br>Radhakrishnan, Malavika; Critica<br>Akunne, Azubuike; Critica<br>Gorman, Jack M.; Critica

Keywords:	COVID-19, Anthropology < TROPICAL MEDICINE, Patient Participation, PREVENTIVE MEDICINE

SCHOLARONE™ Manuscripts

I, the Submitting Author has the right to grant and does grant on behalf of all authors of the Work (as defined in the below author licence), an exclusive licence and/or a non-exclusive licence for contributions from authors who are: i) UK Crown employees; ii) where BMJ has agreed a CC-BY licence shall apply, and/or iii) in accordance with the terms applicable for US Federal Government officers or employees acting as part of their official duties; on a worldwide, perpetual, irrevocable, royalty-free basis to BMJ Publishing Group Ltd ("BMJ") its licensees and where the relevant Journal is co-owned by BMJ to the co-owners of the Journal, to publish the Work in this journal and any other BMJ products and to exploit all rights, as set out in our licence.

The Submitting Author accepts and understands that any supply made under these terms is made by BMJ to the Submitting Author unless you are acting as an employee on behalf of your employer or a postgraduate student of an affiliated institution which is paying any applicable article publishing charge ("APC") for Open Access articles. Where the Submitting Author wishes to make the Work available on an Open Access basis (and intends to pay the relevant APC), the terms of reuse of such Open Access shall be governed by a Creative Commons licence – details of these licences and which Creative Commons licence will apply to this Work are set out in our licence referred to above.

Other than as permitted in any relevant BMJ Author's Self Archiving Policies, I confirm this Work has not been accepted for publication elsewhere, is not being considered for publication elsewhere and does not duplicate material already published. I confirm all authors consent to publication of this Work and authorise the granting of this licence.

For peer review only

“They’ve all endorsed it…but I’m just not there:” A qualitative exploration of Covid-19 vaccine hesitancy reported by Black and Latinx members of online bulletin board groups

David Scales,[1,2] Sara Gorman,[2] Savannah Windham,[2] William Sandy,[3] Nellie Gregorian,[3] Lindsay Hurth,[2] Malavika Radhakrishnan,[2] Azubuike Akunne,[2] & Jack M. Gorman[2]

1. Section of Hospital Medicine, Division of General Internal Medicine, Department of Medicine, Weill Cornell Medicine, New York, NY, United States
2. Critica, Bronx, NY, United States
3. Fluent LLC, New York, NY, United States

Word Count: 4994

Corresponding Author:
David Scales
david.scales@aya.yale.edu
525 East 68th Street
New York, NY 10068

Acknowledgments

This work was supported by the Robert Wood Johnson Foundation (grant numbers 76935 and 78084) and Weill Cornell Medicine's JumpStart program (grant number N/A). The opinions expressed in this piece do not necessarily reflect those of either the Robert Wood Johnson Foundation or Weill Cornell Medicine.

Conflicts of Interest
The authors state that they have no conflicts of interest to declare.

For peer review only

Abstract

Objective We sought to examine an in-depth analysis of reasons for vaccine hesitancy among online communities of US-based Black and Latinx communities to understand the role of historical racism, present-day structural racism, medical distrust, and individual concerns about vaccine safety and efficacy.

Design A qualitative study using narrative and interpretive phenomenological analysis of online bulletin board focus groups.

Setting Bulletin boards with a focus-group like setting in an online, private, chat-room-like environment

Participants Self-described vaccine hesitant participants from US-based Black (30) and Latinx (30) communities designed to reflect various axes of diversity within these respective communities in the US context.

Results Bulletin board discussions covered a range of topics related to COVID-19 vaccination. Covid-19 vaccine hesitant participants expressed fears about vaccine safety and doubts about vaccine efficacy. Elements of structural racism were cited in both groups as affecting populations but not playing a role in individual vaccine decisions. Historical racism was infrequently cited as a reason for vaccine hesitancy. Individualized fears and doubts about Covid-19 (short- and long-term) safety and efficacy dominated these bulletin board discussions. Community benefits of vaccination were not commonly raised among participants.

Conclusions While this suggests that addressing individually-focused fear and doubts are central to overcoming Covid-19 vaccine hesitancy in Black and Latinx groups, addressing the effects of present-day structural racism through a focus on community protection may also be important.

Keywords
COVID-19 Vaccines, Vaccination Hesitancy, Black, Latinx, Infodemic, Pandemics

Strengths and limitations of this study

- We employed online bulletin board groups of US-based Black and Latinx participants selected for self-described vaccine hesitancy to understand the resons and motivations behind their stance, leveraging the online design to attract participants whose information environments consist of high proportions of online content where anti-vaccine misinformation was prominent.
- Compared to in-person focus groups, asynchronous bulletin boards allow all participants to freely express themselves with fewer concerns about speaking too much while facilitators can encourage greater participation from those who are more quiet.
- Transcripts were analyzed using various qualitative techniques, including narrative and interpretive phenomenological analysis to allow for understanding recurrent themes.
- Participants revealed that vaccine hesitancy is the result of a confluence of psychological and social considerations, but with selective focus on certain factors over others as participants weighed risks and benefits, such as high emphasis was placed on individual vaccine safety with relatively little attention to potential community-level benefits of vaccination.
- Vaccine safety and efficacy were of highest concern; however, distrust in institutions and concerns about systemic and personal racism also featured prominently among participants' concerns.

Introduction

For much of the COVID-19 pandemic, rates of COVID-19 vaccination in Black and Latinx communities in the United States were lower than White communities, although the gap appears to be narrowing (1–3). This vaccination gap is especially concerning because Black and Latinx people diagnosed with Covid-19 have experienced worse clinical outcomes (4). Social determinants of health make it difficult for people in some communities who want to be vaccinated to get vaccines, but even as vaccine uptake gaps have narrowed, a substantial number of people continue to choose not to be vaccinated (5–7).

Covid-19 vaccine hesitancy among Black and Latinx people has been found in survey studies to be higher than among White people (8–10). In their review of 13 studies of racial and ethnic disparities in Covid-19 vaccination status, Khubchandani and Macias[9] found an overall pooled rate of vaccine hesitancy of 26.3%, but a higher rate among Hispanic (30.2%) and African American (40.6%) study participants (11). Survey data have shown that concerns about vaccine safety and efficacy are associated with higher rates of vaccine hesitancy among Black and Latinx people (12,13). Kricorian and Turner identified lack of trust in healthcare providers and the healthcare system as major factors in Covid-19 vaccine hesitancy among these groups (14). Even among healthcare workers, Covid-19 vaccine hesitancy was found to be higher among Black and Latinx people compared with White people (15).

Research has further delineated some of the underlying reasons for Covid-19 vaccine hesitancy among Black and Latinx people. Longoria and colleagues found fears about Covid-19 vaccine safety were commonly circulated online among Latinx people, as well as narratives about alleged “alternative treatments” (16). An analysis of online posts found that mistrust of vaccines and the motivations of official institutions were commonly expressed in online platforms viewed by the Black community (17). Similar concerns were heard in focus groups of Black salon and barbershop owners (18). In addition to these fears, Bateman and colleagues conducted virtual focus groups and identified mistrust of the Covid-19 vaccine development

process and of politicians and historical mistrust of the way Black people have been treated by the healthcare system as factors for vaccine hesitancy among Black and Latinx participants from the Deep South (19). Another focus group study with Black and Latinx community members also identified “pervasive mistreatment” as a basis for vaccine hesitancy in those communities (20).

In particular, current strains of research highlight that perspectives about racism in medicine are not simply about past narratives around Henrietta Lacks or the unethical syphilis experiments performed in Tuskegee but related to contemporary lived experiences (21). Using survey data, Martin, Stanton and Johnson (22) found that current mistreatment by the healthcare system, rather than historical mistreatment as exemplified by the Tuskegee experiments, was associated with Covid-19 vaccine hesitancy among Black Americans. A survey study of people in underserved communities in North Carolina identified safety concerns and government mistrust as the most important factors for vaccine hesitancy among Black and Latinx respondents (23). In a survey using U.S. census data, Black people were more likely than white people to develop Covid-19 vaccine hesitancy because of lack of confidence in the safety and efficacy of vaccines and because of a tendency to watch evolving information and wait before considering vaccination, though this group saw the greatest percentage drop in hesitancy over time (24). Recent work by Morales and Paat (2022) provides additional evidence of a “watch and wait” approach among Black Americans, noting how rates of vaccine hesitancy and refusal in this community declined over time while it remained stable in White communities (25).

Additionally, healthcare access disparities remain an important issue that intersects with manifestations of medical racism within the healthcare system. For various geographic and socioeconomic reasons, Black people are less likely to have access to a primary care physician (26–28) and more likely to use emergency care, a relationship partly mediated by mistrust in the healthcare system (27). Therefore, while primary care physicians are often cited across different racial and ethnic groups as the most trusted person when it comes to vaccine decision-making

(29,30), structural inequalities in healthcare access means that many marginalized communities lack access to these heavily trusted sources (31,32). Some population-based studies support the link between access to primary care and those provider recommending vaccines to higher rates of Covid-19 vaccine uptake (33,34).

The literature on Covid-19 vaccine hesitancy among Black and Latinx people thus highlights three broad factors that influence vaccine decisions: historical mistreatment in medical/scientific studies or contexts (e.g., the US Public Health Service syphilis study at Tuskegee), current mistreatment by the healthcare system (including structural racism), and individual fears and concerns about vaccine adverse side effects and efficacy, which can change over time. We wished to probe more deeply into these factors to get a better understanding of the complexities involved in lower Covid-19 vaccine update among Black and Latinx people.

We conducted two separate online bulletin boards, one with participants from each community who self-identified as vaccine hesitant, to probe the factors behind their vaccine decisions. We were interested in getting an in-depth understanding of what motivates people in the Black and Latinx communities to be Covid-19 vaccine hesitant. Originally, we intended this as two separate inquiries, one involving Black and the other involving Latinx participants and therefore the designs and recruitment strategies of the online bulletin boards were different. However, we observed remarkably similar responses from participants in the two groups and therefore decided to combine them into a single report.

These bulletin boards were conducted soon after Covid-19 vaccines were made available and reflect attitudes at that time. Since then, gaps in Covid-19 vaccination rates among racial and ethnic groups have narrowed and therefore it is likely that attitudes about them have also changed. However, the results of these bulletin boards remain important for two reasons: first, because they tell us that fears about vaccine safety and efficacy are important

drivers of vaccine hesitancy in Black and Latinx communities; and second, because they may help inform future vaccine uptake strategies as new healthcare challenges inevitably arise.

Methods

We conducted two bulletin boards from July 13 to 22, 2021, following the Covid-19 vaccine rollout in the U.S. Informed consent via an online form was obtained from each participant prior to the start of the study, and they were assured that participation was voluntary. Participants were told that they could end their participation at any time and were free to leave any questions unanswered. Subjects were paid $120 for their participation. This research was deemed exempt from IRB review by Ethical and Independent Review Services and approved by the [redacted for peer review] IRB. Due to privacy concerns, data are not currently publicly available, but de-identitified data can be obtained by researchers on a case-by-case basis by contacting the authors.

A bulletin board is an asynchronous online discussion involving greater numbers of individuals than typical focus groups and taking place over an extended period (35–37). Participants log into a password-protected site to answer questions that are posted and monitored by a moderator. The moderator can also follow up on responses for clarification or elaboration. The bulletin board is a flexible research tool that allows the moderator to post questions and probe any individual participant following their entry. The respondents can take as much time to respond as they need. Individual responses are initially uninfluenced by the group, as participants do not see other responses to any given question until they have posted their own response. This method helps to minimize the social desirability bias (38) that may influence participants after exposure to another's responses.

Data collection. Participants in the bulletin board with Black participants were recruited from a panel of people who have previously agreed to participate in online surveys. They were

For peer review only - http://bmjopen.bmj.com/site/about/guidelines.xhtml

contacted by email with an invitation to participate. If interested, they were asked to respond to an online screener that assessed their level of vaccine hesitancy. People who had already been vaccinated, intended to be vaccinated soon, or who adamantly opposed Covid-19 vaccination were excluded, leaving participants who showed some level of hesitancy about having a vaccine. If qualified, they provided their contact information and were given instructions for logging into the bulletin board. Demgraphics of the participants can be found in Table 1.

The process for recruiting participants from Latinx communities differed from that for Black participants. We posted invitations in Spanish on various Facebook pages created for Latinx sub-populations, such as groups for communities from Peru, Colombia, Mexico and the Dominican Republic. If interested, they were asked to complete the online screening questionnaire in Spanish, to determine if they met the criteria for participation, which were the same as for the participants in the Black groups as described above. If qualified, they provided their contact information, and were supplied with instructions for logging into the bulletin board. This project was designed to inform subsequent interventions to address vaccine hesitancy. Therefore, the demographics of each group were chosen to approximately match those of the groups in which interventions would take place in a later study. Similarly, our funding for this project came from a source focused exclusively on health in the United States, we focused on finding participants that were relatively representative of these racial and ethnic groups living in the US.

When participants logged into the bulletin board, they were presented with an introduction from the moderator, a review of the process, and a reminder that they were not obliged to answer any question. They were reassured that the research was anonymous and their identities, including contact information, would not be shared. The moderators of the bulletin boards introduced themselves at the outset and posted their photographs so that the respondents could see them. Participants were allowed to post photographs of themselves to the group, though this was not required.

Participants were then presented with the first of a series of questions. Only after a participant entered their response to a question were they able to see how other participants responded to that same question. At this point, they were free to respond to what other participants had said. After responding to all of the questions posted for that day, they were reminded to check back periodically to respond to possible follow-up questions posted by the moderator. This process continued over three days, with a different set of questions posted each day.

Bulletin board questions were designed for flexible, open-ended inquiry. The research did not seek to confirm any hypotheses but rather to explore the range of perceptions and attitudes that exist in the vaccine hesitant population and to identify important influencers of those perceptions and attitudes, including trusted sources of information, media outlets, social networks, community leaders, health professionals, etc. Examples of the various topics of inquiry and discussion can be found in Table 2. We also asked participants' perspectives on influenza vaccines, but only data from questions about Covid-19 vaccines are included here.

Data quality control. The study employed purposive sampling with screening to ensure that respondents reflected the target population in terms of attitudinal, behavioral, and demographic characteristics. The sample was highly diverse with respect to age, geography, socio-economic status, and in the case of the Latinx sample, with respect to both level of acculturation to the U.S. and national heritage (see Table 1).

The bulletin boards were conducted by trained moderators, each with 20+ years of qualitative research experience. The Latinx bulletin boards were conducted in Spanish by a Latinx moderator; the Black bulletin boards were conducted in English by a Black moderator. The Spanish-language discussion among Latinx respondents was translated into English by an automated translation program provided by the online platform. This was done for the benefit of those observing the discussion who were not Spanish speakers. The automated translation was

review only

not used, however, for the purposes of reporting due to some translation errors. Transcripts included in the report were translated by professional Spanish-speaking moderators.

Methods of analysis. A combination of methods was employed in the analysis of the content generated by these bulletin boards, including interpretive phenomenological analysis (IPA), narrative analysis, and qualitative content analysis. These methods enabled us to explore how respondents narrate and make sense of their prior experiences with vaccines, with medical professionals, and with various sources of medical and health-related information. They also enabled us to observe how participants rationalize their hesitancy with respect to Covid-19 vaccination, and to identify a range of social, emotional, and perceptual barriers to vaccination. Analysis enabled us to identify the range of opinions exhibited, opinions that are universally shared and those that are more idiosyncratic and portray how different perceptions tend to be clustered or coupled.

Interpretive phenomenological analysis (IPA). Data collection was not designed to test hypotheses or preconceptions, nor was data analysis. The intent was to use the data gathered to better understand the experiential world of the respondents, how they understand the phenomenon of the ongoing pandemic, and how they rationalize their decision to refrain from vaccination. Through this bottom-up analysis, we sought commonalities and patterns in experiences and shared forms of reasoning to inform a richer understanding of vaccine hesitancy. In addition, the analysis included any consistent variations in participants' responses that corresponded with major demographic variables such as gender and age. For the descriptive analysis, we identified and cataloged the fullest possible range of opinions around vaccine hesitancy, including commonly cited sources of information, facts, anecdotes, and trusted sources of information, regarding the pandemic and Covid-19 vaccines.

For peer review only

Narrative analysis. In addition to identifying and cataloging the range of opinions and perceptions articulated by participants, the analysis focused on identifying the ways in which information has been woven into narratives. This analysis focused on participants' descriptions of their experiences during the pandemic, their methods for searching for and processing relevant information, and the stories they tell themselves about the need or lack of need for a vaccine. In addition, we analyzed the trajectory of each individual participant's experience with vaccines, looking to identify key moments when their attitudes reportedly changed. This analysis also sought to identify pre-existing narratives and how those intersect with participants' narratives about the pandemic, such as mistreatment of marginalized populations by the healthcare system and lack of trust in the government. This analysis also attempted to gauge the extent to which participants' narratives are fixed, are still being formed, or remain open to revision.

Patient and Public Involvement

None.

Results

We conducted one bulletin board with 30 people from the Black community and one with 30 people from the Latinx community. Characteristics of the participants can be found in Table 1. The themes obtained from the bulletin boards about Covid-19 vaccines in both the Black and Latinx groups were remarkably similar and therefore we combined them in this section. The analysis suggests several interrelated barriers to Covid-19 vaccination are at work in both Black and Latinx communities, strongly influencing vaccine behaviors in these populations. Five main themes and several sub-themes emerged. Illustrative quotations can be found in Tables 3, 4, and 5:

1. **Safety concerns (Table 3)**

For peer review only

- Vaccine unknowns. Vaccines are a “black box.” Some participants perceived vaccine ingredients to be elusive or intentionally obscured with mysterious ingredients.
- Fears about Covid-19 vaccine safety. Participants expressed many fears and doubts regarding both the short- and long-term safety of the vaccines; even those who express high trust in doctors and science and low trust in social media still say stories of vaccine-induced illness make them highly uncertain.
- Conviction that the Covid-19 vaccine can kill you. Some participants believe that the vaccine is directly responsible for deaths.
- Concerns about scientific uncertainty. Public scientific debates about vaccine safety and adverse side effects instill and perpetuate doubts by creating the appearance of scientific uncertainty even among those who normally trust medical professionals. Many seem to almost throw up their hands and say, “I can’t decide what’s true and what’s not, so best to do nothing,” or to wait for more conclusive information.

2. Skepticism about vaccine efficacy (Table 3)

- Covid-19 vaccines are not effective. Several stories about new variants, breakthrough infections, and surging cases suggested a belief that the vaccine would not be effective in protecting them.
- Covid-19 vaccines are insufficient. Even with an effective vaccine, mass vaccination is not enough to return life to normal and that Covid-19 is here to stay, implying that the vaccine’s benefits may be exaggerated.
- Covid-19 vaccines do not prevent transmission. Although vaccines reduce the risk of transmission, news that people can still pass Covid-19 on to others even after being vaccinated is conflated with a narrative that vaccines do not work as intended, thus undermining the argument for getting it to protect others.

For peer review only

3. **Risk/benefit calculations were not perceived to favor vaccines (Table 4)**

- Vaccines are riskier than the virus. Participants frequently assigned greater risk to the vaccine than to the virus itself and noted that there are other ways to prevent infection (like masking), so on balance the vaccines are felt to be unnecessary.
- Covid-19 vaccines are not necessary. Participants in both groups often believed they were not at risk of dying from Covid-19; they believed they could contract the virus and recover from it. They also believed that any illness would be mild, underscoring a lack of urgency to be vaccinated.
- Covid-19 vaccines are only for the most vulnerable. Vaccines are for the most vulnerable, such as older people and immunocompromised people, not the young and healthy, or those being careful and taking other precautions.

4. **Limited trust in institutions (Table 5)**

- Limited trust in physicians. Many say that they trust their primary care doctors the most when it comes to their health, but that trust does not always extend to advice about the Covid-19 vaccines; they do not necessarily see their doctors as experts in this regard. For instance, some seem to say, "at this point, no one can claim to be an expert on these vaccines. So, no one can truly tell me what is best."
- Lack of trust in government. There is a lack of trust in government in general and especially in government spokespersons, undermining their authority as credible messengers. Many tune them out or do not lend them credence, even those who otherwise trust their doctors and medical professionals. Some people suggest that the very fact that the government so badly wants them to get a vaccine makes them not want to get it.

For peer review only

- Limited trust in public health authorities. While some of the vaccine hesitant respondents expressed very high regard for medical professionals and for public health authorities in general, they were more critical of public agency-relayed information about Covid-19 vaccines. Some argued that public health authorities only say what they are told to say by the administration. Some participants distrust the CDC, largely because they viewed the agency as frequently changing its advice and guidelines.

5. How health outcomes differ by race and/or ethnicity (Table 5)

Participants in both groups perceived structural racism as factors that influence a group's risk of infection and the likelihood of having access to vaccines. However, the participants did not cite structural issues as influencing either their own personal risk of infection or their decision to be vaccinated. Two people mentioned the Tuskegee experiments (39) as indicative of abuses against Black people by the healthcare system and a reason to be wary of healthcare system programs, including Covid-19 vaccines. The potential benefits of vaccination, such as protecting vulnerable communities, were not raised as a motivation for vaccination.

Other observations. Although themes and sub-themes about vaccine hesitancy were quite similar between the Black and Latinx groups in this study, there was one notable difference. Black participants were more likely to emphasize obtaining information about Covid-19 vaccines from the internet, despite having what appears to be strong relationships with medical providers. Latinx participants also had strong relationships with and trust in medical providers and seemed to make less use of the internet for health information. Both groups rely heavily on trusted friends and relatives for health information. Even with that support, however, moving to vaccine acceptance for some people can be very difficult and take more time than for others. As one Black participant noted: "*I love and trust my family; I love and trust my pastor. And they all made*

For peer review only

their position known. And I know none of them do things haphazardly… [but] the jury is still out for me… I'm just straddling the fence, and it's just a personal thing with me… They've all endorsed it, my pastor endorsed it…but I'm just not there, I'm not."

It should be noted that participants did not endorse conspiracy theories or unsubstantiated notions about vaccines (e.g., that they contain microchips) that have been voiced in anti-vaccine channels.

Discussion

The results of this research suggest that interrelated barriers to vaccination are at work in communities of color and strongly influence Covid-19 vaccine behaviors in these communities. Two main sets of concerns emerged from in these bulletin boards: that the vaccines are unsafe and that they are insufficiently effective. These concerns are remarkably similar to those observed in an earlier bulletin board study that involved a group of participants that had a majority of white people (40). Indeed, these may be ubiquitous influences on vaccine hesitancy across racial, ethnic, and national groups (41–43).

Our impression is that most of the basis for Covid-19- vaccine hesitancy in both groups in this study is fear and skepticism and that this fear and skepticism are part of the confirmation bias loop that is enabled by our information environment that emphasizes individual freedom and minimizes collective responsibility (44,45). It is important to note that despite journalistic coverage of new information about the vaccines, like breakthrough infections, viral variants, and the endless reporting of rare vaccine adverse side effects, reinforced fear in spite of the fact that the scientific evidence and consensus at the time of these bulletin boards was both that the vaccines were safe and they were highly effective at preventing morbidity and mortality due to Covid-19. This means that the concerns that were circulating in these communities then met most definitions of scientific misinformation (46,47).

For peer review only

Participants seemed eager to make clear they felt race and ethnicity are factors in community viral infection susceptibility because of the history of structural racism in healthcare and medicine. Lack of access to healthcare and to vaccination sites has been found to be a factor in limiting vaccination among Black and Latinx people (20). The history of racism and medical experimentation on people of color in the United States was cited as among the reasons for vaccine skepticism among Black participants in one recent study (48). However, as in the survey study of Martin, Stanton, and Johnson (22), participants in our study did not frequently express a conviction that historical racism was a factor in their *personal* decisions about vaccination. Two of the 30 participants in our Black participant group directly named the Tuskegee experiments, a hallmark of unethical, racist scientific and healthcare practices in the U.S. Thus, although participants in both groups often cited examples of structural racism in general, they were more likely to express individual feelings of fear and skepticism about the vaccines as the main factors in making them hesitant to be vaccinated.

Historical traumas like the experiments that took place in Tuskegee may still have an effect on people's attitudes and decision making even if not explicit (49,50). Current experiences with racism such as health outcome disparities may be as or even more important in shaping ways that people of color make decisions about healthcare issues like vaccination (51). It is possible that the way we framed questions in these bulletin boards influenced participants toward speaking more about their individual concerns as the main factors in Covid-19 vaccine decision making and away from broader discussions about the impact of historical and present racism on those decisions. It is also possible that for these participants at least, while recognizing that structural factors like crowded work conditions and lack of healthcare access make communities of color more likely to acquire Covid-19 and to have more negative outcomes, individual fears and skepticism about the Covid-19 vaccines were indeed the most pressing concerns that influenced vaccine hesitancy.

For peer review only

Although participants expressed trust in their own personal healthcare providers, they exhibitied a general lack of trust in agencies and institutions that are charged with the responsibility of informing and reassuring the public about vaccine safety and efficacy. This is not a unique finding. Previous studies have found people in Black and Latinx communities have low levels of trust in the healthcare system (26), and racial differences in healthcare access has been noted as a contributing factor (27).

Participants in our groups have clearly seen and heard a great deal of information about vaccines, although not all of it is accurate. It seems unlikely that merely supplying more facts about vaccine safety and efficacy will be sufficient to encourage people to get vaccinated. Some things that may be more likely to convince vaccine hesitant people to be vaccinated include:

- Creating stronger incentives – incentives that act to both ‘push’ and ‘pull’ the vaccine hesitant. Disincentivizing staying unvaccinated and offering financial or material benefits for vaccination such as being required to be vaccinated in order to fly to see family abroad, to go to public events, to work, to visit relatives in hospitals or nursing homes. Financial incentives could backfire, however, by creating the impression of financial coercion. Lotteries to incentivize vaccine uptake also had mixed results depending on the incentive structure.

- Working to shift perceived social norms. At the moment, social norms are working in favor of the vaccine hesitant, given the continued emphasis on their large numbers of unvaccinated. It may be possible to shift perceived social norms by emphasizing the ever-growing number of vaccinated and ever-shrinking number of unvaccinated to make it seem less normative or by highlighting stories of vaccine hesitant people who decide to get vaccinated.

- Trying to alter the personal risk calculation of the vaccine hesitant by increasing the sense of personal need (to protect one from new strains or to protect loved ones, for example). Fear of a new surge in infections seems to make some people re-assess the risk and become more open to getting a vaccine, although it seems to make others conclude the opposite: that vaccines cannot protect them from an ever-changing virus.

- Addressing the issue of inefficacy, that even vaccinated people are getting Covid-19 or can still infect others. This is frequently cited as the rationale for not being vaccinated since it undermines the rationale of vaccination to protect your loved ones or to end transmission. It also belies the fact that vaccines generally do not prevent a person from being infected with a pathogen except in the cases where immunization programs have led to the elimination or eradication of a pathogen; rather, vaccines prevent people from getting seriously ill.

- Encouraging those who have been vaccinated to reassure unvaccinated family members and friends of how safe it is and how they have experienced no adverse side effects in the months since being vaccinated.

- Explicitly and more emphatically framing both risks of Covid-19 and benefits of vaccination in community-level terms. It is unclear why participants often framed discussion of discrimination at structural and community levels yet did not perceive the negative effects of Covid-19 nor benefits from vaccination in a similar light. This suggests both the success of current messaging on issues of structural racism, yet at the same time the insufficiency of public health messaging to shift newspaper and television news language from individual to risks/benefits for marginalized communities.

For peer review only

- Providing sufficient training to primary care physicians and pediatricians about vaccines and best-practice strategies for counteracting misinformed ideas held about vaccines by their patients. At the same time, it is important to promote the idea that primary care professionals do indeed have sufficient expertise to provide reliable vaccine information.

- Improving access to vaccines for people in traditionally underserved communities. Access issues can create mistrust and suspicion of the healthcare system and efforts to ameliorate them may make some people more likely to accept vaccines (52).

Limitations

This study has several limitations, including inherent self-selection bias in the sample of participants. There is also inevitable bias toward the views of those comfortable sharing their opinions in a group discussion with others in a digital setting where social desirability bias may make some participants reticent to share what may be perceived as outlandish opinions. This may have been a factor in the fact that subjects did not, for the most part, mention historically racist events and the United States' racialized history does not rule out that these are important factors for vaccine hesitancy. We did not ask specific questions about these issues. Participants' reports of mistrust of public health authorities and the government represent the result of both historical racism and personal experiences of racism. Thus, while we can report our observation that for the most part neither people in our Black nor Latinx groups volunteered racism as affecting their own vaccine decisions, deeper probing might have elicited that as an important factor. Indeed, Dong and colleagues conducted semi-structured interviews with 24 Black Americans and reported that, "systemic racism was discussed as the root cause of the different types of mistrust" (53).

For peer review only

In summary, bulletin boards with Covid-19 vaccine-hesitant people from the Black and Latinx communities revealed that the major factors influencing vaccine hesitancy involve fears of lack of safety and efficacy of the vaccines. There is a misperception that not being vaccinated is a social norm because of media emphasis on unvaccinated people. These attitudes are reinforced by a perception of lack of consensus about the vaccines among experts, mistrust of government officials and institutions, and belief that other measures are sufficient to prevent acquisition and spread of Covid-19**.** Future research will focus on strategies to improve vaccine acceptance that do not rely only on providing facts but account also for the anxieties and fears that motivate vaccine hesitancy.

For peer review only

Contributorship Statement

DS and JMG conceived of the study, wrote the protocol, obtained funding and engaged WS and NG to carry out the research and conduct preliminary data analysis. DS, SG, JMG, WS and NG were responsible for inclusion/exclusion criteria of both bulletin board groups. WS and NG were responsible for bulletin board design, and recruitment of participants. WG undertook initial interpretive phenomenological and narrative analysis. SG, SW, LH, MR, AA contributed to further data review and interpretation with DS and JMG. All authors read and critically evaluated multiple drafts of the manuscript before providing final approval of the version to be published.

Competing Interests

The authors state they have no competing interest to declare.

Funding

This work was supported by the Robert Wood Johnson Foundation (grant numbers 76935 and 78084) and Weill Cornell Medicine's JumpStart program (Grant number N/A). The opinions expressed in this piece do not necessarily reflect those of either the Robert Wood Johnson Foundation or Weill Cornell Medicine.

Data Sharing Statement

Due to privacy concerns, data are not currently publicly available, but de-identitified data can be obtained by researchers on a case-by-case basis by contacting the authors. Patients were not involved in the design of the study.

Ethics Approval Statement

This research was deemed exempt from IRB review by Ethical and Independent Review Services and approved by the [redacted for peer review] IRB (19-10020908).

For peer review only

References

1. Pingali C, Meghani M, Razzaghi H, Lamias MJ, Weintraub E, Kenigsberg TA, et al. COVID-19 Vaccination Coverage Among Insured Persons Aged ≥16 Years, by Race/Ethnicity and Other Selected Characteristics - Eight Integrated Health Care Organizations, United States, December 14, 2020-May 15, 2021. MMWR Morb Mortal Wkly Rep. 2021 Jul 16;70(28):985–90.

2. Szilagyi PG, Thomas K, Shah MD, Vizueta N, Cui Y, Vangala S, et al. Likelihood of COVID-19 vaccination by subgroups across the US: post-election trends and disparities. Hum Vaccin Immunother. 2021 Oct 3;17(10):3262–7.

3. Kriss JL, Hung M-C, Srivastav A, Black CL, Lindley MC, Lee JT, et al. COVID-19 Vaccination Coverage, by Race and Ethnicity - National Immunization Survey Adult COVID Module, United States, December 2020-November 2021. MMWR Morb Mortal Wkly Rep. 2022 Jun 10;71(23):757–63.

4. Magesh S, John D, Li WT, Li Y, Mattingly-App A, Jain S, et al. Disparities in COVID-19 Outcomes by Race, Ethnicity, and Socioeconomic Status: A Systematic-Review and Meta-analysis. JAMA Netw Open. 2021 Nov 1;4(11):e2134147.

5. Agarwal R, Dugas M, Ramaprasad J, Luo J, Li G, Gao GG. Socioeconomic privilege and political ideology are associated with racial disparity in COVID-19 vaccination. Proc Natl Acad Sci U S A [Internet]. 2021 Aug 17;118(33). Available from: http://dx.doi.org/10.1073/pnas.2107873118

6. Njoku A, Joseph M, Felix R. Changing the Narrative: Structural Barriers and Racial and Ethnic Inequities in COVID-19 Vaccination. Int J Environ Res Public Health [Internet]. 2021 Sep 20;18(18). Available from: http://dx.doi.org/10.3390/ijerph18189904

7. Bacong AM, Haro-Ramos AY. Willingness to Receive the COVID-19 Vaccine in California: Disparities by Race and Citizenship Status. J Racial Ethn Health Disparities. 2022 Nov 30;1–10.

8. Nguyen LH, Joshi AD, Drew DA, Merino J, Ma W, Lo C-H, et al. Self-reported COVID-19 vaccine hesitancy and uptake among participants from different racial and ethnic groups in the United States and United Kingdom. Nat Commun. 2022 Feb 1;13(1):636.

9. Saluja S, Nok Lam C, Wishart D, McMorris A, Cousineau MR, Kaplan CM. Disparities in COVID-19 vaccine hesitancy among Los Angeles County adults after vaccine authorization. Prev Med Rep. 2021 Dec;24:101544.

10. Piltch-Loeb R, Savoia E, Goldberg B, Hughes B, Verhey T, Kayyem J, et al. Examining the effect of information channel on COVID-19 vaccine acceptance. PLoS One. 2021 May 12;16(5):e0251095.

11. Khubchandani J, Macias Y. COVID-19 vaccination hesitancy in Hispanics and African-Americans: A review and recommendations for practice. Brain Behav Immun Health. 2021 Aug;15:100277.

For peer review only

12. Allen JD, Abuelezam NN, Rose R, Fontenot HB. Factors associated with the intention to obtain a COVID-19 vaccine among a racially/ethnically diverse sample of women in the USA. Transl Behav Med. 2021 Apr 7;11(3):785–92.

13. Thompson HS, Manning M, Mitchell J, Kim S, Harper FWK, Cresswell S, et al. Factors Associated With Racial/Ethnic Group–Based Medical Mistrust and Perspectives on COVID-19 Vaccine Trial Participation and Vaccine Uptake in the US. JAMA Netw Open. 2021 May 3;4(5):e2111629–e2111629.

14. Kricorian K, Turner K. Covid-19 vaccine reluctance in older black and hispanic adults: Cultural sensitivity and institutional trust. J Am Geriatr Soc. 2021 Apr;69(S1):S214–S214.

15. Momplaisir FM, Kuter BJ, Ghadimi F, Browne S, Nkwihoreze H, Feemster KA, et al. Racial/Ethnic Differences in COVID-19 Vaccine Hesitancy Among Health Care Workers in 2 Large Academic Hospitals. JAMA Netw Open. 2021 Aug 2;4(8):e2121931.

16. Longoria, Acosta, Urbani, Smith. A Limiting Lens: How Vaccine Misinformation Has Influenced Hispanic Conversations Online [Internet]. First Draft; 2021 Dec. Available from: https://firstdraftnews.org/wp-content/uploads/2021/12/COVID-19_VACCINE_MISINFORMATION_HISPANIC_COMMUNITIES.pdf

17. Dodson K, Mason J, Smith R. Covid-19 vaccine misinformation and narratives surrounding Black communities on social media October 13, 2021 [Internet]. First Draft News; 2021. Available from: https://firstdraftnews.org/long-form-article/covid-19-vaccine-misinformation-black-communities/

18. Momplaisir F, Haynes N, Nkwihoreze H, Nelson M, Werner RM, Jemmott J. Understanding Drivers of COVID-19 Vaccine Hesitancy Among Blacks. Clin Infect Dis [Internet]. 2021 Feb 9; Available from: http://dx.doi.org/10.1093/cid/ciab102

19. Bateman LB, Hall AG, Anderson WA, Cherrington AL, Helova A, Judd S, et al. Exploring COVID-19 vaccine hesitancy among stakeholders in African American and Latinx communities in the deep south through the lens of the Health Belief Model. Am J Health Promot. 2022 Feb;36(2):288–95.

20. Balasuriya L, Santilli A, Morone J, Ainooson J, Roy B, Njoku A, et al. COVID-19 Vaccine Acceptance and Access Among Black and Latinx Communities. JAMA Netw Open. 2021 Oct 1;4(10):e2128575.

21. Williams DR, Mohammed SA. Racism and Health I: Pathways and Scientific Evidence. Am Behav Sci [Internet]. 2013 Aug 1;57(8). Available from: http://dx.doi.org/10.1177/0002764213487340

22. Martin KJ, Stanton AL, Johnson KL. Current health care experiences, medical trust, and COVID-19 vaccination intention and uptake in Black and White Americans. Health Psychol [Internet]. 2022 Oct 13; Available from: http://dx.doi.org/10.1037/hea0001240

23. Doherty IA, Pilkington W, Brown L, Billings V, Hoffler U, Paulin L, et al. COVID-19 vaccine hesitancy in underserved communities of North Carolina. PLoS One. 2021 Nov 1;16(11):e0248542.

For peer review only

24. Liu R, Li GM. Hesitancy in the time of coronavirus: Temporal, spatial, and sociodemographic variations in COVID-19 vaccine hesitancy. SSM Popul Health. 2021 Sep;15:100896.

25. Morales DX, Paat Y-F. Hesitancy or Resistance? Differential Changes in COVID-19 Vaccination Intention Between Black and White Americans. J Racial Ethn Health Disparities. 2022 Dec 22;1–13.

26. Gaskin DJ, Dinwiddie GY, Chan KS, McCleary RR. Residential segregation and the availability of primary care physicians. Health Serv Res. 2012 Dec;47(6):2353–76.

27. Arnett MJ, Thorpe RJ Jr, Gaskin DJ, Bowie JV, LaVeist TA. Race, Medical Mistrust, and Segregation in Primary Care as Usual Source of Care: Findings from the Exploring Health Disparities in Integrated Communities Study. J Urban Health. 2016 Jun;93(3):456–67.

28. Brown EJ, Polsky D, Barbu CM, Seymour JW, Grande D. Racial Disparities In Geographic Access To Primary Care In Philadelphia. Health Aff . 2016 Aug 1;35(8):1374–81.

29. Oh NL, Biddell CB, Rhodes BE, Brewer NT. Provider communication and HPV vaccine uptake: A meta-analysis and systematic review. Prev Med. 2021 Jul;148:106554.

30. Opel DJ, Mangione-Smith R, Robinson JD, Heritage J, DeVere V, Salas HS, et al. The Influence of Provider Communication Behaviors on Parental Vaccine Acceptance and Visit Experience. Am J Public Health. 2015 Oct;105(10):1998–2004.

31. Benkert R, Cuevas A, Thompson HS, Dove-Meadows E, Knuckles D. Ubiquitous Yet Unclear: A Systematic Review of Medical Mistrust. Behav Med. 2019 Apr-Jun;45(2):86–101.

32. Siegel M, Critchfield-Jain I, Boykin M, Owens A, Muratore R, Nunn T, et al. Racial/Ethnic Disparities in State-Level COVID-19 Vaccination Rates and Their Association with Structural Racism. J Racial Ethn Health Disparities. 2022 Dec;9(6):2361–74.

33. Nguyen KH, Yankey D, Lu P-J, Kriss JL, Brewer NT, Razzaghi H, et al. Report of Health Care Provider Recommendation for COVID-19 Vaccination Among Adults, by Recipient COVID-19 Vaccination Status and Attitudes - United States, April-September 2021. MMWR Morb Mortal Wkly Rep. 2021 Dec 17;70(50):1723–30.

34. Lo C-H, Chiu L, Qian A, Khan MZ, Alhassan HA, Duval AJ, et al. Association of Primary Care Physicians Per Capita With COVID-19 Vaccination Rates Among US Counties. JAMA Netw Open. 2022 Feb 1;5(2):e2147920.

35. Stancanelli J. Conducting an Online Focus Group. The Qualitative Report; Fort Lauderdale. 2010 May;15(3):761–5.

36. Turney L, Pocknee C. Virtual focus groups: New technologies, new opportunities, new learning environments [Internet]. ascilite.org; 2004 [cited 2022 Dec 19]. Available from: https://www.ascilite.org/conferences/perth04/procs/pdf/turney.pdf

For peer review only

37. Gordon AR, Calzo JP, Eiduson R, Sharp K, Silverstein S, Lopez E, et al. Asynchronous Online Focus Groups for Health Research: Case Study and Lessons Learned. International Journal of Qualitative Methods. 2021 Jan 1;20:1609406921990489.

38. Latkin CA, Edwards C, Davey-Rothwell MA, Tobin KE. The relationship between social desirability bias and self-reports of health, substance use, and social network factors among urban substance users in Baltimore, Maryland. Addict Behav. 2017 Oct;73:133–6.

39. Shavers VL, Lynch CF, Burmeister LF. Knowledge of the Tuskegee study and its impact on the willingness to participate in medical research studies. J Natl Med Assoc. 2000 Dec;92(12):563–72.

40. Gorman JM, Gorman SE, Sandy W, Gregorian N, Scales DA. Implications of COVID-19 Vaccine Hesitancy: Results of Online Bulletin Board Interviews. Frontiers in Public Health [Internet]. 2022;9. Available from: https://www.frontiersin.org/article/10.3389/fpubh.2021.757283

41. Aw J, Seng JJB, Seah SSY, Low LL. COVID-19 Vaccine Hesitancy—A Scoping Review of Literature in High-Income Countries. Vaccines. 2021 Aug 13;9(8):900.

42. Rabaa J, Dorsaf H, Hend J, Sabrine O, Uta O, Yosra Z, et al. Perceptions of Tunisians on COVID-19 vaccines: A qualitative study on a sample of general population [Internet]. scivisionpub.com; 2021 [cited 2022 Dec 19]. Available from: http://scivisionpub.com/pdfs/perceptions-of-tunisians-on-covid19-vaccines-a-qualitative-study-on-a-sample-of-general-population-1843.pdf

43. Steinert JI, Sternberg H, Prince H, Fasolo B, Galizzi MM, Büthe T, et al. COVID-19 vaccine hesitancy in eight European countries: Prevalence, determinants, and heterogeneity. Sci Adv. 2022 Apr 29;8(17):eabm9825.

44. Güss CD, Tuason MT. Individualism and Egalitarianism Can Kill: How Cultural Values Predict Coronavirus Deaths Across the Globe. Front Psychol. 2021 Jun 22;12:620490.

45. Santos HC, Varnum MEW, Grossmann I. Global Increases in Individualism. Psychol Sci. 2017 Sep;28(9):1228–39.

46. Vraga EK, Bode L. Defining Misinformation and Understanding its Bounded Nature: Using Expertise and Evidence for Describing Misinformation. Political Communication. 2020 Jan 2;37(1):136–44.

47. Southwell BG, Brennen JSB, Paquin R, Boudewyns V, Zeng J. Defining and Measuring Scientific Misinformation. Ann Am Acad Pol Soc Sci. 2022 Mar 1;700(1):98–111.

48. Jimenez ME, Rivera-Núñez Z, Crabtree BF, Hill D, Pellerano MB, Devance D, et al. Black and Latinx Community Perspectives on COVID-19 Mitigation Behaviors, Testing, and Vaccines. JAMA Netw Open. 2021 Jul 1;4(7):e2117074.

49. Sotero M. A Conceptual Model of Historical Trauma: Implications for Public Health Practice and Research [Internet]. Journal of health disparities research and practice. 2006 [cited 2022 Dec 19]. Available from: https://papers.ssrn.com/abstract=1350062

For peer review only

50. Akinyemi AA. Historical trauma compounds experiences of racial injustice. Nat Hum Behav. 2022 Sep;6(9):1183.

51. Bajaj SS, Stanford FC. Beyond Tuskegee - vaccine distrust and everyday racism. N Engl J Med. 2021 Feb 4;384(5):e12.

52. Nguyen AL, Schwei RJ, Zhao Y-Q, Rathouz PJ, Jacobs EA. What Matters When It Comes to Trust in One's Physician: Race/Ethnicity, Sociodemographic Factors, and/or Access to and Experiences with Health Care? Health Equity. 2020 Jun 29;4(1):280–9.

53. Dong L, Bogart LM, Gandhi P, Aboagye JB, Ryan S, Serwanga R, et al. A qualitative study of COVID-19 vaccine intentions and mistrust in Black Americans: Recommendations for vaccine dissemination and uptake. PLoS One. 2022 May 3;17(5):e0268020.

For peer review only

Table 1: Characteristics of bulletin board participants

	Participants from the Black community (N=30)	Participants from the Latinx community (N=30)
**Gender**		
Male	9	14
Female	21	16
**Age**		
18-29	4	13
30-39	8	8
40-49	7	6
50-59	6	3
60-69	5	0
**Marital Status**		
Married or living with partner	5	20
Divorced or widowed	6	0
Single	19	10
**Education Level**		
Less than high school	1	3
Some college	15	13
College degree	10	10
Post-graduate	4	4
**Household Income**		
Below $35,000	9	7
$35,000-$49,999	5	3
$50-$74,999	5	11

For peer review only

$75-$99,999	8	10
$100,000+	3	0
**Flu Vaccine History**		
Usually get the vaccine	4	3
Sometimes get the vaccine	12	13
Never get the vaccine	14	14
**Religion**		
Roman Catholic	NA	19
Protestant	NA	7
None	NA	3
Mormon	NA	1
**US or Foreign-Born**		
US-born	NA	17
Foreign-born	NA	13
**Heritage Country**		
Mexico	NA	8
Peru	NA	4
Ecuador	NA	4
Dominican Republic	NA	3
Venezuela	NA	2
Puerto Rico	NA	2
Colombia	NA	2
El Salvador	NA	1
Chile	NA	1
Costa Rica	NA	1

For peer review only

Cuba	NA	1
Guatemala	NA	1

**NA: Information not requested*

For peer review only

Table 2: Topics of inquiry and discussion on the bulletin board

Day	Topics
**1**	General health and wellbeing concerns for themselves and their families<br>Sources of health and medical information and advice<br>Primary care doctors<br>Use of trusted family and home remedies<br>Personal experiences with vaccines in the past<br>Experiences with flu vaccines<br>Awareness of messaging around vaccine safety<br>Preferred sources of information<br>Use of social media for medical or health information
**2**	Things they have heard about the Covid-19 vaccines<br>How much they trust the sources<br>What, if anything, frightens them about a Covid-19 vaccine<br>Which is more frightening to them, catching Covid-19 or getting a vaccine<br>Intentions regarding a Covid-19 vaccine<br>Perceived effectiveness of the Covid-19 vaccines<br>If and how they have discussed the vaccine with their doctors<br>If and how they have discussed the vaccine with family members or friends<br>What their community and church leaders are advising them with respect to the vaccine<br>What they have heard about vaccines and vaccine safety on social media<br>How much they trust what they see on social media<br>What public health officials are saying<br>How much trust they place in public health officials
**3**	How have their communities and their families been affected by Covid-19<br>How worried are they about possibly passing Covid-19 on to at-risk members of their families<br>How important do they feel it is to eventually receive a Covid-19 vaccine<br>How important are vaccines for restoring normalcy<br>What are the best arguments they have heard in favor of vaccination<br>How do they feel about the idea of mandated vaccination<br>What information would make them feel better about getting a Covid-19 vaccine<br>Whose endorsement of vaccination would be meaningful for them<br>Responses to various pro-vaccine messages

Table 3: Example quotations of hesitancy related to safety and efficacy concerns.

Sub-Theme	Quotations
Vaccines are a "black box"	*"I don't know what they're putting in my body."*
Fears about vaccine safety	*"Vaccines lower the fear of Covid, but not the fear of long-term effects"*<br><br>*"The unknown frightens me. What happens when the vaccine interacts with medications… what happens years from now?"*<br><br>*"What frightens me is that uncertainty. No one knows what this vaccine will do to humans long term. Let alone babies that are born after."*
Conviction that the vaccine can kill you	*"I believe I would say receiving the vaccine is most frightening. I have had several people to pass away [sic] after receiving the vaccine. Prior to the vaccine these individuals were healthy and doing fine."*<br><br>*"The idea that I could die or have health complications because of the vaccine frightens me. I've mostly read this in social media."*
Concerns about scientific uncertainty	*"There are so many conflicting reports that it is difficult to know who is being honest and factual."*
Vaccines are not effective	*"With all the reports of fully vaccinated people contracting COVID a second time I'm not convinced that the vaccine offers the protection it claims."*

For peer review only

	"From what I'm hearing it would be very effective, but some people, even though they got vaccinated ended up with Covid. I'm not really sure at the moment to be honest with you"
Vaccines are insufficient	*"Currently, only 50% effective. I have seen where there's a booster shot required every six months. I've also heard doctors and CDC state that it doesn't prevent you from getting Covid, it just lessens your likelihood the virus being as bad."*
Vaccines do not prevent transmission	*"Some people that have been vaccinated have gotten the virus. I think that it causes people to lower their guard regarding social distancing and wearing masks. New more contagious strains of the virus are still popping up."*<br><br>*"They're dying from the vaccine as well. And the vaccine is not effective. They still get the virus and pass it on to other people."*

For peer review only

Table 4: Example quotations of hesitancy related to the perception that vaccines are not worth the risk.

Sub-Theme	Quotations
Vaccines are riskier than the virus	*"I have heard the Covid virus isn't too bad and I have multiple friends that have had it. So, I guess you can say I'm more worried about the vaccine than the virus itself."*<br>*"I have already had the virus and had minimal symptoms. So I guess I could say getting the vaccine is more frightening."*<br>*"Both are scary, but getting the vaccine is more frightening for me because I feel that if I got Covid I would be fine and it wouldn't affect me much."*<br>*"At this point, me getting the Covid vaccine is more frightening [than getting Covid]. I stay by myself; I only go out if need be and I am masked up."*
Vaccines are not necessary	*"I'm not frightened at all because I take great precautions. I'm more concerned about someone passing it on to me."*
Vaccines are only for the most vulnerable	*"For me personally, I don't feel like it's necessary as I am a healthy individual with no underlying health issues, and so is my husband and child."*<br>*"I think that the vaccine is important for those who are most vulnerable. If they get sick, at least it won't be as serious."*

For peer review only

Table 5: Example quotations of hesitancy related to distrust of institutions or concerns about structural/individual racism.

Sub-Theme	Quotations
Limited trust in physicians	*"I never really trust one opinion regarding health issues. I listen to what the doctors say and suggest for any illness. Next I read all information given and search the internet for reliable sources and try to gain an understanding of the situation. At that point my decision is made."*<br>*"I don't view my doctor as an expert in vaccine… Kinda like having a degree in general studies vs. a specialist… I think he's knowledgeable… but don't think the level of focus and concentration points to expert."*<br>*"I don't think anyone is an expert. You can't know everything about such a new vaccine!"*<br>*"In addition to the advice of medical professionals, I also believe firmly… in the power of being natural and how people used to cure themselves in the past… The traditional remedies work."*
Lack of trust in government	*"I have a hard time trusting anything government affiliated – because they follow government directives rather than their own expertise."*<br>*"I don't have confidence in what the government says in general. At the end of the day they are protecting themselves and I don't believe that they are concerned about those in the lowest classes. I feel like the government if [sic] capable of lying for its own benefit."*
Limited trust in public health authorities	*"I trust most of their opinions. Not all."*<br>*"I trust but may not do 100% of what they say."*
Concerns about health outcomes differing by race or ethnicity	*"I'm not convinced that being Black does affect the risks of getting Covid. I know that's what reported but I'm just not convinced that it's true….. It's not the news itself that's unbelievable, it's the source. Medical institutions have subjected Black people to abuse, exploitation and experimentation since this country's foundation. It wouldn't be the first time that Black people were misled into getting vaccines with the false hope of immunity from a deadly disease."*

For peer review only

	"I feel that my community is more at risk of catching Covid due to the history of us being ignored by health professionals and the government. Additionally, we are most likely in employment opportunities that expose us to conditions that are not ideal. I don't agree that we are experiencing more serious reactions because that implies that we are unhealthy. Unhealthy behaviors are common in America and not assigned to simply one community. If we are having serious reactions, it is most likely due to our concerns being brushed aside when we seek assistance from health care workers."<br>*"I honestly believe that the social structure of how Black people are treated in America is more so to do with the severity of the virus to this group. Less readily available access to health care, poor living situation, less money funneled into Black community…."*<br>*"I don't feel like my race affects my risk of getting [Covid] but I feel like it would affect the medical care that I received if I needed medical care while I was positive."*<br>*"I don't think it affects people differently due to ethnicity."*<br>*"I don't think that race is a factor here. Anyone can get the virus."*

For peer review only

Standards for Reporting Qualitative Research (SRQR)*

http://www.equator-network.org/reporting-guidelines/srqr/

Title and abstract

	Page/line no(s).
**Title** - Concise description of the nature and topic of the study Identifying the study as qualitative or indicating the approach (e.g., ethnography, grounded theory) or data collection methods (e.g., interview, focus group) is recommended	1
**Abstract** - Summary of key elements of the study using the abstract format of the intended publication; typically includes background, purpose, methods, results, and conclusions	2

Introduction

**Problem formulation** - Description and significance of the problem/phenomenon studied; review of relevant theory and empirical work; problem statement	4-6
**Purpose or research question** - Purpose of the study and specific objectives or questions	4-6

Methods

**Qualitative approach and research paradigm** - Qualitative approach (e.g., ethnography, grounded theory, case study, phenomenology, narrative research) and guiding theory if appropriate; identifying the research paradigm (e.g., postpositivist, constructivist/ interpretivist) is also recommended; rationale**	10-11
**Researcher characteristics and reflexivity** - Researchers' characteristics that may influence the research, including personal attributes, qualifications/experience, relationship with participants, assumptions, and/or presuppositions; potential or actual interaction between researchers' characteristics and the research questions, approach, methods, results, and/or transferability	7-9
**Context** - Setting/site and salient contextual factors; rationale**	7
**Sampling strategy** - How and why research participants, documents, or events were selected; criteria for deciding when no further sampling was necessary (e.g., sampling saturation); rationale**	7-9
**Ethical issues pertaining to human subjects** - Documentation of approval by an appropriate ethics review board and participant consent, or explanation for lack thereof; other confidentiality and data security issues	7
**Data collection methods** - Types of data collected; details of data collection procedures including (as appropriate) start and stop dates of data collection and analysis, iterative process, triangulation of sources/methods, and modification of procedures in response to evolving study findings; rationale**	7-9

For peer review only

For peer review only - http://bmjopen.bmj.com/site/about/guidelines.xhtml

**Data collection instruments and technologies** - Description of instruments (e.g., interview guides, questionnaires) and devices (e.g., audio recorders) used for data collection; if/how the instrument(s) changed over the course of the study	7-9
**Units of study** - Number and relevant characteristics of participants, documents, or events included in the study; level of participation (could be reported in results)	7-9, Table 1
**Data processing** - Methods for processing data prior to and during analysis, including transcription, data entry, data management and security, verification of data integrity, data coding, and anonymization/de-identification of excerpts	7-9
**Data analysis** - Process by which inferences, themes, etc., were identified and developed, including the researchers involved in data analysis; usually references a specific paradigm or approach; rationale**	10-11
**Techniques to enhance trustworthiness** - Techniques to enhance trustworthiness and credibility of data analysis (e.g., member checking, audit trail, triangulation); rationale**	10-11

Results/findings

**Synthesis and interpretation** - Main findings (e.g., interpretations, inferences, and themes); might include development of a theory or model, or integration with prior research or theory	11-14
**Links to empirical data** - Evidence (e.g., quotes, field notes, text excerpts, photographs) to substantiate analytic findings	Table 3

Discussion

**Integration with prior work, implications, transferability, and contribution(s) to the field -** Short summary of main findings; explanation of how findings and conclusions connect to, support, elaborate on, or challenge conclusions of earlier scholarship; discussion of scope of application/generalizability; identification of unique contribution(s) to scholarship in a discipline or field	15-19
**Limitations** - Trustworthiness and limitations of findings	19-20

Other

**Conflicts of interest** - Potential sources of influence or perceived influence on study conduct and conclusions; how these were managed	1
**Funding** - Sources of funding and other support; role of funders in data collection, interpretation, and reporting	1

*The authors created the SRQR by searching the literature to identify guidelines, reporting standards, and critical appraisal criteria for qualitative research; reviewing the reference lists of retrieved sources; and contacting experts to gain feedback. The SRQR aims to improve the transparency of all aspects of qualitative research by providing clear standards for reporting qualitative research.

For peer review only

**The rationale should briefly discuss the justification for choosing that theory, approach, method, or technique rather than other options available, the assumptions and limitations implicit in those choices, and how those choices influence study conclusions and transferability. As appropriate, the rationale for several items might be discussed together.

Reference:

O'Brien BC, Harris IB, Beckman TJ, Reed DA, Cook DA. **Standards for reporting qualitative research: a synthesis of recommendations.** *Academic Medicine*, Vol. 89, No. 9 / Sept 2014 DOI: 10.1097/ACM.0000000000000388

For peer review only

BMJ Open

"They've all endorsed it…but I'm just not there:" A qualitative exploration of Covid-19 vaccine hesitancy reported by Black and Latinx individuals

Journal:	*BMJ Open*
Manuscript ID	bmjopen-2023-072619.R1
Article Type:	Original research
Date Submitted by the Author:	30-May-2023
Complete List of Authors:	Scales, David; Weill Cornell Medical College; Critica<br>Gorman, Sara; Critica<br>Windham, Savannah; Critica<br>Sandy, William; Fluent Research<br>Gregorian, Nellie; Fluent Research<br>Hurth, Lindsay; Critica<br>Radhakrishnan, Malavika; Critica<br>Akunne, Azubuike; Critica<br>Gorman, Jack M.; Critica
**Primary Subject Heading**:	Sociology
Secondary Subject Heading:	Infectious diseases, Health services research
Keywords:	COVID-19, Anthropology < TROPICAL MEDICINE, Patient Participation, PREVENTIVE MEDICINE

SCHOLARONE™
Manuscripts

I, the Submitting Author has the right to grant and does grant on behalf of all authors of the Work (as defined in the below author licence), an exclusive licence and/or a non-exclusive licence for contributions from authors who are: i) UK Crown employees; ii) where BMJ has agreed a CC-BY licence shall apply, and/or iii) in accordance with the terms applicable for US Federal Government officers or employees acting as part of their official duties; on a worldwide, perpetual, irrevocable, royalty-free basis to BMJ Publishing Group Ltd ("BMJ") its licensees and where the relevant Journal is co-owned by BMJ to the co-owners of the Journal, to publish the Work in this journal and any other BMJ products and to exploit all rights, as set out in our licence.

The Submitting Author accepts and understands that any supply made under these terms is made by BMJ to the Submitting Author unless you are acting as an employee on behalf of your employer or a postgraduate student of an affiliated institution which is paying any applicable article publishing charge ("APC") for Open Access articles. Where the Submitting Author wishes to make the Work available on an Open Access basis (and intends to pay the relevant APC), the terms of reuse of such Open Access shall be governed by a Creative Commons licence – details of these licences and which Creative Commons licence will apply to this Work are set out in our licence referred to above.

Other than as permitted in any relevant BMJ Author's Self Archiving Policies, I confirm this Work has not been accepted for publication elsewhere, is not being considered for publication elsewhere and does not duplicate material already published. I confirm all authors consent to publication of this Work and authorise the granting of this licence.

For peer review only

"They've all endorsed it…but I'm just not there:" A qualitative exploration of COVID-19 vaccine hesitancy reported by Black and Latinx individuals

David Scales,[1,2] Sara Gorman,[2] Savannah Windham,[2] William Sandy,[3] Nellie Gregorian,[3] Lindsay Hurth,[2] Malavika Radhakrishnan,[2] Azubuike Akunne,[2] & Jack M. Gorman[2]

1. Section of Hospital Medicine, Division of General Internal Medicine, Department of Medicine, Weill Cornell Medicine, New York, NY, United States
2. Critica, Bronx, NY, United States
3. Fluent LLC, New York, NY, United States

Word Count: 5527

Corresponding Author:
David Scales
david.scales@aya.yale.edu
525 East 68th Street
New York, NY 10068

For peer review only

Abstract

Objective We sought to examine reasons for vaccine hesitancy among online communities of US-based Black and Latinx communities to understand the role of historical racism, present-day structural racism, medical mistrust, and individual concerns about vaccine safety and efficacy.

Design A qualitative study using narrative and interpretive phenomenological analysis of online bulletin board focus groups.

Setting Bulletin boards with a focus-group like setting in an online, private, chat-room-like environment

Participants Self-described vaccine hesitant participants from US-based Black (30) and Latinx (30) communities designed to reflect various axes of diversity within these respective communities in the US context.

Results Bulletin board discussions covered a range of topics related to COVID-19 vaccination. COVID-19 vaccine hesitant participants expressed fears about vaccine safety and doubts about vaccine efficacy. Elements of structural racism were cited in both groups as affecting populations but not playing a role in individual vaccine decisions. Historical racism was infrequently cited as a reason for vaccine hesitancy. Individualized fears and doubts about COVID-19 (short- and long-term) safety and efficacy dominated these bulletin board discussions. Community benefits of vaccination were not commonly raised among participants.

Conclusions While this suggests that addressing individually-focused fear and doubts are central to overcoming COVID-19 vaccine hesitancy in Black and Latinx groups, addressing the effects of present-day structural racism through a focus on community protection may also be important.

Keywords
COVID-19 Vaccines, Vaccination Hesitancy, Black, Latinx, Infodemic, Pandemics

Strengths and limitations of this study

- We employed online bulletin board groups of US-based Black and Latinx participants selected for self-described vaccine hesitancy to understand the reasons and motivations behind their stance, leveraging the online design to attract participants whose information environments consist of high proportions of online content where anti-vaccine misinformation was prominent.
- Compared to in-person focus groups, asynchronous bulletin boards allow all participants to freely express themselves with fewer concerns about speaking too much while facilitators can encourage greater participation from those who are more quiet.
- Transcripts were analyzed using various qualitative techniques, including narrative and interpretive phenomenological analysis to allow for understanding recurrent themes.
- Participants revealed that vaccine hesitancy is the result of a confluence of psychological and social considerations, but with selective focus on certain factors over others as participants weighed risks and benefits, such as high emphasis was placed on individual vaccine safety with relatively little attention to potential community-level benefits of vaccination.
- Vaccine safety and efficacy were of highest concern; however, mistrust in institutions and concerns about systemic and personal racism also featured prominently among participants' concerns.

For peer review only

Introduction

For much of the COVID-19 pandemic, rates of COVID-19 vaccination in Black and Latinx communities in the United States were lower than White communities, although the gap appears to be narrowing (1–3). This vaccination gap is especially concerning because Black and Latinx people diagnosed with COVID-19 have experienced worse clinical outcomes (4). Structural and social determinants of health like racism, socioeconomic status, access to transportation, and access to information or trusted healthcare practitioners make it difficult for people in some communities who want to be vaccinated to get vaccines, but even as vaccine uptake gaps have narrowed, a number of people continue to choose not to be vaccinated (5–7).

COVID-19 vaccine hesitancy, defined as “indecision around accepting a vaccination” (8), among Black and Latinx people has been found in survey studies to be higher than among White people (9–11). In their review of 13 studies of racial and ethnic disparities in COVID-19 vaccination status, Khubchandani and Macias[9] found an overall pooled rate of vaccine hesitancy of 26.3%, but a higher rate among Hispanic (30.2%) and African American (40.6%) study participants (12). Even among healthcare workers, COVID-19 vaccine hesitancy was found to be higher among Black and Latinx people compared with White people (13).

The literature on COVID-19 vaccine hesitancy among Black and Latinx people highlights three influential factors affecting vaccine decisions in these populations: individual fears and concerns about vaccine adverse side effects and efficacy, historical mistreatment in medical/scientific contexts (e.g., the US Public Health Service syphilis study at Tuskegee), present-day experience with mistreatment by the healthcare system, (including structural racism), the latter two of which are linked to mistrust in healthcare providers and the healthcare system. The literature on each of these topics is vast and a full review is beyond the scope of our study. However, there are some key studies worth highlighting that informed and motivated the research presented here.

Survey data have shown that concerns about vaccine safety and efficacy are associated with higher rates of vaccine hesitancy among Black and Latinx people (14,15). In a survey using U.S. census data, Black people were more likely than white people to develop COVID-19 vaccine hesitancy because of lack of confidence in the safety and efficacy of vaccines and because of a tendency to watch evolving information and wait before considering vaccination, though this group saw the greatest percentage drop in hesitancy over time (16). Longoria and colleagues found fears about COVID-19 vaccine safety were commonly circulated online among Latinx people, as well as narratives about alleged “alternative treatments” (17). Recent work by Morales and Paat (2022) provides additional evidence of a “watch and wait” approach among Black Americans, noting how rates of vaccine hesitancy and refusal in this community declined over time while it remained stable in White communities (18), consistent with more people seeking the vaccine as more time passes demonstrating the safety and efficacy of the vaccine.

Early in the vaccine roll-out, there was significant concern among public health professionals that well-known narratives of historical racism among marginalized communities around, for example, Henrietta Lacks (19) or the syphilis study at Tuskegee (20), would engender vaccine hesitancy due to a “legacy of distrust” in medical research (21). However, much empirical research highlighted that past narratives around Henrietta Lacks or the unethical syphilis experiments performed in Tuskegee relate to and shape contemporary lived experiences, perceptions of racism in medicine, and mistrust in the healthcare system in general and COVID-19 vaccines specifically (22). Using survey data, Martin, Stanton and Johnson (23) found that current mistreatment by the healthcare system, rather than historical mistreatment as exemplified by the Tuskegee experiments, was associated with COVID-19 vaccine hesitancy among Black Americans. Another small study among Black undergraduates described these historical examples of racist and unethical practices as “backdrops” that informed their contemporary perceptions of how Black people continue to be discounted (24).

Additionally, present day manifestations of structural racism with historical antecedents, for example, the downstream impact of redlining, perpetuate healthcare access and socioeconomic disparities (25,26) that subsequently are likely to influence vaccine-related decisions. For various geographic and socioeconomic reasons, Black people are less likely to have access to a primary care physician (27–29) and more likely to use emergency care, a relationship partly mediated by mistrust in the healthcare system (28). Therefore, while primary care physicians are often cited across different racial and ethnic groups as the most trusted person when it comes to vaccine decision-making (30,31), structural inequalities in healthcare access means that many marginalized communities lack access to these heavily trusted sources (32,33). Some population-based studies support the link between access to primary care and those provider recommending vaccines to higher rates of COVID-19 vaccine uptake (34,35).

As alluded to above, the lived experience of historical racism, present day racism, including structural manifestations of it, and medical mistrust are intertwined. For example, one qualitative study linked historical mistrust to the way Black people in the Deep South have been treated by the healthcare system as a factor contributing to vaccine hesitancy (36). Another focus group study with Black and Latinx community members also identified "pervasive mistreatment" as a basis for vaccine hesitancy in those communities (37), suggesting that it is difficult to separate perceptions of medical racism from institutional mistrust in healthcare. Similarly, an analysis of online posts found that mistrust of vaccines and the motivations of official institutions (i.e. institutional mistrust) were commonly expressed in online platforms viewed by the Black community (38). Similar concerns were heard in focus groups of Black salon and barbershop owners (39) and in a study focusing on older Black and Hispanic adults (40). In addition to these fears, Bateman and colleagues conducted virtual focus groups and identified mistrust of the COVID-19 vaccine development process among Black and Latinx participants from the Deep South (36). Similarly, while conceptually separated here, concerns

about vaccine safety are also likely intermixed with institutional mistrust and experiences with racism since safety is assessed by government agencies. For example, a survey of people in underserved communities in North Carolina identified safety concerns and government mistrust as the most important factors for vaccine hesitancy among Black and Latinx respondents (41).

The purpose of this study was to probe more deeply into these factors to get a better understanding of the complexities involved in lower COVID-19 vaccine uptake among Black and Latinx people. To do this, we conducted two separate online bulletin boards, one with participants from each community who self-identified as vaccine hesitant, to probe the factors behind their vaccine decisions. We were interested in understanding what motivates people in the Black and Latinx communities to be COVID-19 vaccine hesitant. Originally, we intended this as two separate inquiries, one involving Black and the other involving Latinx participants and therefore the designs and recruitment strategies of the online bulletin boards differed. However, we observed remarkably similar responses from participants in the two groups and therefore decided to combine them into a single report.

These bulletin boards were conducted within the first few months after COVID-19 vaccines were made available and reflect attitudes at that time. Gaps in COVID-19 vaccination rates among racial and ethnic groups have since narrowed and attitudes about vaccines may have shifted. However, the results of these bulletin boards remain important for two reasons: first, because they provide insight into important drivers of vaccine hesitancy in Black and Latinx communities; and second, because they may help inform strategies to support future vaccine demand as new healthcare challenges inevitably arise.

Methods

We conducted two bulletin boards from July 13 to 22, 2021, following the COVID-19 vaccine rollout in the U.S. Informed consent via an online form was obtained from each participant prior to the start of the study, and they were assured that participation was voluntary.

For peer review only - http://bmjopen.bmj.com/site/about/guidelines.xhtml

Participants were told that they could end their participation at any time and were free to leave any questions unanswered. Subjects were paid $120 for their participation. This research was deemed exempt from IRB review by Ethical and Independent Review Services and approved by the Weill Cornell IRB. Due to privacy concerns, data are not currently publicly available, but de-identitified data can be obtained by researchers on a case-by-case basis by contacting the authors.

A bulletin board is an asynchronous online discussion involving greater numbers of individuals than typical focus groups and taking place over an extended period (42–44). Participants log into a password-protected site run by an external third party (QualBoard, since acquired by Sago) that creates such dedicated platforms to answer questions that are posted and monitored by a moderator. The moderator can also follow up on responses for clarification or elaboration. The bulletin board is a flexible research tool that allows the moderator to post questions and probe any individual participant following their entry. The respondents can take as much time to respond as they need. Individual responses are initially uninfluenced by the group, as participants do not see other responses to any given question until they have posted their own response. This method helps to minimize the social desirability bias (45) that may influence participants after exposure to another's responses.

Data collection. Participants in the bulletin board with Black participants were recruited from a panel of people who have previously agreed to participate in online surveys. They were contacted by email with an invitation to participate. If interested, they were asked to respond to an online screener that assessed their level of vaccine hesitancy. People who had already been vaccinated, intended to be vaccinated soon, or who adamantly opposed COVID-19 vaccination were excluded. For recruiting purposes, we defined vaccine hesitancy as adults who were not categorically opposed to vaccines, but were undecided as to whether it was safe to receive the new COVID-19 vaccines. We then screened for individuals that met these criteria. If qualified,

For peer review only

they provided their contact information and were given instructions for logging into the bulletin board. Please see Table 1 for demgraphics of the participants.

The process for recruiting participants from Latinx communities differed from that for Black participants. We posted invitations in Spanish on various Facebook pages created for Latinx sub-populations, such as groups for communities from Peru, Colombia, Mexico and the Dominican Republic. If interested, they were asked to complete the online screening questionnaire in Spanish, to determine if they met the criteria for participation, which were the same as for the participants in the Black groups as described above. If qualified, they provided their contact information, and were supplied with instructions for logging into the bulletin board. This project was designed to inform subsequent interventions to address vaccine hesitancy. Therefore, the demographics of each group were chosen to approximately match those of the groups in which interventions would take place in a later study based on observational assessment of such spaces by our interventionists (for more info on these interventions, see (46,47). Similarly, our funding for this project came from a source exclusively prioritizing health in the United States, we focused on finding participants that were relatively representative of these racial and ethnic groups living in the US according to US Census American Community Survey 5-year estimates.

When participants logged into the bulletin board, they were presented with an introduction from the moderator, a review of the process, and a reminder that they were not obliged to answer any question. They were reassured that the research was anonymous and their identities, including contact information, would not be shared. The moderators of the bulletin boards introduced themselves at the outset and posted their photographs so that the respondents could see them. Participants were allowed to post photographs of themselves to the group, though this was not required.

Participants were then presented with the first of a series of questions. Only after a participant entered their response to a question were they able to see how other participants

responded to that same question. At this point, they were free to respond to what other participants had said. After responding to all of the questions posted for that day, they were reminded to check back periodically to respond to possible follow-up questions posted by the moderator. This process continued over three days, with a different set of questions posted each day.

Bulletin board questions were designed for flexible, open-ended inquiry. The research did not seek to confirm any hypotheses but rather to explore the range of perceptions and attitudes that exist in the vaccine hesitant population and to identify important influencers of those perceptions and attitudes, including trusted sources of information, media outlets, social networks, community leaders, health professionals, etc. Examples of the various topics of inquiry and discussion can be found in Table 2 and the facilitators guide included in supplementary material. We also asked participants' perspectives on influenza vaccines, but only data from questions about COVID-19 vaccines are included here for parsimony.

Data quality control. The study employed purposive sampling with screening to ensure that respondents reflected the target population in terms of attitudinal, behavioral, and demographic characteristics. The sample was highly diverse with respect to age, geography, socio-economic status, and in the case of the Latinx sample, with respect to both level of acculturation to the U.S. and national heritage (see Table 1).

The bulletin boards were conducted by trained moderators, each with 20+ years of qualitative research experience. The Latinx bulletin boards were conducted in Spanish by a Latinx moderator; the Black bulletin boards were conducted in English by a Black moderator. The Spanish-language discussion among Latinx respondents was translated into English by an automated translation program provided by the online platform. This was done for the benefit of those observing the discussion who were not Spanish speakers. The automated translation was

For peer review only - http://bmjopen.bmj.com/site/about/guidelines.xhtml

not used, however, for the purposes of reporting due to some translation errors. Transcripts included in the report were translated by professional Spanish-speaking moderators.

Methods of analysis. A combination of methods was employed in the analysis of the content generated by these bulletin boards, including interpretive phenomenological analysis (IPA), narrative analysis, and, secondarily, qualitative content analysis. Taking a reflexive, phenomenological constructivist approach to understand participant viewpoints, these methods enabled us to explore how respondents narrate and make sense of their prior experiences with vaccines, with medical professionals, and with various sources of medical and health-related information. They also enabled us to observe how participants rationalize their hesitancy with respect to COVID-19 vaccination, and to identify a range of social, emotional, and perceptual barriers to vaccination. Analysis enabled us to identify the range of opinions exhibited, opinions that are universally shared and those that are more idiosyncratic and portray how different perceptions tend to be clustered or coupled.

Initial coding was done manually by WS. Transcripts were read through once in their entirety by prior to coding. On a second readthrough, relevant text was highlighted, codes were inductively drawn out, and labeled in text margins. Codes were then aggregated and organized into themes which were discussed in meetings with co-authors discussed in more detail below. Themes were then arranged with key quotations pulled out as illustrative examples.

Data credibility was assessed through discursive triangulation. Initial coding was followed by a process of layered discussions. Specifically, that meant multiple meetings between the primary data analyst and a supervisor to process codes and themes, followed by further coding and thematic discussions and revisions with the first and senior author. After consensus on themes had been reached by those four authors, subsequent review and discussion then took place with the remaining co-authors.

For peer review only

Interpretive phenomenological analysis (IPA). Data collection was not designed to test hypotheses or preconceptions, nor was data analysis. The intent was to use the data gathered to better understand the experiential world of the respondents, how they understand the phenomenon of the ongoing pandemic, and how they rationalize their decision to refrain from vaccination (48). Through this bottom-up analysis, we sought commonalities and patterns in experiences and shared forms of reasoning to inform a richer understanding of vaccine hesitancy. In addition, the analysis included any consistent variations in participants' responses that corresponded with major demographic variables such as gender and age. For the descriptive analysis, we identified and cataloged the fullest possible range of opinions around vaccine hesitancy, including commonly cited sources of information, facts, anecdotes, and trusted sources of information, regarding the pandemic and COVID-19 vaccines.

Narrative analysis. In addition to identifying and cataloging the range of opinions and perceptions articulated by participants, the analysis focused on identifying the ways in which information has been woven into narratives. This analysis focused on participants' descriptions of their experiences during the pandemic, their methods for searching for and processing relevant information, and the stories they tell themselves about the need or lack of need for a vaccine. In addition, we analyzed the trajectory of each individual participant's experience with vaccines, looking to identify key moments when their attitudes reportedly changed. This analysis also sought to identify pre-existing narratives and how those intersect with participants' narratives about the pandemic, such as mistreatment of marginalized populations by the healthcare system and lack of trust in the government. This analysis also attempted to gauge the extent to which participants' narratives are fixed, are still being formed, or remain open to revision.

For peer review only

As an additional check on our data and to ensure we met the objective of the study, we examined certain topics of high concern (historical racism, present-day structural racism, and medical mistrust) using a directed approach in secondary content analysis (49).

Patient and Public Involvement

None.

Results

We conducted one bulletin board with 30 people from the Black community and one with 30 people from the Latinx community. Characteristics of the participants can be found in Table 1. The themes obtained from the bulletin boards about COVID-19 vaccines in both the Black and Latinx groups were remarkably similar and therefore we combined them in this section. The analysis suggests several interrelated barriers to COVID-19 vaccination are at work in both Black and Latinx communities, strongly influencing vaccine behaviors in these populations. Five main themes and several sub-themes emerged. Illustrative quotations can be found in Tables 3, 4, and 5:

1. **Safety concerns (Table 3)**

- Vaccine unknowns. Vaccines are a “black box.” Some participants perceived vaccine ingredients to be elusive or intentionally obscured with mysterious ingredients.
- Fears about COVID-19 vaccine safety. Participants expressed many fears and doubts regarding both the short- and long-term safety of the vaccines; even those who express high trust in doctors and science and low trust in social media still say stories of vaccine-induced illness make them highly uncertain.
- Conviction that the COVID-19 vaccine can kill you. Some participants believe that the vaccine is directly responsible for deaths.

For peer review only

- Concerns about scientific uncertainty. Public scientific debates about vaccine safety and adverse side effects instill and perpetuate doubts by creating the appearance of scientific uncertainty even among those who normally trust medical professionals. Many seem to almost throw up their hands and say, “I can’t decide what’s true and what’s not, so best to do nothing,” or to wait for more conclusive information.

2. Skepticism about vaccine efficacy (Table 3)

- COVID-19 vaccines are not effective. Several stories about new variants, breakthrough infections, and surging cases suggested a belief that the vaccine would not be effective in protecting them.
- COVID-19 vaccines are insufficient. Even with an effective vaccine, mass vaccination is not enough to return life to normal and that COVID-19 is here to stay, implying that the vaccine’s benefits may be exaggerated.
- COVID-19 vaccines do not prevent transmission. Although vaccines reduce the risk of transmission, news that people can still pass COVID-19 on to others even after being vaccinated is conflated with a narrative that vaccines do not work as intended, thus undermining the argument for getting it to protect others.

3. Risk/benefit calculations were not perceived to favor vaccines (Table 4)

- Vaccines are riskier than the virus. Participants frequently assigned greater risk to the vaccine than to the virus itself and noted that there are other ways to prevent infection (like masking), so on balance the vaccines are felt to be unnecessary.
- COVID-19 vaccines are not necessary. Participants in both groups often believed they were not at risk of dying from COVID-19; they believed they could contract the virus and

For peer review only

recover from it. They also believed that any illness would be mild, underscoring a lack of urgency to be vaccinated.

- COVID-19 vaccines are only for the most vulnerable. Vaccines are for the most vulnerable, such as older people and immunocompromised people, not the young and healthy, or those being careful and taking other precautions.

4. Limited trust in institutions (Table 5)

- Limited trust in physicians. Many say that they trust their primary care doctors the most when it comes to their health, but that trust does not always extend to advice about the COVID-19 vaccines; they do not necessarily see their doctors as experts in this regard. For instance, some seem to say, "at this point, no one can claim to be an expert on these vaccines. So, no one can truly tell me what is best."
- Lack of trust in government. There is a lack of trust in government in general and especially in government spokespersons, undermining their authority as credible messengers. Many tune them out or do not lend them credence, even those who otherwise trust their doctors and medical professionals. Some people suggest that the very fact that the government so badly wants them to get a vaccine makes them not want to get it.
- Limited trust in public health authorities. While some of the vaccine hesitant respondents expressed very high regard for medical professionals and for public health authorities in general, they were more critical of public agency-relayed information about COVID-19 vaccines. Some argued that public health authorities only say what they are told to say by the administration. Some participants mistrust the CDC, largely because they viewed the agency as frequently changing its advice and guidelines.

For peer review only

5. How health outcomes differ by race and/or ethnicity (Table 5)

Participants in both groups perceived structural racism as factors that influence a group's risk of infection and the likelihood of having access to vaccines. However, the participants did not cite structural issues as influencing either their own personal risk of infection or their decision to be vaccinated. Two people mentioned the Tuskegee experiments (50) as indicative of abuses against Black people by the healthcare system and a reason to be wary of healthcare system programs, including COVID-19 vaccines. The potential benefits of vaccination, such as protecting vulnerable communities, were not raised as a motivation for vaccination.

Other observations. Although themes and sub-themes about vaccine hesitancy were quite similar between the Black and Latinx groups in this study, there was one notable difference in where participants noted obtaining health information. Black participants were more likely to emphasize obtaining information about COVID-19 vaccines from the internet, despite having what appears to be strong relationships with medical providers. Latinx participants also had strong relationships with and trust in medical providers and seemed to make less use of the internet for health information. Both groups rely heavily on trusted friends and relatives for health information. Even with that support, however, moving to vaccine acceptance for some people can be very difficult and take more time than for others. As one Black participant noted: "*I love and trust my family; I love and trust my pastor. And they all made their position known. And I know none of them do things haphazardly… [but] the jury is still out for me… I'm just straddling the fence, and it's just a personal thing with me… They've all endorsed it, my pastor endorsed it…but I'm just not there, I'm not."*

Aside from the difference noted above, we did not observe distinct themes specific to any particular sub-group (e.g. by country of origin). Additionally, it should be noted that

For peer review only

participants did not endorse conspiracy theories or unsubstantiated notions about vaccines (e.g., that they contain microchips) that have been voiced in anti-vaccine channels.

Discussion

The results of this research suggest that interrelated barriers to vaccination are at work in communities of color and strongly influence COVID-19 vaccine behaviors in these communities. Two main sets of concerns emerged from in these bulletin boards: that the vaccines are unsafe and that they are insufficiently effective. These concerns are remarkably similar to those observed in an earlier bulletin board study that involved a group of participants that had a majority of white people (51). Indeed, these may be ubiquitous influences on vaccine hesitancy across racial, ethnic, and national groups (52–54).

Several participants seemed eager to make clear they felt race and ethnicity were factors in community viral infection susceptibility because of the history of structural racism in healthcare and medicine. Lack of access to healthcare and to vaccination sites has been found to be a factor in limiting vaccination among Black and Latinx people (37). The history of racism and medical experimentation on people of color in the United States was cited as among the reasons for vaccine skepticism among Black participants in one recent study (55). However, as in the survey study of Martin, Stanton, and Johnson (23), participants in our study did not frequently express a conviction that historical racism was a factor in their *personal* decisions about vaccination. Two of the 30 participants in our Black participant group directly named the Tuskegee experiments, a hallmark of unethical, racist scientific and healthcare practices in the U.S. Thus, although participants in both groups often cited examples of structural racism in general, they were more likely to express individual feelings of fear and skepticism about the vaccines as the main factors in making them hesitant to be vaccinated.

Historical traumas like the experiments that took place in Tuskegee may still have an effect on people's attitudes and decision making even if not explicit (56,57). Current experiences

For peer review only

with racism such as health outcome disparities may be as or even more important in shaping ways that people of color make decisions about healthcare issues like vaccination (58). It is possible that the way we framed questions in these bulletin boards influenced participants toward speaking more about their individual concerns as the main factors in COVID-19 vaccine decision making and away from broader discussions about the impact of historical and present racism on those decisions. It is also possible that for these participants at least, while recognizing that structural factors like crowded work conditions and lack of healthcare access make communities of color more likely to acquire COVID-19 and to have more negative outcomes, individual fears and skepticism about the COVID-19 vaccines were indeed the most pressing concerns that influenced vaccine hesitancy.

Although participants expressed trust in their own personal healthcare providers, they exhibitied a general lack of trust in agencies and institutions that are charged with the responsibility of informing and reassuring the public about vaccine safety and efficacy. This is not a unique finding. Previous studies have found people in Black and Latinx communities have low levels of trust in the healthcare system (27), and racial differences in healthcare access has been noted as a contributing factor (28).

This study also raises key questions about the information environments participants were immersed in. In his Special Advisory on misinformation in 2021, the US Surgeon General discussed the need to build a healthy "information environment" (59). While a worthy endeavor, there are currently no standard ways to measure whether individual or community is immersed in a healthy information environment, defined as where people and communities are immersed in high-quality information of public health importance and enveloped by a communication context that underscores the trustworthiness and importance of that quality (60). It is important to note that, at the time the bulletin boards were being done, media coverage of about the vaccines contained a mixture of concerning and reassuring information, first about breakthrough infections, viral variants, and prominent reporting on rare vaccine adverse side effects, and

For peer review only

second, describing a public health and scientific consensus that that the vaccines were safe and they were highly effective at preventing morbidity and mortality due to COVID-19.

Yet quotations from participants generally reflected more of the concerns than reassuring information, providing some insight into what their information environment may consist of. Specifically, while it was clear that participants saw and heard abundant information about vaccines, much of that information did not appear to accurately present information about both individual risks and collective benefits. Concerningly, suggests that the concerns that were circulating in these communities then met most definitions of scientific misinformation (61,62). An alternative explanation is that despite the presentation of high-quality information, misinformation or worrisome information about the vaccines could have been more effective at shaping vaccine-related feelings and decisions. In either case, the implications are concerning. Therefore, it seems unlikely that merely supplying more facts about vaccine safety and efficacy will be sufficient on its own to sufficiently modify that information environment and change participants' views on obtaining vaccines.

Some key points that public health professionals may consider when contemplating how to encourage vaccine uptake in their work with marginalized communities include:

- Leveraging trusted sources to challenge narratives of safety and inefficacy by emphasizing personal and communal benefits over risk (e.g. to protect one from new strains or to protect loved ones, for example). While previous studies of HPV vaccines observed Black people did not leverage family and friends for information (63,64), our data suggest this may differ by vaccine. Given how both Black and Latinx participants noted obtaining much of their health information from family and friends, encouraging those who have been vaccinated to reassure unvaccinated family members and friends about safety may be an efficient way to disrupt these narratives in these priority communities. This approach would be consistent with others' recommendations to

strengthen pro-vaccine messages to leverage non-medical, in-group spokespeople to share community benefits of vaccines with Black communities (65).

- Explicitly and more emphatically framing both risks of COVID-19 and benefits of vaccination in community-level terms. It is unclear why participants often offered narratives of racial discrimination at structural and community levels yet narratives about information gathering, the negative effects of COVID-19 and benefits from vaccination were not conceptualized in a similar light. This suggests both the success of current messaging on issues of structural racism, and the insufficiency of public health messaging to penetrate dominant media narratives framing aspects of COVID-19 in primarily individualistic terms (66). In this way, the data presented here reflect concerns stated elsewhere about the “individualization of pandemic control” (67,68).

- As many participants noted institutional mistrust in government and the health system, it it will be essential for public health professionals to leverage partnerships to effectively reach marginalized community members with trusted messengers. This includes improving access to vaccines for people in traditionally underserved communities as access issues can foment mistrust and suspicion of the healthcare system and efforts to ameliorate them may make some people more likely to accept vaccines (69). Access must also be coupled with sufficient training to primary care clinicians to build trust with Black and Latinx communities. As many participants noted that they did not consider their physician sufficiently expert to trust their opinion on vaccines, counteracting misinformed ideas held about vaccines by patients will only be successful if there is sufficient trust in the relative expertise of those care providers.

Limitations

For peer review only

This study has several limitations, including inherent self-selection bias in the sample of participants. There is also inevitable bias toward the views of those comfortable sharing their opinions in a group discussion with others in a digital setting where social desirability bias may make some participants reticent to share what may be perceived as outlandish opinions. This may have been a factor in the fact that subjects did not, for the most part, mention historically racist events and the United States' racialized history does not rule out that these are important factors for vaccine hesitancy. We did not ask specific questions about these issues. Participants' reports of mistrust of public health authorities and the government represent the result of both historical racism and personal experiences of racism. Thus, while we can report our observation that for the most part neither people in our Black nor Latinx groups volunteered racism as affecting their own vaccine decisions, deeper probing might have elicited that as an important factor. Indeed, Dong and colleagues conducted semi-structured interviews with 24 Black Americans and reported that, "systemic racism was discussed as the root cause of the different types of mistrust" (70).

In summary, bulletin boards with COVID-19 vaccine-hesitant people from the Black and Latinx communities revealed that the major factors influencing vaccine hesitancy involve fears of lack of safety and efficacy of the vaccines. There is a misperception that not being vaccinated is a social norm because of media emphasis on unvaccinated people. These attitudes are reinforced by a perception of lack of consensus about the vaccines among experts, mistrust of government officials and institutions, and belief that other measures are sufficient to prevent acquisition and spread of COVID-19**.** Future research will focus on strategies to improve vaccine acceptance that do not rely only on providing facts but account also for the anxieties and fears that motivate vaccine hesitancy.

For peer review only

Contributorship Statement

DS and JMG conceived of the study, wrote the protocol, obtained funding and engaged WS and NG to carry out the research and conduct preliminary data analysis. DS, SG, JMG, WS and NG were responsible for inclusion/exclusion criteria of both bulletin board groups. WS and NG were responsible for bulletin board design, and recruitment of participants. WG undertook initial interpretive phenomenological and narrative analysis. SG, SW, LH, MR, AA contributed to further data review and interpretation with DS and JMG. All authors read and critically evaluated multiple drafts of the manuscript before providing final approval of the version to be published.

Competing Interests

The authors state they have no competing interest to declare.

Funding

This work was supported by the Robert Wood Johnson Foundation (grant numbers 76935 and 78084) and Weill Cornell Medicine's JumpStart program (Grant number N/A). The opinions expressed in this piece do not necessarily reflect those of either the Robert Wood Johnson Foundation or Weill Cornell Medicine.

Data Sharing Statement

Due to privacy concerns, data are not currently publicly available, but de-identitified data can be obtained by researchers on a case-by-case basis by contacting the authors. Patients were not involved in the design of the study.

Ethics Approval Statement

This research was deemed exempt from IRB review by Ethical and Independent Review Services and approved by the Weill Cornell IRB (19-10020908).

Acknowledgments

This work was supported by the Robert Wood Johnson Foundation (grant numbers 76935 and 78084) and Weill Cornell Medicine's JumpStart program (grant number N/A). The opinions expressed in this piece do not necessarily reflect those of either the Robert Wood Johnson Foundation or Weill Cornell Medicine.

Conflicts of Interest

The authors state that they have no conflicts of interest to declare.

For peer review only

References

1. Pingali C, Meghani M, Razzaghi H, Lamias MJ, Weintraub E, Kenigsberg TA, et al. COVID-19 Vaccination Coverage Among Insured Persons Aged ≥16 Years, by Race/Ethnicity and Other Selected Characteristics - Eight Integrated Health Care Organizations, United States, December 14, 2020-May 15, 2021. MMWR Morb Mortal Wkly Rep. 2021 Jul 16;70(28):985–90.

2. Szilagyi PG, Thomas K, Shah MD, Vizueta N, Cui Y, Vangala S, et al. Likelihood of COVID-19 vaccination by subgroups across the US: post-election trends and disparities. Hum Vaccin Immunother. 2021 Oct 3;17(10):3262–7.

3. Kriss JL, Hung M-C, Srivastav A, Black CL, Lindley MC, Lee JT, et al. COVID-19 Vaccination Coverage, by Race and Ethnicity - National Immunization Survey Adult COVID Module, United States, December 2020-November 2021. MMWR Morb Mortal Wkly Rep. 2022 Jun 10;71(23):757–63.

4. Magesh S, John D, Li WT, Li Y, Mattingly-App A, Jain S, et al. Disparities in COVID-19 Outcomes by Race, Ethnicity, and Socioeconomic Status: A Systematic-Review and Meta-analysis. JAMA Netw Open. 2021 Nov 1;4(11):e2134147.

5. Agarwal R, Dugas M, Ramaprasad J, Luo J, Li G, Gao GG. Socioeconomic privilege and political ideology are associated with racial disparity in COVID-19 vaccination. Proc Natl Acad Sci U S A [Internet]. 2021 Aug 17;118(33). Available from: http://dx.doi.org/10.1073/pnas.2107873118

6. Njoku A, Joseph M, Felix R. Changing the Narrative: Structural Barriers and Racial and Ethnic Inequities in COVID-19 Vaccination. Int J Environ Res Public Health [Internet]. 2021 Sep 20;18(18). Available from: http://dx.doi.org/10.3390/ijerph18189904

7. Bacong AM, Haro-Ramos AY. Willingness to Receive the COVID-19 Vaccine in California: Disparities by Race and Citizenship Status. J Racial Ethn Health Disparities. 2022 Nov 30;1–10.

8. Larson HJ, Jarrett C, Schulz WS, Chaudhuri M, Zhou Y, Dube E, et al. Measuring vaccine hesitancy: The development of a survey tool. Vaccine. 2015 Aug 14;33(34):4165–75.

9. Nguyen LH, Joshi AD, Drew DA, Merino J, Ma W, Lo C-H, et al. Self-reported COVID-19 vaccine hesitancy and uptake among participants from different racial and ethnic groups in the United States and United Kingdom. Nat Commun. 2022 Feb 1;13(1):636.

10. Saluja S, Nok Lam C, Wishart D, McMorris A, Cousineau MR, Kaplan CM. Disparities in COVID-19 vaccine hesitancy among Los Angeles County adults after vaccine authorization. Prev Med Rep. 2021 Dec;24:101544.

11. Piltch-Loeb R, Savoia E, Goldberg B, Hughes B, Verhey T, Kayyem J, et al. Examining the effect of information channel on COVID-19 vaccine acceptance. PLoS One. 2021 May 12;16(5):e0251095.

For peer review only - http://bmjopen.bmj.com/site/about/guidelines.xhtml

12. Khubchandani J, Macias Y. COVID-19 vaccination hesitancy in Hispanics and African-Americans: A review and recommendations for practice. Brain Behav Immun Health. 2021 Aug;15:100277.

13. Momplaisir FM, Kuter BJ, Ghadimi F, Browne S, Nkwihoreze H, Feemster KA, et al. Racial/Ethnic Differences in COVID-19 Vaccine Hesitancy Among Health Care Workers in 2 Large Academic Hospitals. JAMA Netw Open. 2021 Aug 2;4(8):e2121931.

14. Allen JD, Abuelezam NN, Rose R, Fontenot HB. Factors associated with the intention to obtain a COVID-19 vaccine among a racially/ethnically diverse sample of women in the USA. Transl Behav Med. 2021 Apr 7;11(3):785–92.

15. Thompson HS, Manning M, Mitchell J, Kim S, Harper FWK, Cresswell S, et al. Factors Associated With Racial/Ethnic Group–Based Medical Mistrust and Perspectives on COVID-19 Vaccine Trial Participation and Vaccine Uptake in the US. JAMA Netw Open. 2021 May 3;4(5):e2111629–e2111629.

16. Liu R, Li GM. Hesitancy in the time of coronavirus: Temporal, spatial, and sociodemographic variations in COVID-19 vaccine hesitancy. SSM Popul Health. 2021 Sep;15:100896.

17. Longoria, Acosta, Urbani, Smith. A Limiting Lens: How Vaccine Misinformation Has Influenced Hispanic Conversations Online [Internet]. First Draft; 2021 Dec. Available from: https://firstdraftnews.org/wp-content/uploads/2021/12/COVID-19_VACCINE_MISINFORMATION_HISPANIC_COMMUNITIES.pdf

18. Morales DX, Paat Y-F. Hesitancy or Resistance? Differential Changes in COVID-19 Vaccination Intention Between Black and White Americans. J Racial Ethn Health Disparities. 2022 Dec 22;1–13.

19. Skloot R. The Immortal Life of Henrietta Lacks. Crown Publishing Group, a division of Random House, Incorporated; 2017. 381 p.

20. Reverby SM. Examining Tuskegee: The Infamous Syphilis Study and Its Legacy. Univ of North Carolina Press; 2009. 416 p.

21. Gamble VN. A legacy of distrust: African Americans and medical research. Am J Prev Med. 1993 Nov-Dec;9(6 Suppl):35–8.

22. Williams DR, Mohammed SA. Racism and Health I: Pathways and Scientific Evidence. Am Behav Sci [Internet]. 2013 Aug 1;57(8). Available from: http://dx.doi.org/10.1177/0002764213487340

23. Martin KJ, Stanton AL, Johnson KL. Current health care experiences, medical trust, and COVID-19 vaccination intention and uptake in Black and White Americans. Health Psychol [Internet]. 2022 Oct 13; Available from: http://dx.doi.org/10.1037/hea0001240

24. Soboti JM. “If You are Going to Step on This Campus, You Have to Get Vaccinated”: A Qualitative Understanding of COVID-19 Vaccine Hesitancy Among Black Emerging Adults Returning to College Campuses. Emerging Adulthood. 2023 Jun 1;11(3):735–47.

For peer review only

25. King CJ, Buckley BO, Maheshwari R, Griffith DM. Race, place, and structural racism: A review of health and history in Washington, D.c. Health Aff . 2022 Feb;41(2):273–80.

26. Egede LE, Walker RJ, Campbell JA, Linde S, Hawks LC, Burgess KM. Modern Day Consequences of Historic Redlining: Finding a Path Forward. J Gen Intern Med. 2023 May;38(6):1534–7.

27. Gaskin DJ, Dinwiddie GY, Chan KS, McCleary RR. Residential segregation and the availability of primary care physicians. Health Serv Res. 2012 Dec;47(6):2353–76.

28. Arnett MJ, Thorpe RJ Jr, Gaskin DJ, Bowie JV, LaVeist TA. Race, Medical Mistrust, and Segregation in Primary Care as Usual Source of Care: Findings from the Exploring Health Disparities in Integrated Communities Study. J Urban Health. 2016 Jun;93(3):456–67.

29. Brown EJ, Polsky D, Barbu CM, Seymour JW, Grande D. Racial Disparities In Geographic Access To Primary Care In Philadelphia. Health Aff . 2016 Aug 1;35(8):1374–81.

30. Oh NL, Biddell CB, Rhodes BE, Brewer NT. Provider communication and HPV vaccine uptake: A meta-analysis and systematic review. Prev Med. 2021 Jul;148:106554.

31. Opel DJ, Mangione-Smith R, Robinson JD, Heritage J, DeVere V, Salas HS, et al. The Influence of Provider Communication Behaviors on Parental Vaccine Acceptance and Visit Experience. Am J Public Health. 2015 Oct;105(10):1998–2004.

32. Benkert R, Cuevas A, Thompson HS, Dove-Meadows E, Knuckles D. Ubiquitous Yet Unclear: A Systematic Review of Medical Mistrust. Behav Med. 2019 Apr-Jun;45(2):86–101.

33. Siegel M, Critchfield-Jain I, Boykin M, Owens A, Muratore R, Nunn T, et al. Racial/Ethnic Disparities in State-Level COVID-19 Vaccination Rates and Their Association with Structural Racism. J Racial Ethn Health Disparities. 2022 Dec;9(6):2361–74.

34. Nguyen KH, Yankey D, Lu P-J, Kriss JL, Brewer NT, Razzaghi H, et al. Report of Health Care Provider Recommendation for COVID-19 Vaccination Among Adults, by Recipient COVID-19 Vaccination Status and Attitudes - United States, April-September 2021. MMWR Morb Mortal Wkly Rep. 2021 Dec 17;70(50):1723–30.

35. Lo C-H, Chiu L, Qian A, Khan MZ, Alhassan HA, Duval AJ, et al. Association of Primary Care Physicians Per Capita With COVID-19 Vaccination Rates Among US Counties. JAMA Netw Open. 2022 Feb 1;5(2):e2147920.

36. Bateman LB, Hall AG, Anderson WA, Cherrington AL, Helova A, Judd S, et al. Exploring COVID-19 vaccine hesitancy among stakeholders in African American and Latinx communities in the deep south through the lens of the Health Belief Model. Am J Health Promot. 2022 Feb;36(2):288–95.

37. Balasuriya L, Santilli A, Morone J, Ainooson J, Roy B, Njoku A, et al. COVID-19 Vaccine Acceptance and Access Among Black and Latinx Communities. JAMA Netw Open. 2021 Oct 1;4(10):e2128575.

For peer review only

38. Dodson K, Mason J, Smith R. Covid-19 vaccine misinformation and narratives surrounding Black communities on social media October 13, 2021 [Internet]. First Draft News; 2021. Available from: https://firstdraftnews.org/long-form-article/covid-19-vaccine-misinformation-black-communities/

39. Momplaisir F, Haynes N, Nkwihoreze H, Nelson M, Werner RM, Jemmott J. Understanding Drivers of COVID-19 Vaccine Hesitancy Among Blacks. Clin Infect Dis [Internet]. 2021 Feb 9; Available from: http://dx.doi.org/10.1093/cid/ciab102

40. Kricorian K, Turner K. Covid-19 vaccine reluctance in older black and hispanic adults: Cultural sensitivity and institutional trust. J Am Geriatr Soc. 2021 Apr;69(S1):S214–S214.

41. Doherty IA, Pilkington W, Brown L, Billings V, Hoffler U, Paulin L, et al. COVID-19 vaccine hesitancy in underserved communities of North Carolina. PLoS One. 2021 Nov 1;16(11):e0248542.

42. Stancanelli J. Conducting an Online Focus Group. The Qualitative Report; Fort Lauderdale. 2010 May;15(3):761–5.

43. Turney L, Pocknee C. Virtual focus groups: New technologies, new opportunities, new learning environments [Internet]. ascilite.org; 2004 [cited 2022 Dec 19]. Available from: https://www.ascilite.org/conferences/perth04/procs/pdf/turney.pdf

44. Gordon AR, Calzo JP, Eiduson R, Sharp K, Silverstein S, Lopez E, et al. Asynchronous Online Focus Groups for Health Research: Case Study and Lessons Learned. International Journal of Qualitative Methods. 2021 Jan 1;20:1609406921990489.

45. Latkin CA, Edwards C, Davey-Rothwell MA, Tobin KE. The relationship between social desirability bias and self-reports of health, substance use, and social network factors among urban substance users in Baltimore, Maryland. Addict Behav. 2017 Oct;73:133–6.

46. Gorman JM, Scales DA. Leveraging infodemiologists to counteract online misinformation: Experience with COVID-19 vaccines. HKS Misinfo Review [Internet]. 2022 Jan 16; Available from: https://misinforeview.hks.harvard.edu/?p=8758

47. Scales D, Gorman JM, DiCaprio P, Hurth L, Radhakrishnan M, Windham S, et al. Community-oriented Motivational Interviewing (MI): A novel framework extending MI to address COVID-19 vaccine misinformation in online social media platforms. Comput Human Behav. 2023 Apr 1;141:107609.

48. Frechette J, Bitzas V, Aubry M, Kilpatrick K, Lavoie-Tremblay M. Capturing Lived Experience: Methodological Considerations for Interpretive Phenomenological Inquiry. International Journal of Qualitative Methods. 2020 Jan 1;19:1609406920907254.

49. Hsieh H-F, Shannon SE. Three approaches to qualitative content analysis. Qual Health Res. 2005 Nov;15(9):1277–88.

50. Shavers VL, Lynch CF, Burmeister LF. Knowledge of the Tuskegee study and its impact on the willingness to participate in medical research studies. J Natl Med Assoc. 2000 Dec;92(12):563–72.

For peer review only

51. Gorman JM, Gorman SE, Sandy W, Gregorian N, Scales DA. Implications of COVID-19 Vaccine Hesitancy: Results of Online Bulletin Board Interviews. Frontiers in Public Health [Internet]. 2022;9. Available from: https://www.frontiersin.org/article/10.3389/fpubh.2021.757283

52. Aw J, Seng JJB, Seah SSY, Low LL. COVID-19 Vaccine Hesitancy—A Scoping Review of Literature in High-Income Countries. Vaccines. 2021 Aug 13;9(8):900.

53. Rabaa J, Dorsaf H, Hend J, Sabrine O, Uta O, Yosra Z, et al. Perceptions of Tunisians on COVID-19 vaccines: A qualitative study on a sample of general population [Internet]. scivisionpub.com; 2021 [cited 2022 Dec 19]. Available from: http://scivisionpub.com/pdfs/perceptions-of-tunisians-on-covid19-vaccines-a-qualitative-study-on-a-sample-of-general-population-1843.pdf

54. Steinert JI, Sternberg H, Prince H, Fasolo B, Galizzi MM, Büthe T, et al. COVID-19 vaccine hesitancy in eight European countries: Prevalence, determinants, and heterogeneity. Sci Adv. 2022 Apr 29;8(17):eabm9825.

55. Jimenez ME, Rivera-Núñez Z, Crabtree BF, Hill D, Pellerano MB, Devance D, et al. Black and Latinx Community Perspectives on COVID-19 Mitigation Behaviors, Testing, and Vaccines. JAMA Netw Open. 2021 Jul 1;4(7):e2117074.

56. Sotero M. A Conceptual Model of Historical Trauma: Implications for Public Health Practice and Research [Internet]. Journal of health disparities research and practice. 2006 [cited 2022 Dec 19]. Available from: https://papers.ssrn.com/abstract=1350062

57. Akinyemi AA. Historical trauma compounds experiences of racial injustice. Nat Hum Behav. 2022 Sep;6(9):1183.

58. Bajaj SS, Stanford FC. Beyond Tuskegee - vaccine distrust and everyday racism. N Engl J Med. 2021 Feb 4;384(5):e12.

59. Murthy VH. Confronting Health Misinformation: The U.S. Surgeon General’s Advisory on Building a Healthy Information Environment [Internet]. Office of the US Surgeon General; 2021 Jul. Available from: https://www.hhs.gov/surgeongeneral/reports-and-publications/health-misinformation/index.html

60. Scales D, Gorman J. Screening for Information Environments: A Role for Health Systems to Address the Misinformation Crisis. J Prim Care Community Health. 2022 Jan;13:21501319221087870.

61. Vraga EK, Bode L. Defining Misinformation and Understanding its Bounded Nature: Using Expertise and Evidence for Describing Misinformation. Political Communication. 2020 Jan 2;37(1):136–44.

62. Southwell BG, Brennen JSB, Paquin R, Boudewyns V, Zeng J. Defining and Measuring Scientific Misinformation. Ann Am Acad Pol Soc Sci. 2022 Mar 1;700(1):98–111.

63. Joseph NP, Clark JA, Bauchner H, Walsh JP, Mercilus G, Figaro J, et al. Knowledge, attitudes, and beliefs regarding HPV vaccination: ethnic and cultural differences between

For peer review only

African-American and Haitian immigrant women. Womens Health Issues. 2012 Nov-Dec;22(6):e571-9.

64. Fu LY, Zimet GD, Latkin CA, Joseph JG. Social Networks for Human Papillomavirus Vaccine Advice Among African American Parents. J Adolesc Health. 2019 Jul;65(1):124–9.

65. Fu LY, Haimowitz R, Thompson D. Community members trusted by African American parents for vaccine advice. Hum Vaccin Immunother. 2019 Apr 11;15(7–8):1715–22.

66. Turchi J, Melton-Fant C. Media Framing of COVID-19 Racial Disparities: Lessons from Memphis, Tennessee. Sociology of Race and Ethnicity. 2022 Jul 1;8(3):355–69.

67. Tomori C, Evans DP, Ahmed A, Nair A, Meier BM. Where is the “Public” in American Public Health? Moving from individual responsibility to collective action. eClinicalMedicine [Internet]. 2022 Mar 1;45. Available from: https://doi.org/10.1016/j.eclinm.2022.101341

68. Gollust SE, Haselswerdt J. Who does COVID-19 hurt most? Perceptions of unequal impact and political implications. Soc Sci Med. 2023 Apr;323:115825.

69. Nguyen AL, Schwei RJ, Zhao Y-Q, Rathouz PJ, Jacobs EA. What Matters When It Comes to Trust in One’s Physician: Race/Ethnicity, Sociodemographic Factors, and/or Access to and Experiences with Health Care? Health Equity. 2020 Jun 29;4(1):280–9.

70. Dong L, Bogart LM, Gandhi P, Aboagye JB, Ryan S, Serwanga R, et al. A qualitative study of COVID-19 vaccine intentions and mistrust in Black Americans: Recommendations for vaccine dissemination and uptake. PLoS One. 2022 May 3;17(5):e0268020.

For peer review only

Table 1: Characteristics of bulletin board participants

	Participants from the Black community (N=30)	Participants from the Latinx community (N=30)
**Gender**		
Male	9	14
Female	21	16
**Age**		
18-29	4	13
30-39	8	8
40-49	7	6
50-59	6	3
60-69	5	0
**Marital Status**		
Married or living with partner	5	20
Divorced or widowed	6	0
Single	19	10
**Education Level**		
Less than high school	1	3
Some college	15	13
College degree	10	10
Post-graduate	4	4
**Household Income**		
Below $35,000	9	7
$35,000-$49,999	5	3
$50-$74,999	5	11
$75-$99,999	8	10
$100,000+	3	0
**Flu Vaccine History**		
Usually get the vaccine	4	3

For peer review only

Sometimes get the vaccine	12	13
Never get the vaccine	14	14
**Religion**		
Roman Catholic	NA	19
Protestant	NA	7
None	NA	3
Mormon	NA	1
**US or Foreign-Born**		
US-born	NA	17
Foreign-born	NA	13
**Heritage Country**		
Mexico	NA	8
Peru	NA	4
Ecuador	NA	4
Dominican Republic	NA	3
Venezuela	NA	2
Puerto Rico	NA	2
Colombia	NA	2
El Salvador	NA	1
Chile	NA	1
Costa Rica	NA	1
Cuba	NA	1
Guatemala	NA	1

**NA: Information not requested*

For peer review only

Table 2: Topics of inquiry and discussion on the bulletin board

Day	Topics
1	General health and wellbeing concerns for themselves and their families<br>Sources of health and medical information and advice<br>Primary care doctors<br>Use of trusted family and home remedies<br>Personal experiences with vaccines in the past<br>Experiences with flu vaccines<br>Awareness of messaging around vaccine safety<br>Preferred sources of information<br>Use of social media for medical or health information
2	Things they have heard about the COVID-19 vaccines<br>How much they trust the sources<br>What, if anything, frightens them about a COVID-19 vaccine<br>Which is more frightening to them, catching COVID-19 or getting a vaccine<br>Intentions regarding a COVID-19 vaccine<br>Perceived effectiveness of the COVID-19 vaccines<br>If and how they have discussed the vaccine with their doctors<br>If and how they have discussed the vaccine with family members or friends<br>What their community and church leaders are advising them with respect to the vaccine<br>What they have heard about vaccines and vaccine safety on social media<br>How much they trust what they see on social media<br>What public health officials are saying<br>How much trust they place in public health officials
3	How have their communities and their families been affected by COVID-19<br>How worried are they about possibly passing COVID-19 on to at-risk members of their families<br>How important do they feel it is to eventually receive a COVID-19 vaccine<br>How important are vaccines for restoring normalcy<br>What are the best arguments they have heard in favor of vaccination<br>How do they feel about the idea of mandated vaccination<br>What information would make them feel better about getting a COVID-19 vaccine<br>Whose endorsement of vaccination would be meaningful for them<br>Responses to various pro-vaccine messages

Table 3: Example quotations of hesitancy related to safety and efficacy concerns.

**Sub-Theme**	**Quotations**
Vaccines are a "black box"	*"I don't know what they're putting in my body."*
Fears about vaccine safety	*"Vaccines lower the fear of COVID, but not the fear of long-term effects"*<br><br>*"The unknown frightens me. What happens when the vaccine interacts with medications… what happens years from now?"*<br><br>*"What frightens me is that uncertainty. No one knows what this vaccine will do to humans long term. Let alone babies that are born after."*
Conviction that the vaccine can kill you	*"I believe I would say receiving the vaccine is most frightening. I have had several people to pass away [sic] after receiving the vaccine. Prior to the vaccine these individuals were healthy and doing fine."*<br><br>*"The idea that I could die or have health complications because of the vaccine frightens me. I've mostly read this in social media."*
Concerns about scientific uncertainty	*"There are so many conflicting reports that it is difficult to know who is being honest and factual."*
Vaccines are not effective	*"With all the reports of fully vaccinated people contracting COVID a second time I'm not convinced that the vaccine offers the protection it claims."*

For peer review only

	"From what I'm hearing it would be very effective, but some people, even though they got vaccinated ended up with COVID. I'm not really sure at the moment to be honest with you"
Vaccines are insufficient	*"Currently, only 50% effective. I have seen where there's a booster shot required every six months. I've also heard doctors and CDC state that it doesn't prevent you from getting COVID, it just lessens your likelihood the virus being as bad."*
Vaccines do not prevent transmission	*"Some people that have been vaccinated have gotten the virus. I think that it causes people to lower their guard regarding social distancing and wearing masks. New more contagious strains of the virus are still popping up."*<br><br>*"They're dying from the vaccine as well. And the vaccine is not effective. They still get the virus and pass it on to other people."*

For peer review only

Table 4: Example quotations of hesitancy related to the perception that vaccines are not worth the risk.

Sub-Theme	Quotations
Vaccines are riskier than the virus	*"I have heard the COVID virus isn't too bad and I have multiple friends that have had it. So, I guess you can say I'm more worried about the vaccine than the virus itself."*<br>*"I have already had the virus and had minimal symptoms. So I guess I could say getting the vaccine is more frightening."*<br>*"Both are scary, but getting the vaccine is more frightening for me because I feel that if I got COVID I would be fine and it wouldn't affect me much."*<br>*"At this point, me getting the COVID vaccine is more frightening [than getting COVID]. I stay by myself; I only go out if need be and I am masked up."*
Vaccines are not necessary	*"I'm not frightened at all because I take great precautions. I'm more concerned about someone passing it on to me."*
Vaccines are only for the most vulnerable	*"For me personally, I don't feel like it's necessary as I am a healthy individual with no underlying health issues, and so is my husband and child."*<br>*"I think that the vaccine is important for those who are most vulnerable. If they get sick, at least it won't be as serious."*

For peer review only

Table 5: Example quotations of hesitancy related to distrust of institutions or concerns about structural/individual racism.

Sub-Theme	Quotations
Limited trust in physicians	*"I never really trust one opinion regarding health issues. I listen to what the doctors say and suggest for any illness. Next I read all information given and search the internet for reliable sources and try to gain an understanding of the situation. At that point my decision is made."*<br><br>*"I don't view my doctor as an expert in vaccine… Kinda like having a degree in general studies vs. a specialist… I think he's knowledgeable… but don't think the level of focus and concentration points to expert."*<br><br>*"I don't think anyone is an expert. You can't know everything about such a new vaccine!"*<br><br>*"In addition to the advice of medical professionals, I also believe firmly… in the power of being natural and how people used to cure themselves in the past… The traditional remedies work."*
Lack of trust in government	*"I have a hard time trusting anything government affiliated – because they follow government directives rather than their own expertise."*<br><br>*"I don't have confidence in what the government says in general. At the end of the day they are protecting themselves and I don't believe that they are concerned about those in the lowest classes. I feel like the government if [sic] capable of lying for its own benefit."*
Limited trust in public health authorities	*"I trust most of their opinions. Not all."*<br><br>*"I trust but may not do 100% of what they say."*
Concerns about health outcomes differing by race or ethnicity	*"I'm not convinced that being Black does affect the risks of getting COVID. I know that's what reported but I'm just not convinced that it's true….. It's not the news itself that's unbelievable, it's the source. Medical institutions have subjected Black people to abuse, exploitation and experimentation since this country's foundation. It wouldn't be the first time that Black people were misled into getting vaccines with the false hope of immunity from a deadly disease."*

For peer review only

	"I feel that my community is more at risk of catching COVID due to the history of us being ignored by health professionals and the government. Additionally, we are most likely in employment opportunities that expose us to conditions that are not ideal. I don't agree that we are experiencing more serious reactions because that implies that we are unhealthy. Unhealthy behaviors are common in America and not assigned to simply one community. If we are having serious reactions, it is most likely due to our concerns being brushed aside when we seek assistance from health care workers."<br><br>*"I honestly believe that the social structure of how Black people are treated in America is more so to do with the severity of the virus to this group. Less readily available access to health care, poor living situation, less money funneled into Black community…."*<br><br>*"I don't feel like my race affects my risk of getting [COVID] but I feel like it would affect the medical care that I received if I needed medical care while I was positive."*<br><br>*"I don't think it affects people differently due to ethnicity."*<br><br>*"I don't think that race is a factor here. Anyone can get the virus."*

For peer review only

Critica Bulletin Board Discussion Guide: Exploring Hesitancy to Vaccinate Among Latinx and African-American Communities

May 27, 2021

All questions will be partially masked (respondents can only see others' responses once they have submitted their own response). All questions will be text response (open-ended) questions unless otherwise indicated.

Introduction and Instructions:

[Multiple choice question] Welcome! My name is **Marsha**, and I will be guiding you through our online discussion over the next three days. Before we get started, please read the following information carefully and consent to participate in the discussion.

[INSERT CONSENT TEXT]

( ) Yes
( ) No [Terminate]

[Notice] Here's how the discussion will work…

Over the next three days, I will ask you questions related to the topic of vaccines. My job is to get your thoughts and opinions about this topic, and your job is to share them by typing your responses, and then submitting them. I am interested in your **honest** opinions, be they positive or negative, so please share freely and honestly throughout the discussion. There are no "right" or "wrong" answers, and you don't have to worry about your spelling or sentence structure. If you share your honest opinions and attitudes, then you will be providing the "right" answers.

For peer review only - http://bmjopen.bmj.com/site/about/guidelines.xhtml

Once you submit your response to a question, you will be able to see how the rest of the group has responded and you can "like" or comment on their posts if you have anything to add. We are all different individuals in this discussion, so I expect there will be a variety of different experiences and opinions. That's great! If it looks to you like you have a different opinion than most others, please be sure to share it! It is important that you all be honest, and respectful of others who might think differently than you do.

I'm hoping to learn as much from you as possible, and encourage you not only tell me **what** you think, but **WHY**. The more details you share to explain your opinions, the better! This is not like Twitter or other social media where there's a limit to how much you can write. In fact, it's just the opposite! Tell me as much as you want in response to each question, especially WHY your thoughts and opinions are what they are. Please share examples or experiences that you have had that help to explain your response. For example, "Just last week, I was going to the grocery store, and…." Or "My niece told me that one of her teachers said...." Stories or examples like this really help me to understand what shapes your opinions.

I will be posting new questions each day – once in the morning, and once in the early afternoon, so it is important that you visit the website at least twice every day. It will probably take you about 30 minutes per day to answer the questions. You can spend as much time on the site as you desire.

To answer a question or respond to a post, simply click on the button in the bottom left corner that says "You have not replied. Click here," type your response in the space provided, and click on the "Submit" button. Please completely answer each question fully and be as conversational as possible, details are great! Any questions that you have not answered will have a button stating "You have not replied. Click here". This way you can easily tell what you still need to answer.

Keep an eye out for follow-up questions from me. I may ask questions of you specifically or to the group as a whole. To easily see if you have follow-ups you can

For peer review only - http://bmjopen.bmj.com/site/about/guidelines.xhtml

look at your Project Alerts box on the left of your screen and if you see a number beside "Unanswered Follow Up Questions" you'll know I have put something in specifically for you. Just click on that number and it will be brought up on the screen for you.

Here are some more tips for you to make this process easy and enjoyable:

- Please watch the "Participant Intro Video" found on the "Dashboard (Home)" page of this site. There is a lot of really great information in there to help you out along the way!
- Please upload a photo or an avatar of yourself so that I can associate a face with your name. It just makes our discussion a little more personable. You can do this under "My Profile" in the upper left-hand corner of the site.
- You can click "Highlight Unanswered Questions" on the top of the Navigation Section to see if there are any questions from me that you may have missed. To make things even easier…on the left-hand side of the screen you'll see the number of questions or follow ups that I may have left for you! Just look for the blue box titled "Project Alerts."
- On the left of your screen you'll see a tab titled "Message Center". Click on that feature to see any emails that I might have sent to you that you might have missed in your personal email inbox.
- There are some really good help articles and "How-To's" HERE if you run into questions about how to use QualBoard (you can also click "Help Desk" on the top right of your screen to get to this site).

If you have technical difficulties of any kind while participating in this discussion, please click "Chat with QualBoard Support" at the bottom left of your screen. A technical representative will reply promptly during normal business hours (within 24 hours of your request).

One other thing -- typos don't matter, we all make them!

For peer review only

DAY 1:

Health and Well-being Concerns

- Please take a moment to introduce yourself. Your first name only is fine. I'd like to know where you live (city, state), and who all lives at home with you, if anyone.
    - Do you have any children who live with you?
    - Do you have any elderly people who live with you, or who you take care of?

- When it comes to your health, what sorts of things are you concerned about? Please describe all of the concerns that you think about, specific to your own health, from time to time.

- What kinds of actions or precautions do you take to address these concerns, if any? Please explain for each health concern that you named.

- What about your family's health – what heath concerns, if any, do you have for others in your family? Please explain in detail.

- What kinds of actions or precautions do you take to address these concerns, if any? Please explain for each health concern that you named.

Sources of Health and Medical Information and Advice

- When it comes to your personal health, where do you turn for information and advice? Please list all of the sources you turn to.

- Who or what do you trust when it comes to health or medical advice? What makes this source/these sources trustworthy? Please explain in detail for each one.

- Who, if anyone, do you trust about health and medical issues aside from doctors or nurses? Please explain what makes you trust each of these people.

- Do you ever do your own research on health topics? If so, where do you start and what sources do you like to use? Please walk me through your process, in detail.

- What happens when you come across health-related information that contradicts other sources of health information? How do you decide what to believe or what to act upon? Please explain in detail, and share examples that may come to mind.

- Do you have a personal or family physician that you go to? If so, what are your thoughts about your doctor? Please describe your level of comfort sharing health concerns with your doctor.

    - If you do not have a regular family doctor or primary care physician, where do you go for medical advice or for medical treatment?

For peer review only - http://bmjopen.bmj.com/site/about/guidelines.xhtml

- How would you describe your level of trust with your personal physician? Why is that? Please explain your answer.

- Are there times you find your doctor's advice convincing and other times you question what he or she tells you? Please describe these situations in detail.

- Can you think of a time when you questioned your doctor's advice? Please explain that situation in detail.
    - Did you talk to your doctor about your concerns about this advice?
    - Did you follow the advice anyway, or did you do something else?

- Are there products or home remedies you like to use to stay healthy or to recover from illness? If so, please share examples of these, including when and why you use them.
    - How did you decide that this product or remedy was right for you? Please explain in detail.

For peer review only

Experiences and Attitudes with Respect to Vaccines in General

Welcome back! I'd like to learn about your experiences with getting vaccines, and what you think of vaccines in general. We will talk specifically about the Covid vaccine later in this discussion. For now, I'm interested in **other kinds of vaccines, not the Covid vaccine**, starting with ones you may have gotten when you were a child.

- Think back to when you were a child. Do you recall your parents taking you to get vaccinations – such as vaccinations for measles, tetanus, diptheria, etc.? What do you remember about these experiences?
    - Were these vaccines required where you grew up?

- For those of you with young children, have you taken them to get the standard vaccines for their age group?
    - What are your thoughts about these recommended vaccines for children?
    - How important do you find such vaccines to be?

- As an adult, which vaccines have you received (other than the Covid vaccine)? Please list them, and explain why you chose to get each one.

- How necessary have you found flu vaccines to be for your own health and well-being during the flu season?

- How regularly have you gotten flu shots in recent years?

- If you get the flu shot some years, but not others, what is it that makes you decide to get it some years, but not others?

For peer review only - http://bmjopen.bmj.com/site/about/guidelines.xhtml

  - If anything prevented you from getting a flu shot in past years, please explain what it was?

- How much do you typically worry about getting the flu?

- Is there anything that makes you hesitant to get a flu shot? If so, what? Please explain in detail.

- Have you ever had a bad experience with a vaccine? If so, please describe what happened in detail.

- Have you read or heard anything in the past about the safety of vaccines (not including the COVID vaccine) or the need for vaccines, in general? If so, please explain in detail what the safety concerns are that you have heard or read.

  - Where did you hear or read this information?

  - How credible did you find this information about the safety of vaccines or the need for vaccines? Why is that?

  - What, if anything, did you do to try to confirm what you heard or double-check the information?

- Have you ever heard contradictory messages on the safety of vaccines? For example, some sources saying that they are safe and others saying that they are unsafe? Please explain.

- Where or from whom do you usually get your news or information on vaccines? Please be as specific as possible.

  - How much do you trust these sources of information? Please explain why you may or may not trust each one.

- What social media platforms, if any, do you use regularly?

- What impact do you think these social media platforms have had on your feelings and concerns around vaccines? Please explain and use examples, if relevant.

- Do you know of or follow any social media groups that have been talking about vaccines, or sharing information about them?

  - If so, what do you think about the information shared in these groups? How do these groups make you feel?

- When it comes to the need for vaccines and the safety of vaccines, how do you decide which side of the debate to trust? Please explain in detail.

For peer review only - http://bmjopen.bmj.com/site/about/guidelines.xhtml

- How do you decide if someone talking about vaccines is an expert in this area or not?
    - What makes someone an expert in the area of vaccines and vaccine safety? Please explain your opinion in detail.
- For those of you with a primary care physician, do you consider that doctor to be an expert on vaccines? Why or why not?
- Are there some vaccines you trust and others you don't? If so, how do you decide? Please explain in detail.

That's all of the questions for today. Thank you for sharing your thoughts and opinions so far! I look forward to learning more from you in the next session!

DAY 2:

Welcome back! At this point, I'd like to turn our conversation to the COVID vaccines that are available.

When it comes to advice and information on Covid vaccines, you may be hearing different things from many different sources – from news stations, local health officials, national health officials, church leaders, community groups, community leaders, websites, and YouTube channels, among others. We'd like to explore which of these sources of information you rely on or trust more and which you rely on or trust less.

- What kinds of things have you heard about the COVID-19 vaccines that have been developed?
- Have you heard or read anything that makes you concerned regarding the safety or the effectiveness of any of the new COVID vaccines?
    - If so, what specifically have you heard that concerns you? Please explain in detail, and give examples.
    - Where did you hear or read this?
- What about the information or source made you trust it?
- What most frightens you about getting a COVID vaccine?
- Which to you is more frightening: getting a Covid vaccine or getting the COVID-19 virus itself?

For peer review only - http://bmjopen.bmj.com/site/about/guidelines.xhtml

- How frightened are you that you might pass Covid along to people you care about?

- Does what you've heard make you think you might want to get the vaccine eventually – if your concerns are addressed? Why or why not?

- MULTIPLE CHOICE. Based on what you have seen and heard about the Covid vaccines so far, which of the following describes your attitude towards getting a Covid vaccine?

  - ❑ I will definitely get it as soon as I can.
  - ❑ I will probably get it as soon as I can.
  - ❑ I will probably get it eventually but want to wait until I know it definitely works.
  - ❑ I will probably get it eventually but want to wait until I know it is safe.
  - ❑ I will probably not get it.
  - ❑ I will definitely not get it.

- How necessary is getting vaccinated in preventing you from getting sick or dying from Covid?

- How effective do you think the vaccine would be in protecting you from Covid?

- Are there other, practical reasons that are preventing you or people you know from getting a Covid vaccine, or making it harder to do so?

- Do you have people in your family or community who feel they may not be eligible to get a Covid vaccine? If so, why do they think they wouldn't be eligible?

- Who else is involved in your decision and ability to get a Covid vaccine? Would you need to get permission from anyone else in the family before you are could get a vaccine?

- Is there disagreement within your family – some family members who want to get a Covid vaccine and others who don't? If so, how will that be resolved?

- Have you ever discussed the Covid vaccine or expressed your concerns about it with a medical professional, such as a doctor or pharmacist? If not, why not? If so, did they advise you to get a vaccine? What reasons did they give?

- Do you know people who have been vaccinated against Covid? Do you have friends who have been vaccinated? If so, how has this affected your thinking about the vaccine?

For peer review only - http://bmjopen.bmj.com/site/about/guidelines.xhtml

- If you have a church you belong to, have church leaders made any recommendations about the Covid vaccine? If so, what have they recommended?

- How important to you is the advice of church leaders on this subject?

- How do you weigh their recommendations against those of health professionals if they contradict one another?

- What about local community groups? What kinds of messages have you heard from community centers or other local organizations about the vaccine?

- How important or relevant to you are their recommendations in this regard?

- What kind of things do you hear from people in your neighborhood/community? Are many people in your community outspoken about the Covid vaccine and whether or not it is safe?

- And what about social media, what do you hear about the Covid vaccine on the social media sites you use?

- How much do you trust things people are posting on social media about the Covid vaccine?

- Can you give an example of a source you trust on social media?

- What about the Pharmaceutical companies that developed the Covid vaccines – how much do you generally trust their products?

- Have you heard anything that makes you question their development of the Covid vaccines?

- What about your local or state public health officials? What has been their advice regarding the Covid vaccines?

- Do you find their statements trustworthy or re-assuring? Why or why not?

- Do you find advice from local health officials to be more relevant or compelling than advice from national health officials? Why or why not?

- How much do you trust the recommendations or advice of public health leaders in the government, such as Dr. Anthony Fauci, Director of the National Institute of Allergy and Infectious Diseases and member of the White House Coronavirus Task Force?

- What about the Centers for Disease Control (CDC)? How familiar are you with this organization?

For peer review only - http://bmjopen.bmj.com/site/about/guidelines.xhtml

- Had you heard of it before the Covid pandemic?
- Do you trust its recommendations on how to protect yourself against Covid?
- Has your trust in the recommendations of the CDC changed over the course of the pandemic? Is so, how?
- How important is it to you to know that the FDA (the U.S. Food and Drug Administration) had authorized the new vaccine to feel it would be safe for you to get?
  Does this reassure you as to the safety of the Covid vaccines?
- Have you heard anything that makes you question the FDA's authorization of the Covid vaccines?
- Are there any reasons why you find government reassurances about health issues like Covid to be unconvincing?

DAY 3:

- How has your community been affected by Covid?
- How has Covid affected the neighborhood you live in? Has it seen a lot of cases?
- Have you heard of people around you getting seriously ill or dying from Covid?
- How has your family been affected? Do you have family members or close friends who have gotten seriously ill from Covid? How does this affect your thinking about the value of getting a vaccine?
- How worried are you about catching Covid? Does the idea of getting Covid frighten you?
- How frightening is the idea of your parents or other older family members getting it?
- Have you caught Covid? If so, how bad was it? If so, how does this make you feel about getting vaccinated?
- How do you feel that being Black/African-American affects your risk of catching Covid or of having serious reactions to Covid?
- Do you know of people here who want to get a Covid vaccine but are having trouble getting it?

For peer review only - http://bmjopen.bmj.com/site/about/guidelines.xhtml

- How does this influence your thinking about the Covid vaccine?
- Does this make you feel lucky or privileged to live in a country and a time when it is possible to be vaccinated against a potentially deadly disease?
- How much do you feel that it is a privilege to be able to get vaccinated, given that there are many countries where people desperately want it and can't get it.
- Do you have any religious objections to the vaccines? Has any clergy person discouraged you from getting vaccinated? IF SO, PROBE RE: nature of the objection or the reason for the discouragement
- How important do you feel it is to sooner or later get a Covid vaccine for your own health?
- How important do you feel it is to get a vaccine to help protect your family members or others you come in contact with who might be very vulnerable to having a serious reaction to Covid?
- How important do you feel it is for people to get a Covid vaccine to help restore normalcy??
- Do you think certain people should be required to get them? Why or why not?
- Do you think everyone should be required to get them? Why or why not?
- Who or what do you think would convince you that a Covid vaccine is safe?
- What are the best arguments you have heard for getting the vaccine?
- Even if you don't think you personally need it, are there other reasons you might want to get it? If so, please explain.
- To what extent should it be a matter of individual need or choice versus a matter of community responsibility?
- What would make you feel better or more confident of the safety of Covid vaccines?
- Whose endorsement of vaccination would you need to see?
- Would it have to be a leader in your community? Someone in your family? Someone in your church?

For peer review only - http://bmjopen.bmj.com/site/about/guidelines.xhtml

- As an African American, whose endorsement of a Covid vaccine would carry the most weight for you? Is there an individual or a group or organization that place a great deal of trust in with regards to this issue?

- Now I'm going to present some facts about the Covid vaccine, and I'd like to get your reaction acts and hear how these facts might affect your thinking.

- How does it affect your thinking about Covid vaccines that it helps protect the most vulnerable family members and community members?

- How does it affect your thinking about Covid vaccines that doctors and healthcare workers trust the vaccine and are getting them.

- How does it affect your thinking that hundreds of millions have now gotten the vaccine safely?

- How does it affect your thinking to see that getting vaccinated is helping us get our normal lives back, and ending social isolation?

- What part do you think vaccination should play in helping us open up and return to normal, and eliminating ongoing fear of Covid.

Thank you all for your participation and for sharing your thoughts. Is there anything else about the Covid vaccine that you would like to share before we close?

For peer review only

For peer review only - http://bmjopen.bmj.com/site/about/guidelines.xhtml

Standards for Reporting Qualitative Research (SRQR)*

http://www.equator-network.org/reporting-guidelines/srqr/

Title and abstract

	Page/line no(s).
**Title** - Concise description of the nature and topic of the study Identifying the study as qualitative or indicating the approach (e.g., ethnography, grounded theory) or data collection methods (e.g., interview, focus group) is recommended	1
**Abstract** - Summary of key elements of the study using the abstract format of the intended publication; typically includes background, purpose, methods, results, and conclusions	2

Introduction

**Problem formulation** - Description and significance of the problem/phenomenon studied; review of relevant theory and empirical work; problem statement	4-6
**Purpose or research question** - Purpose of the study and specific objectives or questions	4-6

Methods

**Qualitative approach and research paradigm** - Qualitative approach (e.g., ethnography, grounded theory, case study, phenomenology, narrative research) and guiding theory if appropriate; identifying the research paradigm (e.g., postpositivist, constructivist/ interpretivist) is also recommended; rationale**	10-11
**Researcher characteristics and reflexivity** - Researchers' characteristics that may influence the research, including personal attributes, qualifications/experience, relationship with participants, assumptions, and/or presuppositions; potential or actual interaction between researchers' characteristics and the research questions, approach, methods, results, and/or transferability	7-9
**Context** - Setting/site and salient contextual factors; rationale**	7
**Sampling strategy** - How and why research participants, documents, or events were selected; criteria for deciding when no further sampling was necessary (e.g., sampling saturation); rationale**	7-9
**Ethical issues pertaining to human subjects** - Documentation of approval by an appropriate ethics review board and participant consent, or explanation for lack thereof; other confidentiality and data security issues	7
**Data collection methods** - Types of data collected; details of data collection procedures including (as appropriate) start and stop dates of data collection and analysis, iterative process, triangulation of sources/methods, and modification of procedures in response to evolving study findings; rationale**	7-9

For peer review only - http://bmjopen.bmj.com/site/about/guidelines.xhtml

For peer review only

**Data collection instruments and technologies** - Description of instruments (e.g., interview guides, questionnaires) and devices (e.g., audio recorders) used for data collection; if/how the instrument(s) changed over the course of the study	7-9
**Units of study** - Number and relevant characteristics of participants, documents, or events included in the study; level of participation (could be reported in results)	7-9, Table 1
**Data processing** - Methods for processing data prior to and during analysis, including transcription, data entry, data management and security, verification of data integrity, data coding, and anonymization/de-identification of excerpts	7-9
**Data analysis** - Process by which inferences, themes, etc., were identified and developed, including the researchers involved in data analysis; usually references a specific paradigm or approach; rationale**	10-11
**Techniques to enhance trustworthiness** - Techniques to enhance trustworthiness and credibility of data analysis (e.g., member checking, audit trail, triangulation); rationale**	10-11

Results/findings

**Synthesis and interpretation** - Main findings (e.g., interpretations, inferences, and themes); might include development of a theory or model, or integration with prior research or theory	11-14
**Links to empirical data** - Evidence (e.g., quotes, field notes, text excerpts, photographs) to substantiate analytic findings	Table 3

Discussion

**Integration with prior work, implications, transferability, and contribution(s) to the field -** Short summary of main findings; explanation of how findings and conclusions connect to, support, elaborate on, or challenge conclusions of earlier scholarship; discussion of scope of application/generalizability; identification of unique contribution(s) to scholarship in a discipline or field	15-19
**Limitations** - Trustworthiness and limitations of findings	19-20

Other

**Conflicts of interest** - Potential sources of influence or perceived influence on study conduct and conclusions; how these were managed	1
**Funding** - Sources of funding and other support; role of funders in data collection, interpretation, and reporting	1

*The authors created the SRQR by searching the literature to identify guidelines, reporting standards, and critical appraisal criteria for qualitative research; reviewing the reference lists of retrieved sources; and contacting experts to gain feedback. The SRQR aims to improve the transparency of all aspects of qualitative research by providing clear standards for reporting qualitative research.

For peer review only - http://bmjopen.bmj.com/site/about/guidelines.xhtml

**The rationale should briefly discuss the justification for choosing that theory, approach, method, or technique rather than other options available, the assumptions and limitations implicit in those choices, and how those choices influence study conclusions and transferability. As appropriate, the rationale for several items might be discussed together.

Reference:

O'Brien BC, Harris IB, Beckman TJ, Reed DA, Cook DA. **Standards for reporting qualitative research: a synthesis of recommendations.** *Academic Medicine*, Vol. 89, No. 9 / Sept 2014 DOI: 10.1097/ACM.0000000000000388

For peer review only

BMJ Open

"They've all endorsed it…but I'm just not there:" A qualitative exploration of Covid-19 vaccine hesitancy reported by Black and Latinx individuals

Journal:	*BMJ Open*
Manuscript ID	bmjopen-2023-072619.R2
Article Type:	Original research
Date Submitted by the Author:	28-Jun-2023
Complete List of Authors:	Scales, David; Weill Cornell Medical College; Critica<br>Gorman, Sara; Critica<br>Windham, Savannah; Critica<br>Sandy, William; Fluent Research<br>Gregorian, Nellie; Fluent Research<br>Hurth, Lindsay; Critica<br>Radhakrishnan, Malavika; Critica<br>Akunne, Azubuike; Critica<br>Gorman, Jack M.; Critica
**Primary Subject Heading**:	Sociology
Secondary Subject Heading:	Infectious diseases, Health services research
Keywords:	COVID-19, Anthropology < TROPICAL MEDICINE, Patient Participation, PREVENTIVE MEDICINE

SCHOLARONE™
Manuscripts

For peer review only - http://bmjopen.bmj.com/site/about/guidelines.xhtml

I, the Submitting Author has the right to grant and does grant on behalf of all authors of the Work (as defined in the below author licence), an exclusive licence and/or a non-exclusive licence for contributions from authors who are: i) UK Crown employees; ii) where BMJ has agreed a CC-BY licence shall apply, and/or iii) in accordance with the terms applicable for US Federal Government officers or employees acting as part of their official duties; on a worldwide, perpetual, irrevocable, royalty-free basis to BMJ Publishing Group Ltd ("BMJ") its licensees and where the relevant Journal is co-owned by BMJ to the co-owners of the Journal, to publish the Work in this journal and any other BMJ products and to exploit all rights, as set out in our licence.

The Submitting Author accepts and understands that any supply made under these terms is made by BMJ to the Submitting Author unless you are acting as an employee on behalf of your employer or a postgraduate student of an affiliated institution which is paying any applicable article publishing charge ("APC") for Open Access articles. Where the Submitting Author wishes to make the Work available on an Open Access basis (and intends to pay the relevant APC), the terms of reuse of such Open Access shall be governed by a Creative Commons licence – details of these licences and which Creative Commons licence will apply to this Work are set out in our licence referred to above.

Other than as permitted in any relevant BMJ Author's Self Archiving Policies, I confirm this Work has not been accepted for publication elsewhere, is not being considered for publication elsewhere and does not duplicate material already published. I confirm all authors consent to publication of this Work and authorise the granting of this licence.

For peer review only

"They've all endorsed it…but I'm just not there:" A qualitative exploration of COVID-19 vaccine hesitancy reported by Black and Latinx individuals

David Scales,[1,2] Sara Gorman,[2] Savannah Windham,[2] William Sandy,[3] Nellie Gregorian,[3] Lindsay Hurth,[2] Malavika Radhakrishnan,[2] Azubuike Akunne,[2] & Jack M. Gorman[2]

1. Section of Hospital Medicine, Division of General Internal Medicine, Department of Medicine, Weill Cornell Medicine, New York, NY, United States
2. Critica, Bronx, NY, United States
3. Fluent LLC, New York, NY, United States

Word Count: 5632

Corresponding Author:
David Scales
david.scales@aya.yale.edu
525 East 68th Street
New York, NY 10068

For peer review only

Abstract

Objective We sought to examine reasons for vaccine hesitancy among online communities of US-based Black and Latinx communities to understand the role of historical racism, present-day structural racism, medical mistrust, and individual concerns about vaccine safety and efficacy.

Design A qualitative study using narrative and interpretive phenomenological analysis of online bulletin board focus groups.

Setting Bulletin boards with a focus-group like setting in an online, private, chat-room-like environment

Participants Self-described vaccine hesitant participants from US-based Black (30) and Latinx (30) communities designed to reflect various axes of diversity within these respective communities in the US context.

Results Bulletin board discussions covered a range of topics related to COVID-19 vaccination. COVID-19 vaccine hesitant participants expressed fears about vaccine safety and doubts about vaccine efficacy. Elements of structural racism were cited in both groups as affecting populations but not playing a role in individual vaccine decisions. Historical racism was infrequently cited as a reason for vaccine hesitancy. Individualized fears and doubts about COVID-19 (short- and long-term) safety and efficacy dominated these bulletin board discussions. Community benefits of vaccination were not commonly raised among participants.

Conclusions While this suggests that addressing individually-focused fear and doubts are central to overcoming COVID-19 vaccine hesitancy in Black and Latinx groups, addressing the effects of present-day structural racism through a focus on community protection may also be important.

Keywords
COVID-19 Vaccines, Vaccination Hesitancy, Black, Latinx, Infodemic, Pandemics

review only

Strengths and limitations of this study

- We employed online bulletin board groups of US-based Black and Latinx participants selected for self-described vaccine hesitancy to understand the reasons and motivations behind their stance, leveraging the online design to attract participants whose information environments consist of high proportions of online content where anti-vaccine misinformation was prominent.
- Compared to in-person focus groups, asynchronous bulletin boards allow all participants to freely express themselves with fewer concerns about speaking too much while facilitators can encourage greater participation from those who are more quiet.
- Transcripts were analyzed using various qualitative techniques, including narrative and interpretive phenomenological analysis to allow for understanding recurrent themes.
- Participants revealed that vaccine hesitancy is the result of a confluence of psychological and social considerations, but with selective focus on certain factors over others as participants weighed risks and benefits, such as high emphasis was placed on individual vaccine safety with relatively little attention to potential community-level benefits of vaccination.
- Vaccine safety and efficacy were of highest concern; however, mistrust in institutions and concerns about systemic and personal racism also featured prominently among participants' concerns.

For peer review only

Introduction

For much of the COVID-19 pandemic, rates of COVID-19 vaccination in Black and Latinx communities in the United States were lower than White communities, although the gap appears to be narrowing (1–3). This vaccination gap is especially concerning because Black and Latinx people diagnosed with COVID-19 have experienced worse clinical outcomes (4). Structural and social determinants of health like racism, socioeconomic status, access to transportation, and access to information or trusted healthcare practitioners make it difficult for people in some communities who want to be vaccinated to get vaccines, but even as vaccine uptake gaps have narrowed, a number of people continue to choose not to be vaccinated (5–7).

COVID-19 vaccine hesitancy, defined as "indecision around accepting a vaccination" (8), among Black and Latinx people has been found in survey studies to be higher than among White people (9–11). In their review of 13 studies of racial and ethnic disparities in COVID-19 vaccination status, Khubchandani and Macias[9] found an overall pooled rate of vaccine hesitancy of 26.3%, but a higher rate among Hispanic (30.2%) and African American (40.6%) study participants (12). Even among healthcare workers, COVID-19 vaccine hesitancy was found to be higher among Black and Latinx people compared with White people (13).

The literature on COVID-19 vaccine hesitancy among Black and Latinx people highlights three influential factors affecting vaccine decisions in these populations: individual fears and concerns about vaccine adverse side effects and efficacy, historical mistreatment in medical/scientific contexts (e.g., the US Public Health Service syphilis study at Tuskegee), present-day experience with mistreatment by the healthcare system, (including structural racism), the latter two of which are linked to mistrust in healthcare providers and the healthcare system. The literature on each of these topics is vast and a full review is beyond the scope of our study. However, there are some key studies worth highlighting that informed and motivated the research presented here.

Survey data have shown that concerns about vaccine safety and efficacy are associated with higher rates of vaccine hesitancy among Black and Latinx people (14,15). In a survey using U.S. census data, Black people were more likely than white people to develop COVID-19 vaccine hesitancy because of lack of confidence in the safety and efficacy of vaccines and because of a tendency to watch evolving information and wait before considering vaccination, though this group saw the greatest percentage drop in hesitancy over time (16). Longoria and colleagues found fears about COVID-19 vaccine safety were commonly circulated online among Latinx people, as well as narratives about alleged “alternative treatments” (17). Recent work by Morales and Paat (2022) provides additional evidence of a “watch and wait” approach among Black Americans, noting how rates of vaccine hesitancy and refusal in this community declined over time while it remained stable in White communities (18), consistent with more people seeking the vaccine as more time passes demonstrating the safety and efficacy of the vaccine.

Early in the vaccine roll-out, there was significant concern among public health professionals that well-known narratives of historical racism among marginalized communities around, for example, Henrietta Lacks (19) or the syphilis study at Tuskegee (20), would engender vaccine hesitancy due to a “legacy of distrust” in medical research (21). However, much empirical research highlighted that past narratives around Henrietta Lacks or the unethical syphilis experiments performed in Tuskegee relate to and shape contemporary lived experiences, perceptions of racism in medicine, and mistrust in the healthcare system in general and COVID-19 vaccines specifically (22). Using survey data, Martin, Stanton and Johnson (23) found that current mistreatment by the healthcare system, rather than historical mistreatment as exemplified by the Tuskegee experiments, was associated with COVID-19 vaccine hesitancy among Black Americans. Another small study among Black undergraduates described these historical examples of racist and unethical practices as “backdrops” that informed their contemporary perceptions of how Black people continue to be discounted (24).

Additionally, present day manifestations of structural racism with historical antecedents, for example, the downstream impact of redlining, perpetuate healthcare access and socioeconomic disparities (25,26) that subsequently are likely to influence vaccine-related decisions. For various geographic and socioeconomic reasons, Black people are less likely to have access to a primary care physician (27–29) and more likely to use emergency care, a relationship partly mediated by mistrust in the healthcare system (28). Therefore, while primary care physicians are often cited across different racial and ethnic groups as the most trusted person when it comes to vaccine decision-making (30,31), structural inequalities in healthcare access means that many marginalized communities lack access to these heavily trusted sources (32,33). Some population-based studies support the link between access to primary care and those provider recommending vaccines to higher rates of COVID-19 vaccine uptake (34,35).

As alluded to above, the lived experience of historical racism, present day racism, including structural manifestations of it, and medical mistrust are intertwined. For example, one qualitative study linked historical mistrust to the way Black people in the Deep South have been treated by the healthcare system as a factor contributing to vaccine hesitancy (36). Another focus group study with Black and Latinx community members also identified "pervasive mistreatment" as a basis for vaccine hesitancy in those communities (37), suggesting that it is difficult to separate perceptions of medical racism from institutional mistrust in healthcare. Similarly, an analysis of online posts found that mistrust of vaccines and the motivations of official institutions (i.e. institutional mistrust) were commonly expressed in online platforms viewed by the Black community (38). Similar concerns were heard in focus groups of Black salon and barbershop owners (39) and in a study focusing on older Black and Hispanic adults (40). In addition to these fears, Bateman and colleagues conducted virtual focus groups and identified mistrust of the COVID-19 vaccine development process among Black and Latinx participants from the Deep South (36). Similarly, while conceptually separated here, concerns

about vaccine safety are also likely intermixed with institutional mistrust and experiences with racism since safety is assessed by government agencies. For example, a survey of people in underserved communities in North Carolina identified safety concerns and government mistrust as the most important factors for vaccine hesitancy among Black and Latinx respondents (41).

The purpose of this study was to probe more deeply into these factors to get a better understanding of the complexities involved in lower COVID-19 vaccine uptake among Black and Latinx people. To do this, we conducted two separate online bulletin boards, one with participants from each community who self-identified as vaccine hesitant, to probe the factors behind their vaccine decisions. We were interested in understanding what motivates people in the Black and Latinx communities to be COVID-19 vaccine hesitant. Originally, we intended this as two separate inquiries, one involving Black and the other involving Latinx participants and therefore the designs and recruitment strategies of the online bulletin boards differed. However, we observed remarkably similar responses from participants in the two groups and therefore decided to combine them into a single report.

These bulletin boards were conducted within the first few months after COVID-19 vaccines were made available and reflect attitudes at that time. Gaps in COVID-19 vaccination rates among racial and ethnic groups have since narrowed and attitudes about vaccines may have shifted. However, the results of these bulletin boards remain important for two reasons: first, because they provide insight into important drivers of vaccine hesitancy in Black and Latinx communities; and second, because they may help inform strategies to support future vaccine demand as new healthcare challenges inevitably arise.

Methods

We conducted two bulletin boards from July 13 to 22, 2021, following the COVID-19 vaccine rollout in the U.S. Informed consent via an online form was obtained from each participant prior to the start of the study, and they were assured that participation was voluntary.

For peer review only - http://bmjopen.bmj.com/site/about/guidelines.xhtml

Participants were told that they could end their participation at any time and were free to leave any questions unanswered. Subjects were paid $120 for their participation. This research was deemed exempt from IRB review by Ethical and Independent Review Services and approved by the Weill Cornell IRB. Due to privacy concerns, data are not currently publicly available, but de-identitified data can be obtained by researchers on a case-by-case basis by contacting the authors.

A bulletin board is an asynchronous online discussion involving greater numbers of individuals than typical focus groups and taking place over an extended period (42–44). Participants log into a password-protected site run by an external third party (QualBoard, since acquired by Sago) that creates such dedicated platforms to answer questions that are posted and monitored by a moderator. The moderator can also follow up on responses for clarification or elaboration. The bulletin board is a flexible research tool that allows the moderator to post questions and probe any individual participant following their entry. The respondents can take as much time to respond as they need. Individual responses are initially uninfluenced by the group, as participants do not see other responses to any given question until they have posted their own response. This method helps to minimize the social desirability bias (45) that may influence participants after exposure to another's responses.

Data collection. Participants in the bulletin board with Black participants were recruited from a panel of people who have previously agreed to participate in online surveys. They were contacted by email with an invitation to participate. If interested, they were asked to respond to an online screener that assessed their level of vaccine hesitancy. People who had already been vaccinated, intended to be vaccinated soon, or who adamantly opposed COVID-19 vaccination were excluded. For recruiting purposes, we defined vaccine hesitancy as adults who were not categorically opposed to vaccines, but were undecided as to whether it was safe to receive the new COVID-19 vaccines. We then screened for individuals that met these criteria. If qualified,

For peer review only

they provided their contact information and were given instructions for logging into the bulletin board. Please see Table 1 for demgraphics of the participants.

The process for recruiting participants from Latinx communities differed from that for Black participants. We posted invitations in Spanish on various Facebook pages created for Latinx sub-populations, such as groups for communities from Peru, Colombia, Mexico and the Dominican Republic. If interested, they were asked to complete the online screening questionnaire in Spanish, to determine if they met the criteria for participation, which were the same as for the participants in the Black groups as described above. If qualified, they provided their contact information, and were supplied with instructions for logging into the bulletin board. This project was designed to inform subsequent interventions to address vaccine hesitancy. Therefore, the demographics of each group were chosen to approximately match those of the groups in which interventions would take place in a later study based on observational assessment of such spaces by our interventionists (for more info on these interventions, see (46,47). Similarly, our funding for this project came from a source exclusively prioritizing health in the United States, we focused on finding participants that were relatively representative of these racial and ethnic groups living in the US according to US Census American Community Survey 5-year estimates.

When participants logged into the bulletin board, they were presented with an introduction from the moderator, a review of the process, and a reminder that they were not obliged to answer any question. They were reassured that the research was anonymous and their identities, including contact information, would not be shared. The moderators of the bulletin boards introduced themselves at the outset and posted their photographs so that the respondents could see them. Participants were allowed to post photographs of themselves to the group, though this was not required.

Participants were then presented with the first of a series of questions. Only after a participant entered their response to a question were they able to see how other participants

responded to that same question. At this point, they were free to respond to what other participants had said. After responding to all of the questions posted for that day, they were reminded to check back periodically to respond to possible follow-up questions posted by the moderator. This process continued over three days, with a different set of questions posted each day.

Bulletin board questions were designed for flexible, open-ended inquiry. The research did not seek to confirm any hypotheses but rather to explore the range of perceptions and attitudes that exist in the vaccine hesitant population and to identify important influencers of those perceptions and attitudes, including trusted sources of information, media outlets, social networks, community leaders, health professionals, etc. Examples of the various topics of inquiry and discussion can be found in Table 2 and the facilitators guide included in supplementary material. We also asked participants' perspectives on influenza vaccines, but only data from questions about COVID-19 vaccines are included here for the following reasons. First, we found participants more expressive about COVID-19 vaccines and terse in their feelings about influenza vaccination by comparison. Our resulting analysis of influenza discussions quickly reached thematic saturation, with less range of sentiment than what has been reported elsewhere in the literature (48,49). Attempts to probe this lack of interest/enthusiasm in influenza vaccination were unsuccessful.

Data quality control. The study employed purposive sampling with screening to ensure that respondents reflected the target population in terms of attitudinal, behavioral, and demographic characteristics. The sample was highly diverse with respect to age, geography, socio-economic status, and in the case of the Latinx sample, with respect to both level of acculturation to the U.S. and national heritage (see Table 1).

The bulletin boards were conducted by trained moderators, each with 20+ years of qualitative research experience. The Latinx bulletin boards were conducted in Spanish by a

Latinx moderator; the Black bulletin boards were conducted in English by a Black moderator. The Spanish-language discussion among Latinx respondents was translated into English by an automated translation program provided by the online platform. This was done for the benefit of those observing the discussion who were not Spanish speakers. The automated translation was not used, however, for the purposes of reporting due to some translation errors. Transcripts included in the report were translated by professional Spanish-speaking moderators.

Methods of analysis. A combination of methods was employed in the analysis of the content generated by these bulletin boards, including interpretive phenomenological analysis (IPA), narrative analysis, and, secondarily, qualitative content analysis. Taking a reflexive, phenomenological constructivist approach to understand participant viewpoints, these methods enabled us to explore how respondents narrate and make sense of their prior experiences with vaccines, with medical professionals, and with various sources of medical and health-related information. They also enabled us to observe how participants rationalize their hesitancy with respect to COVID-19 vaccination, and to identify a range of social, emotional, and perceptual barriers to vaccination. Analysis enabled us to identify the range of opinions exhibited, opinions that are universally shared and those that are more idiosyncratic and portray how different perceptions tend to be clustered or coupled.

Initial coding was done manually by WS. Transcripts were read through once in their entirety by prior to coding. On a second readthrough, relevant text was highlighted, codes were inductively drawn out, and labeled in text margins. Codes were then aggregated and organized into themes which were discussed in meetings with co-authors discussed in more detail below. Themes were then arranged with key quotations pulled out as illustrative examples.

Data credibility was assessed through discursive triangulation. Initial coding was followed by a process of layered discussions. Specifically, that meant multiple meetings between the primary data analyst and a supervisor to process codes and themes, followed by

For peer review only - http://bmjopen.bmj.com/site/about/guidelines.xhtml

further coding and thematic discussions and revisions with the first and senior author. After consensus on themes had been reached by those four authors, subsequent review and discussion then took place with the remaining co-authors.

Interpretive phenomenological analysis (IPA). Data collection was not designed to test hypotheses or preconceptions, nor was data analysis. The intent was to use the data gathered to better understand the experiential world of the respondents, how they understand the phenomenon of the ongoing pandemic, and how they rationalize their decision to refrain from vaccination (50). Through this bottom-up analysis, we sought commonalities and patterns in experiences and shared forms of reasoning to inform a richer understanding of vaccine hesitancy. In addition, the analysis included any consistent variations in participants' responses that corresponded with major demographic variables such as gender and age. For the descriptive analysis, we identified and cataloged the fullest possible range of opinions around vaccine hesitancy, including commonly cited sources of information, facts, anecdotes, and trusted sources of information, regarding the pandemic and COVID-19 vaccines.

Narrative analysis. In addition to identifying and cataloging the range of opinions and perceptions articulated by participants, the analysis focused on identifying the ways in which information has been woven into narratives. This analysis focused on participants' descriptions of their experiences during the pandemic, their methods for searching for and processing relevant information, and the stories they tell themselves about the need or lack of need for a vaccine. In addition, we analyzed the trajectory of each individual participant's experience with vaccines, looking to identify key moments when their attitudes reportedly changed. This analysis also sought to identify pre-existing narratives and how those intersect with participants' narratives about the pandemic, such as mistreatment of marginalized populations by the healthcare system and lack of trust in the government. This analysis also attempted to gauge

For peer review only

the extent to which participants' narratives are fixed, are still being formed, or remain open to revision.

As an additional check on our data and to ensure we met the objective of the study, we examined certain topics of high concern (historical racism, present-day structural racism, and medical mistrust) using a directed approach in secondary content analysis (51).

Patient and Public Involvement

None.

Results

We conducted one bulletin board with 30 people from the Black community and one with 30 people from the Latinx community. Characteristics of the participants can be found in Table 1. The themes obtained from the bulletin boards about COVID-19 vaccines in both the Black and Latinx groups were remarkably similar and therefore we combined them in this section. The analysis suggests several interrelated barriers to COVID-19 vaccination are at work in both Black and Latinx communities, strongly influencing vaccine behaviors in these populations. Five main themes and several sub-themes emerged. Illustrative quotations can be found in Tables 3, 4, and 5:

1. **Safety concerns (Table 3)**

- Vaccine unknowns. Vaccines are a "black box." Some participants perceived vaccine ingredients to be elusive or intentionally obscured with mysterious ingredients.
- Fears about COVID-19 vaccine safety. Participants expressed many fears and doubts regarding both the short- and long-term safety of the vaccines; even those who express high trust in doctors and science and low trust in social media still say stories of vaccine-induced illness make them highly uncertain.

For peer review only

- Conviction that the COVID-19 vaccine can kill you. Some participants believe that the vaccine is directly responsible for deaths.
- Concerns about scientific uncertainty. Public scientific debates about vaccine safety and adverse side effects instill and perpetuate doubts by creating the appearance of scientific uncertainty even among those who normally trust medical professionals. Many seem to almost throw up their hands and say, “I can’t decide what’s true and what’s not, so best to do nothing,” or to wait for more conclusive information.

2. Skepticism about vaccine efficacy (Table 3)

- COVID-19 vaccines are not effective. Several stories about new variants, breakthrough infections, and surging cases suggested a belief that the vaccine would not be effective in protecting them.
- COVID-19 vaccines are insufficient. Even with an effective vaccine, mass vaccination is not enough to return life to normal and that COVID-19 is here to stay, implying that the vaccine’s benefits may be exaggerated.
- COVID-19 vaccines do not prevent transmission. Although vaccines reduce the risk of transmission, news that people can still pass COVID-19 on to others even after being vaccinated is conflated with a narrative that vaccines do not work as intended, thus undermining the argument for getting it to protect others.

3. Risk/benefit calculations were not perceived to favor vaccines (Table 4)

- Vaccines are riskier than the virus. Participants frequently assigned greater risk to the vaccine than to the virus itself and noted that there are other ways to prevent infection (like masking), so on balance the vaccines are felt to be unnecessary.

For peer review only

- COVID-19 vaccines are not necessary. Participants in both groups often believed they were not at risk of dying from COVID-19; they believed they could contract the virus and recover from it. They also believed that any illness would be mild, underscoring a lack of urgency to be vaccinated.
- COVID-19 vaccines are only for the most vulnerable. Vaccines are for the most vulnerable, such as older people and immunocompromised people, not the young and healthy, or those being careful and taking other precautions.

4. Limited trust in institutions (Table 5)

- Limited trust in physicians. Many say that they trust their primary care doctors the most when it comes to their health, but that trust does not always extend to advice about the COVID-19 vaccines; they do not necessarily see their doctors as experts in this regard. For instance, some seem to say, "at this point, no one can claim to be an expert on these vaccines. So, no one can truly tell me what is best."
- Lack of trust in government. There is a lack of trust in government in general and especially in government spokespersons, undermining their authority as credible messengers. Many tune them out or do not lend them credence, even those who otherwise trust their doctors and medical professionals. Some people suggest that the very fact that the government so badly wants them to get a vaccine makes them not want to get it.
- Limited trust in public health authorities. While some of the vaccine hesitant respondents expressed very high regard for medical professionals and for public health authorities in general, they were more critical of public agency-relayed information about COVID-19 vaccines. Some argued that public health authorities only say what they are told to say

For peer review only

by the administration. Some participants mistrust the CDC, largely because they viewed the agency as frequently changing its advice and guidelines.

5. How health outcomes differ by race and/or ethnicity (Table 5)

Participants in both groups perceived structural racism as factors that influence a group's risk of infection and the likelihood of having access to vaccines. However, the participants did not cite structural issues as influencing either their own personal risk of infection or their decision to be vaccinated. Two people mentioned the Tuskegee experiments (52) as indicative of abuses against Black people by the healthcare system and a reason to be wary of healthcare system programs, including COVID-19 vaccines. The potential benefits of vaccination, such as protecting vulnerable communities, were not raised as a motivation for vaccination.

Other observations. Although themes and sub-themes about vaccine hesitancy were quite similar between the Black and Latinx groups in this study, there was one notable difference in where participants noted obtaining health information. Black participants were more likely to emphasize obtaining information about COVID-19 vaccines from the internet, despite having what appears to be strong relationships with medical providers. Latinx participants also had strong relationships with and trust in medical providers and seemed to make less use of the internet for health information. Both groups rely heavily on trusted friends and relatives for health information. Even with that support, however, moving to vaccine acceptance for some people can be very difficult and take more time than for others. As one Black participant noted: "*I love and trust my family; I love and trust my pastor. And they all made their position known. And I know none of them do things haphazardly… [but] the jury is still out for me… I'm just straddling the fence, and it's just a personal thing with me… They've all endorsed it, my pastor endorsed it…but I'm just not there, I'm not."*

For peer review only

Aside from the difference noted above, we did not observe distinct themes specific to any particular sub-group (e.g. by country of origin). Additionally, it should be noted that participants did not endorse conspiracy theories or unsubstantiated notions about vaccines (e.g., that they contain microchips) that have been voiced in anti-vaccine channels.

Discussion

The results of this research suggest that interrelated barriers to vaccination are at work in communities of color and strongly influence COVID-19 vaccine behaviors in these communities. Two main sets of concerns emerged from in these bulletin boards: that the vaccines are unsafe and that they are insufficiently effective. These concerns are remarkably similar to those observed in an earlier bulletin board study that involved a group of participants that had a majority of white people (53). Indeed, these may be ubiquitous influences on vaccine hesitancy across racial, ethnic, and national groups (54–56).

Several participants seemed eager to make clear they felt race and ethnicity were factors in community viral infection susceptibility because of the history of structural racism in healthcare and medicine. Lack of access to healthcare and to vaccination sites has been found to be a factor in limiting vaccination among Black and Latinx people (37). The history of racism and medical experimentation on people of color in the United States was cited as among the reasons for vaccine skepticism among Black participants in one recent study (57). However, as in the survey study of Martin, Stanton, and Johnson (23), participants in our study did not frequently express a conviction that historical racism was a factor in their *personal* decisions about vaccination. Two of the 30 participants in our Black participant group directly named the Tuskegee experiments, a hallmark of unethical, racist scientific and healthcare practices in the U.S. Thus, although participants in both groups often cited examples of structural racism in general, they were more likely to express individual feelings of fear and skepticism about the vaccines as the main factors in making them hesitant to be vaccinated.

For peer review only

Historical traumas like the experiments that took place in Tuskegee may still have an effect on people's attitudes and decision making even if not explicit (58,59). Current experiences with racism such as health outcome disparities may be as or even more important in shaping ways that people of color make decisions about healthcare issues like vaccination (60). It is possible that the way we framed questions in these bulletin boards influenced participants toward speaking more about their individual concerns as the main factors in COVID-19 vaccine decision making and away from broader discussions about the impact of historical and present racism on those decisions. It is also possible that for these participants at least, while recognizing that structural factors like crowded work conditions and lack of healthcare access make communities of color more likely to acquire COVID-19 and to have more negative outcomes, individual fears and skepticism about the COVID-19 vaccines were indeed the most pressing concerns that influenced vaccine hesitancy.

Although participants expressed trust in their own personal healthcare providers, they exhibitied a general lack of trust in agencies and institutions that are charged with the responsibility of informing and reassuring the public about vaccine safety and efficacy. This is not a unique finding. Previous studies have found people in Black and Latinx communities have low levels of trust in the healthcare system (27), and racial differences in healthcare access has been noted as a contributing factor (28).

This study also raises key questions about the information environments participants were immersed in. In his Special Advisory on misinformation in 2021, the US Surgeon General discussed the need to build a healthy "information environment" (61). While a worthy endeavor, there are currently no standard ways to measure whether individual or community is immersed in a healthy information environment, defined as where people and communities are immersed in high-quality information of public health importance and enveloped by a communication context that underscores the trustworthiness and importance of that quality (62). It is important to note that, at the time the bulletin boards were being done, media coverage of about the

For peer review only

vaccines contained a mixture of concerning and reassuring information, first about breakthrough infections, viral variants, and prominent reporting on rare vaccine adverse side effects, and second, describing a public health and scientific consensus that that the vaccines were safe and they were highly effective at preventing morbidity and mortality due to COVID-19.

Yet quotations from participants generally reflected more of the concerns than reassuring information, providing some insight into what their information environment may consist of. Specifically, while it was clear that participants saw and heard abundant information about vaccines, much of that information did not appear to accurately present information about both individual risks and collective benefits. Concerningly, suggests that the concerns that were circulating in these communities then met most definitions of scientific misinformation (63,64). An alternative explanation is that despite the presentation of high-quality information, misinformation or worrisome information about the vaccines could have been more effective at shaping vaccine-related feelings and decisions. In either case, the implications are concerning. Therefore, it seems unlikely that merely supplying more facts about vaccine safety and efficacy will be sufficient on its own to sufficiently modify that information environment and change participants' views on obtaining vaccines.

Some key points that public health professionals may consider when contemplating how to encourage vaccine uptake in their work with marginalized communities include:

- Leveraging trusted sources to challenge narratives of safety and inefficacy by emphasizing personal and communal benefits over risk (e.g. to protect one from new strains or to protect loved ones, for example). While previous studies of HPV vaccines observed Black people did not leverage family and friends for information (65,66), our data suggest this may differ by vaccine. Given how both Black and Latinx participants noted obtaining much of their health information from family and friends, encouraging those who have been vaccinated to reassure unvaccinated family members and friends

For peer review

about safety may be an efficient way to disrupt these narratives in these priority communities. This approach would be consistent with others' recommendations to strengthen pro-vaccine messages to leverage non-medical, in-group spokespeople to share community benefits of vaccines with Black communities (67).

- Explicitly and more emphatically framing both risks of COVID-19 and benefits of vaccination in community-level terms. It is unclear why participants often offered narratives of racial discrimination at structural and community levels yet narratives about information gathering, the negative effects of COVID-19 and benefits from vaccination were not conceptualized in a similar light. This suggests both the success of current messaging on issues of structural racism, and the insufficiency of public health messaging to penetrate dominant media narratives framing aspects of COVID-19 in primarily individualistic terms (68). In this way, the data presented here reflect concerns stated elsewhere about the "individualization of pandemic control" (69,70).

- As many participants noted institutional mistrust in government and the health system, it it will be essential for public health professionals to leverage partnerships to effectively reach marginalized community members with trusted messengers. This includes improving access to vaccines for people in traditionally underserved communities as access issues can foment mistrust and suspicion of the healthcare system and efforts to ameliorate them may make some people more likely to accept vaccines (71). Access must also be coupled with sufficient training to primary care clinicians to build trust with Black and Latinx communities. As many participants noted that they did not consider their physician sufficiently expert to trust their opinion on vaccines, counteracting misinformed ideas held about vaccines by patients will only be successful if there is sufficient trust in the relative expertise of those care providers.

For peer review only

Limitations

This study has several limitations, including inherent self-selection bias in the sample of participants. There is also inevitable bias toward the views of those comfortable sharing their opinions in a group discussion with others in a digital setting where social desirability bias may make some participants reticent to share what may be perceived as outlandish opinions. This may have been a factor in the fact that subjects did not, for the most part, mention historically racist events and the United States' racialized history does not rule out that these are important factors for vaccine hesitancy. We did not ask specific questions about these issues. Participants' reports of mistrust of public health authorities and the government represent the result of both historical racism and personal experiences of racism. Thus, while we can report our observation that for the most part neither people in our Black nor Latinx groups volunteered racism as affecting their own vaccine decisions, deeper probing might have elicited that as an important factor. Indeed, Dong and colleagues conducted semi-structured interviews with 24 Black Americans and reported that, "systemic racism was discussed as the root cause of the different types of mistrust" (72). Finally, while we initially asked questions about influenza, these discussions yielded surprisingly narrow themes. We suspect but cannot confirm that this observation was connected to of the context and timing of our study as data was collected in the first months after the release of COVID-19 vaccines.

In summary, bulletin boards with COVID-19 vaccine-hesitant people from the Black and Latinx communities revealed that the major factors influencing vaccine hesitancy involve fears of lack of safety and efficacy of the vaccines. There is a misperception that not being vaccinated is a social norm because of media emphasis on unvaccinated people. These attitudes are reinforced by a perception of lack of consensus about the vaccines among experts, mistrust of government officials and institutions, and belief that other measures are sufficient to prevent

For peer review only

acquisition and spread of COVID-19. Future research will focus on strategies to improve vaccine acceptance that do not rely only on providing facts but account also for the anxieties and fears that motivate vaccine hesitancy.

For peer review only

Contributorship Statement

DS and JMG conceived of the study, wrote the protocol, obtained funding and engaged WS and NG to carry out the research and conduct preliminary data analysis. DS, SG, JMG, WS and NG were responsible for inclusion/exclusion criteria of both bulletin board groups. WS and NG were responsible for bulletin board design, and recruitment of participants. WG undertook initial interpretive phenomenological and narrative analysis. SG, SW, LH, MR, AA contributed to further data review and interpretation with DS and JMG. All authors read and critically evaluated multiple drafts of the manuscript before providing final approval of the version to be published.

Competing Interests

The authors state they have no competing interest to declare.

Funding

This work was supported by the Robert Wood Johnson Foundation (grant numbers 76935 and 78084) and Weill Cornell Medicine's JumpStart program (Grant number N/A). The opinions expressed in this piece do not necessarily reflect those of either the Robert Wood Johnson Foundation or Weill Cornell Medicine.

Data Sharing Statement

Due to privacy concerns, data are not currently publicly available, but de-identitified data can be obtained by researchers on a case-by-case basis by contacting the authors. Patients were not involved in the design of the study.

Ethics Approval Statement

This research was deemed exempt from IRB review by Ethical and Independent Review Services and approved by the Weill Cornell IRB (19-10020908).

Acknowledgments

This work was supported by the Robert Wood Johnson Foundation (grant numbers 76935 and 78084) and Weill Cornell Medicine's JumpStart program (grant number N/A). The opinions expressed in this piece do not necessarily reflect those of either the Robert Wood Johnson Foundation or Weill Cornell Medicine. We also thank the peer reviewers for their thoughtful and insightful comments

Conflicts of Interest

The authors state that they have no conflicts of interest to declare.

For peer review only

References

1. Pingali C, Meghani M, Razzaghi H, Lamias MJ, Weintraub E, Kenigsberg TA, et al. COVID-19 Vaccination Coverage Among Insured Persons Aged ≥16 Years, by Race/Ethnicity and Other Selected Characteristics - Eight Integrated Health Care Organizations, United States, December 14, 2020-May 15, 2021. MMWR Morb Mortal Wkly Rep. 2021 Jul 16;70(28):985–90.

2. Szilagyi PG, Thomas K, Shah MD, Vizueta N, Cui Y, Vangala S, et al. Likelihood of COVID-19 vaccination by subgroups across the US: post-election trends and disparities. Hum Vaccin Immunother. 2021 Oct 3;17(10):3262–7.

3. Kriss JL, Hung M-C, Srivastav A, Black CL, Lindley MC, Lee JT, et al. COVID-19 Vaccination Coverage, by Race and Ethnicity - National Immunization Survey Adult COVID Module, United States, December 2020-November 2021. MMWR Morb Mortal Wkly Rep. 2022 Jun 10;71(23):757–63.

4. Magesh S, John D, Li WT, Li Y, Mattingly-App A, Jain S, et al. Disparities in COVID-19 Outcomes by Race, Ethnicity, and Socioeconomic Status: A Systematic-Review and Meta-analysis. JAMA Netw Open. 2021 Nov 1;4(11):e2134147.

5. Agarwal R, Dugas M, Ramaprasad J, Luo J, Li G, Gao GG. Socioeconomic privilege and political ideology are associated with racial disparity in COVID-19 vaccination. Proc Natl Acad Sci U S A [Internet]. 2021 Aug 17;118(33). Available from: http://dx.doi.org/10.1073/pnas.2107873118

6. Njoku A, Joseph M, Felix R. Changing the Narrative: Structural Barriers and Racial and Ethnic Inequities in COVID-19 Vaccination. Int J Environ Res Public Health [Internet]. 2021 Sep 20;18(18). Available from: http://dx.doi.org/10.3390/ijerph18189904

7. Bacong AM, Haro-Ramos AY. Willingness to Receive the COVID-19 Vaccine in California: Disparities by Race and Citizenship Status. J Racial Ethn Health Disparities. 2022 Nov 30;1–10.

8. Larson HJ, Jarrett C, Schulz WS, Chaudhuri M, Zhou Y, Dube E, et al. Measuring vaccine hesitancy: The development of a survey tool. Vaccine. 2015 Aug 14;33(34):4165–75.

9. Nguyen LH, Joshi AD, Drew DA, Merino J, Ma W, Lo C-H, et al. Self-reported COVID-19 vaccine hesitancy and uptake among participants from different racial and ethnic groups in the United States and United Kingdom. Nat Commun. 2022 Feb 1;13(1):636.

10. Saluja S, Nok Lam C, Wishart D, McMorris A, Cousineau MR, Kaplan CM. Disparities in COVID-19 vaccine hesitancy among Los Angeles County adults after vaccine authorization. Prev Med Rep. 2021 Dec;24:101544.

11. Piltch-Loeb R, Savoia E, Goldberg B, Hughes B, Verhey T, Kayyem J, et al. Examining the effect of information channel on COVID-19 vaccine acceptance. PLoS One. 2021 May 12;16(5):e0251095.

For peer review only

12. Khubchandani J, Macias Y. COVID-19 vaccination hesitancy in Hispanics and African-Americans: A review and recommendations for practice. Brain Behav Immun Health. 2021 Aug;15:100277.

13. Momplaisir FM, Kuter BJ, Ghadimi F, Browne S, Nkwihoreze H, Feemster KA, et al. Racial/Ethnic Differences in COVID-19 Vaccine Hesitancy Among Health Care Workers in 2 Large Academic Hospitals. JAMA Netw Open. 2021 Aug 2;4(8):e2121931.

14. Allen JD, Abuelezam NN, Rose R, Fontenot HB. Factors associated with the intention to obtain a COVID-19 vaccine among a racially/ethnically diverse sample of women in the USA. Transl Behav Med. 2021 Apr 7;11(3):785–92.

15. Thompson HS, Manning M, Mitchell J, Kim S, Harper FWK, Cresswell S, et al. Factors Associated With Racial/Ethnic Group–Based Medical Mistrust and Perspectives on COVID-19 Vaccine Trial Participation and Vaccine Uptake in the US. JAMA Netw Open. 2021 May 3;4(5):e2111629–e2111629.

16. Liu R, Li GM. Hesitancy in the time of coronavirus: Temporal, spatial, and sociodemographic variations in COVID-19 vaccine hesitancy. SSM Popul Health. 2021 Sep;15:100896.

17. Longoria, Acosta, Urbani, Smith. A Limiting Lens: How Vaccine Misinformation Has Influenced Hispanic Conversations Online [Internet]. First Draft; 2021 Dec. Available from: https://firstdraftnews.org/wp-content/uploads/2021/12/COVID-19_VACCINE_MISINFORMATION_HISPANIC_COMMUNITIES.pdf

18. Morales DX, Paat Y-F. Hesitancy or Resistance? Differential Changes in COVID-19 Vaccination Intention Between Black and White Americans. J Racial Ethn Health Disparities. 2022 Dec 22;1–13.

19. Skloot R. The Immortal Life of Henrietta Lacks. Crown Publishing Group, a division of Random House, Incorporated; 2017. 381 p.

20. Reverby SM. Examining Tuskegee: The Infamous Syphilis Study and Its Legacy. Univ of North Carolina Press; 2009. 416 p.

21. Gamble VN. A legacy of distrust: African Americans and medical research. Am J Prev Med. 1993 Nov-Dec;9(6 Suppl):35–8.

22. Williams DR, Mohammed SA. Racism and Health I: Pathways and Scientific Evidence. Am Behav Sci [Internet]. 2013 Aug 1;57(8). Available from: http://dx.doi.org/10.1177/0002764213487340

23. Martin KJ, Stanton AL, Johnson KL. Current health care experiences, medical trust, and COVID-19 vaccination intention and uptake in Black and White Americans. Health Psychol [Internet]. 2022 Oct 13; Available from: http://dx.doi.org/10.1037/hea0001240

24. Soboti JM. “If You are Going to Step on This Campus, You Have to Get Vaccinated”: A Qualitative Understanding of COVID-19 Vaccine Hesitancy Among Black Emerging Adults Returning to College Campuses. Emerging Adulthood. 2023 Jun 1;11(3):735–47.

For peer review only

25. King CJ, Buckley BO, Maheshwari R, Griffith DM. Race, place, and structural racism: A review of health and history in Washington, D.c. Health Aff . 2022 Feb;41(2):273–80.

26. Egede LE, Walker RJ, Campbell JA, Linde S, Hawks LC, Burgess KM. Modern Day Consequences of Historic Redlining: Finding a Path Forward. J Gen Intern Med. 2023 May;38(6):1534–7.

27. Gaskin DJ, Dinwiddie GY, Chan KS, McCleary RR. Residential segregation and the availability of primary care physicians. Health Serv Res. 2012 Dec;47(6):2353–76.

28. Arnett MJ, Thorpe RJ Jr, Gaskin DJ, Bowie JV, LaVeist TA. Race, Medical Mistrust, and Segregation in Primary Care as Usual Source of Care: Findings from the Exploring Health Disparities in Integrated Communities Study. J Urban Health. 2016 Jun;93(3):456–67.

29. Brown EJ, Polsky D, Barbu CM, Seymour JW, Grande D. Racial Disparities In Geographic Access To Primary Care In Philadelphia. Health Aff . 2016 Aug 1;35(8):1374–81.

30. Oh NL, Biddell CB, Rhodes BE, Brewer NT. Provider communication and HPV vaccine uptake: A meta-analysis and systematic review. Prev Med. 2021 Jul;148:106554.

31. Opel DJ, Mangione-Smith R, Robinson JD, Heritage J, DeVere V, Salas HS, et al. The Influence of Provider Communication Behaviors on Parental Vaccine Acceptance and Visit Experience. Am J Public Health. 2015 Oct;105(10):1998–2004.

32. Benkert R, Cuevas A, Thompson HS, Dove-Meadows E, Knuckles D. Ubiquitous Yet Unclear: A Systematic Review of Medical Mistrust. Behav Med. 2019 Apr-Jun;45(2):86–101.

33. Siegel M, Critchfield-Jain I, Boykin M, Owens A, Muratore R, Nunn T, et al. Racial/Ethnic Disparities in State-Level COVID-19 Vaccination Rates and Their Association with Structural Racism. J Racial Ethn Health Disparities. 2022 Dec;9(6):2361–74.

34. Nguyen KH, Yankey D, Lu P-J, Kriss JL, Brewer NT, Razzaghi H, et al. Report of Health Care Provider Recommendation for COVID-19 Vaccination Among Adults, by Recipient COVID-19 Vaccination Status and Attitudes - United States, April-September 2021. MMWR Morb Mortal Wkly Rep. 2021 Dec 17;70(50):1723–30.

35. Lo C-H, Chiu L, Qian A, Khan MZ, Alhassan HA, Duval AJ, et al. Association of Primary Care Physicians Per Capita With COVID-19 Vaccination Rates Among US Counties. JAMA Netw Open. 2022 Feb 1;5(2):e2147920.

36. Bateman LB, Hall AG, Anderson WA, Cherrington AL, Helova A, Judd S, et al. Exploring COVID-19 vaccine hesitancy among stakeholders in African American and Latinx communities in the deep south through the lens of the Health Belief Model. Am J Health Promot. 2022 Feb;36(2):288–95.

37. Balasuriya L, Santilli A, Morone J, Ainooson J, Roy B, Njoku A, et al. COVID-19 Vaccine Acceptance and Access Among Black and Latinx Communities. JAMA Netw Open. 2021 Oct 1;4(10):e2128575.

38. Dodson K, Mason J, Smith R. Covid-19 vaccine misinformation and narratives surrounding Black communities on social media October 13, 2021 [Internet]. First Draft News; 2021. Available from: https://firstdraftnews.org/long-form-article/covid-19-vaccine-misinformation-black-communities/

39. Momplaisir F, Haynes N, Nkwihoreze H, Nelson M, Werner RM, Jemmott J. Understanding Drivers of COVID-19 Vaccine Hesitancy Among Blacks. Clin Infect Dis [Internet]. 2021 Feb 9; Available from: http://dx.doi.org/10.1093/cid/ciab102

40. Kricorian K, Turner K. Covid-19 vaccine reluctance in older black and hispanic adults: Cultural sensitivity and institutional trust. J Am Geriatr Soc. 2021 Apr;69(S1):S214–S214.

41. Doherty IA, Pilkington W, Brown L, Billings V, Hoffler U, Paulin L, et al. COVID-19 vaccine hesitancy in underserved communities of North Carolina. PLoS One. 2021 Nov 1;16(11):e0248542.

42. Stancanelli J. Conducting an Online Focus Group. The Qualitative Report; Fort Lauderdale. 2010 May;15(3):761–5.

43. Turney L, Pocknee C. Virtual focus groups: New technologies, new opportunities, new learning environments [Internet]. ascilite.org; 2004 [cited 2022 Dec 19]. Available from: https://www.ascilite.org/conferences/perth04/procs/pdf/turney.pdf

44. Gordon AR, Calzo JP, Eiduson R, Sharp K, Silverstein S, Lopez E, et al. Asynchronous Online Focus Groups for Health Research: Case Study and Lessons Learned. International Journal of Qualitative Methods. 2021 Jan 1;20:1609406921990489.

45. Latkin CA, Edwards C, Davey-Rothwell MA, Tobin KE. The relationship between social desirability bias and self-reports of health, substance use, and social network factors among urban substance users in Baltimore, Maryland. Addict Behav. 2017 Oct;73:133–6.

46. Gorman JM, Scales DA. Leveraging infodemiologists to counteract online misinformation: Experience with COVID-19 vaccines. HKS Misinfo Review [Internet]. 2022 Jan 16; Available from: https://misinforeview.hks.harvard.edu/?p=8758

47. Scales D, Gorman JM, DiCaprio P, Hurth L, Radhakrishnan M, Windham S, et al. Community-oriented Motivational Interviewing (MI): A novel framework extending MI to address COVID-19 vaccine misinformation in online social media platforms. Comput Human Behav. 2023 Apr 1;141:107609.

48. Nowak GJ, Sheedy K, Bursey K, Smith TM, Basket M. Promoting influenza vaccination: insights from a qualitative meta-analysis of 14 years of influenza-related communications research by U.S. Centers for Disease Control and Prevention (CDC). Vaccine. 2015 Jun 4;33(24):2741–56.

49. Jamison AM, Quinn SC, Freimuth VS. “You don’t trust a government vaccine”: Narratives of institutional trust and influenza vaccination among African American and white adults. Soc Sci Med. 2019 Jan;221:87–94.

For peer review only

50. Frechette J, Bitzas V, Aubry M, Kilpatrick K, Lavoie-Tremblay M. Capturing Lived Experience: Methodological Considerations for Interpretive Phenomenological Inquiry. International Journal of Qualitative Methods. 2020 Jan 1;19:1609406920907254.

51. Hsieh H-F, Shannon SE. Three approaches to qualitative content analysis. Qual Health Res. 2005 Nov;15(9):1277–88.

52. Shavers VL, Lynch CF, Burmeister LF. Knowledge of the Tuskegee study and its impact on the willingness to participate in medical research studies. J Natl Med Assoc. 2000 Dec;92(12):563–72.

53. Gorman JM, Gorman SE, Sandy W, Gregorian N, Scales DA. Implications of COVID-19 Vaccine Hesitancy: Results of Online Bulletin Board Interviews. Frontiers in Public Health [Internet]. 2022;9. Available from: https://www.frontiersin.org/article/10.3389/fpubh.2021.757283

54. Aw J, Seng JJB, Seah SSY, Low LL. COVID-19 Vaccine Hesitancy—A Scoping Review of Literature in High-Income Countries. Vaccines. 2021 Aug 13;9(8):900.

55. Rabaa J, Dorsaf H, Hend J, Sabrine O, Uta O, Yosra Z, et al. Perceptions of Tunisians on COVID-19 vaccines: A qualitative study on a sample of general population [Internet]. scivisionpub.com; 2021 [cited 2022 Dec 19]. Available from: http://scivisionpub.com/pdfs/perceptions-of-tunisians-on-covid19-vaccines-a-qualitative-study-on-a-sample-of-general-population-1843.pdf

56. Steinert JI, Sternberg H, Prince H, Fasolo B, Galizzi MM, Büthe T, et al. COVID-19 vaccine hesitancy in eight European countries: Prevalence, determinants, and heterogeneity. Sci Adv. 2022 Apr 29;8(17):eabm9825.

57. Jimenez ME, Rivera-Núñez Z, Crabtree BF, Hill D, Pellerano MB, Devance D, et al. Black and Latinx Community Perspectives on COVID-19 Mitigation Behaviors, Testing, and Vaccines. JAMA Netw Open. 2021 Jul 1;4(7):e2117074.

58. Sotero M. A Conceptual Model of Historical Trauma: Implications for Public Health Practice and Research [Internet]. Journal of health disparities research and practice. 2006 [cited 2022 Dec 19]. Available from: https://papers.ssrn.com/abstract=1350062

59. Akinyemi AA. Historical trauma compounds experiences of racial injustice. Nat Hum Behav. 2022 Sep;6(9):1183.

60. Bajaj SS, Stanford FC. Beyond Tuskegee - vaccine distrust and everyday racism. N Engl J Med. 2021 Feb 4;384(5):e12.

61. Murthy VH. Confronting Health Misinformation: The U.S. Surgeon General's Advisory on Building a Healthy Information Environment [Internet]. Office of the US Surgeon General; 2021 Jul. Available from: https://www.hhs.gov/surgeongeneral/reports-and-publications/health-misinformation/index.html

62. Scales D, Gorman J. Screening for Information Environments: A Role for Health Systems to Address the Misinformation Crisis. J Prim Care Community Health. 2022 Jan;13:21501319221087870.

For peer review only

63. Vraga EK, Bode L. Defining Misinformation and Understanding its Bounded Nature: Using Expertise and Evidence for Describing Misinformation. Political Communication. 2020 Jan 2;37(1):136–44.

64. Southwell BG, Brennen JSB, Paquin R, Boudewyns V, Zeng J. Defining and Measuring Scientific Misinformation. Ann Am Acad Pol Soc Sci. 2022 Mar 1;700(1):98–111.

65. Joseph NP, Clark JA, Bauchner H, Walsh JP, Mercilus G, Figaro J, et al. Knowledge, attitudes, and beliefs regarding HPV vaccination: ethnic and cultural differences between African-American and Haitian immigrant women. Womens Health Issues. 2012 Nov-Dec;22(6):e571-9.

66. Fu LY, Zimet GD, Latkin CA, Joseph JG. Social Networks for Human Papillomavirus Vaccine Advice Among African American Parents. J Adolesc Health. 2019 Jul;65(1):124–9.

67. Fu LY, Haimowitz R, Thompson D. Community members trusted by African American parents for vaccine advice. Hum Vaccin Immunother. 2019 Apr 11;15(7–8):1715–22.

68. Turchi J, Melton-Fant C. Media Framing of COVID-19 Racial Disparities: Lessons from Memphis, Tennessee. Sociology of Race and Ethnicity. 2022 Jul 1;8(3):355–69.

69. Tomori C, Evans DP, Ahmed A, Nair A, Meier BM. Where is the “Public” in American Public Health? Moving from individual responsibility to collective action. eClinicalMedicine [Internet]. 2022 Mar 1;45. Available from: https://doi.org/10.1016/j.eclinm.2022.101341

70. Gollust SE, Haselswerdt J. Who does COVID-19 hurt most? Perceptions of unequal impact and political implications. Soc Sci Med. 2023 Apr;323:115825.

71. Nguyen AL, Schwei RJ, Zhao Y-Q, Rathouz PJ, Jacobs EA. What Matters When It Comes to Trust in One’s Physician: Race/Ethnicity, Sociodemographic Factors, and/or Access to and Experiences with Health Care? Health Equity. 2020 Jun 29;4(1):280–9.

72. Dong L, Bogart LM, Gandhi P, Aboagye JB, Ryan S, Serwanga R, et al. A qualitative study of COVID-19 vaccine intentions and mistrust in Black Americans: Recommendations for vaccine dissemination and uptake. PLoS One. 2022 May 3;17(5):e0268020.

For peer review only

Table 1: Characteristics of bulletin board participants

	Participants from the Black community (N=30)	Participants from the Latinx community (N=30)
**Gender**		
Male	9	14
Female	21	16
**Age**		
18-29	4	13
30-39	8	8
40-49	7	6
50-59	6	3
60-69	5	0
**Marital Status**		
Married or living with partner	5	20
Divorced or widowed	6	0
Single	19	10
**Education Level**		
Less than high school	1	3
Some college	15	13
College degree	10	10
Post-graduate	4	4
**Household Income**		
Below $35,000	9	7
$35,000-$49,999	5	3
$50-$74,999	5	11
$75-$99,999	8	10
$100,000+	3	0
**Flu Vaccine History**		
Usually get the vaccine	4	3

For peer review only

Sometimes get the vaccine	12	13
Never get the vaccine	14	14
**Religion**		
Roman Catholic	NA	19
Protestant	NA	7
None	NA	3
Mormon	NA	1
**US or Foreign-Born**		
US-born	NA	17
Foreign-born	NA	13
**Heritage Country**		
Mexico	NA	8
Peru	NA	4
Ecuador	NA	4
Dominican Republic	NA	3
Venezuela	NA	2
Puerto Rico	NA	2
Colombia	NA	2
El Salvador	NA	1
Chile	NA	1
Costa Rica	NA	1
Cuba	NA	1
Guatemala	NA	1

**NA: Information not requested*

For peer review only

Table 2: Topics of inquiry and discussion on the bulletin board

Day	Topics
**1**	General health and wellbeing concerns for themselves and their families<br>Sources of health and medical information and advice<br>Primary care doctors<br>Use of trusted family and home remedies<br>Personal experiences with vaccines in the past<br>Experiences with flu vaccines<br>Awareness of messaging around vaccine safety<br>Preferred sources of information<br>Use of social media for medical or health information
**2**	Things they have heard about the COVID-19 vaccines<br>How much they trust the sources<br>What, if anything, frightens them about a COVID-19 vaccine<br>Which is more frightening to them, catching COVID-19 or getting a vaccine<br>Intentions regarding a COVID-19 vaccine<br>Perceived effectiveness of the COVID-19 vaccines<br>If and how they have discussed the vaccine with their doctors<br>If and how they have discussed the vaccine with family members or friends<br>What their community and church leaders are advising them with respect to the vaccine<br>What they have heard about vaccines and vaccine safety on social media<br>How much they trust what they see on social media<br>What public health officials are saying<br>How much trust they place in public health officials
**3**	How have their communities and their families been affected by COVID-19<br>How worried are they about possibly passing COVID-19 on to at-risk members of their families<br>How important do they feel it is to eventually receive a COVID-19 vaccine<br>How important are vaccines for restoring normalcy<br>What are the best arguments they have heard in favor of vaccination<br>How do they feel about the idea of mandated vaccination<br>What information would make them feel better about getting a COVID-19 vaccine<br>Whose endorsement of vaccination would be meaningful for them<br>Responses to various pro-vaccine messages

Table 3: Example quotations of hesitancy related to safety and efficacy concerns.

Sub-Theme	Quotations
Vaccines are a "black box"	*"I don't know what they're putting in my body."*
Fears about vaccine safety	*"Vaccines lower the fear of COVID, but not the fear of long-term effects"*<br><br>*"The unknown frightens me. What happens when the vaccine interacts with medications… what happens years from now?"*<br><br>*"What frightens me is that uncertainty. No one knows what this vaccine will do to humans long term. Let alone babies that are born after."*
Conviction that the vaccine can kill you	*"I believe I would say receiving the vaccine is most frightening. I have had several people to pass away [sic] after receiving the vaccine. Prior to the vaccine these individuals were healthy and doing fine."*<br><br>*"The idea that I could die or have health complications because of the vaccine frightens me. I've mostly read this in social media."*
Concerns about scientific uncertainty	*"There are so many conflicting reports that it is difficult to know who is being honest and factual."*
Vaccines are not effective	*"With all the reports of fully vaccinated people contracting COVID a second time I'm not convinced that the vaccine offers the protection it claims."*

For peer review only

	"From what I'm hearing it would be very effective, but some people, even though they got vaccinated ended up with COVID. I'm not really sure at the moment to be honest with you"
Vaccines are insufficient	*"Currently, only 50% effective. I have seen where there's a booster shot required every six months. I've also heard doctors and CDC state that it doesn't prevent you from getting COVID, it just lessens your likelihood the virus being as bad."*
Vaccines do not prevent transmission	*"Some people that have been vaccinated have gotten the virus. I think that it causes people to lower their guard regarding social distancing and wearing masks. New more contagious strains of the virus are still popping up."*<br><br>*"They're dying from the vaccine as well. And the vaccine is not effective. They still get the virus and pass it on to other people."*

For peer review only

Table 4: Example quotations of hesitancy related to the perception that vaccines are not worth the risk.

Sub-Theme	Quotations
Vaccines are riskier than the virus	*"I have heard the COVID virus isn't too bad and I have multiple friends that have had it. So, I guess you can say I'm more worried about the vaccine than the virus itself."*<br>*"I have already had the virus and had minimal symptoms. So I guess I could say getting the vaccine is more frightening."*<br>*"Both are scary, but getting the vaccine is more frightening for me because I feel that if I got COVID I would be fine and it wouldn't affect me much."*<br>*"At this point, me getting the COVID vaccine is more frightening [than getting COVID]. I stay by myself; I only go out if need be and I am masked up."*
Vaccines are not necessary	*"I'm not frightened at all because I take great precautions. I'm more concerned about someone passing it on to me."*
Vaccines are only for the most vulnerable	*"For me personally, I don't feel like it's necessary as I am a healthy individual with no underlying health issues, and so is my husband and child."*<br>*"I think that the vaccine is important for those who are most vulnerable. If they get sick, at least it won't be as serious."*

For peer review only

Table 5: Example quotations of hesitancy related to distrust of institutions or concerns about structural/individual racism.

Sub-Theme	Quotations
Limited trust in physicians	*"I never really trust one opinion regarding health issues. I listen to what the doctors say and suggest for any illness. Next I read all information given and search the internet for reliable sources and try to gain an understanding of the situation. At that point my decision is made."*<br><br>*"I don't view my doctor as an expert in vaccine… Kinda like having a degree in general studies vs. a specialist… I think he's knowledgeable… but don't think the level of focus and concentration points to expert."*<br><br>*"I don't think anyone is an expert. You can't know everything about such a new vaccine!"*<br><br>*"In addition to the advice of medical professionals, I also believe firmly… in the power of being natural and how people used to cure themselves in the past… The traditional remedies work."*
Lack of trust in government	*"I have a hard time trusting anything government affiliated – because they follow government directives rather than their own expertise."*<br><br>*"I don't have confidence in what the government says in general. At the end of the day they are protecting themselves and I don't believe that they are concerned about those in the lowest classes. I feel like the government if [sic] capable of lying for its own benefit."*
Limited trust in public health authorities	*"I trust most of their opinions. Not all."*<br><br>*"I trust but may not do 100% of what they say."*
Concerns about health outcomes differing by race or ethnicity	*"I'm not convinced that being Black does affect the risks of getting COVID. I know that's what reported but I'm just not convinced that it's true….. It's not the news itself that's unbelievable, it's the source. Medical institutions have subjected Black people to abuse, exploitation and experimentation since this country's foundation. It wouldn't be the first time that Black people were misled into getting vaccines with the false hope of immunity from a deadly disease."*

For peer review only

	"I feel that my community is more at risk of catching COVID due to the history of us being ignored by health professionals and the government. Additionally, we are most likely in employment opportunities that expose us to conditions that are not ideal. I don't agree that we are experiencing more serious reactions because that implies that we are unhealthy. Unhealthy behaviors are common in America and not assigned to simply one community. If we are having serious reactions, it is most likely due to our concerns being brushed aside when we seek assistance from health care workers."<br><br>*"I honestly believe that the social structure of how Black people are treated in America is more so to do with the severity of the virus to this group. Less readily available access to health care, poor living situation, less money funneled into Black community…."*<br><br>*"I don't feel like my race affects my risk of getting [COVID] but I feel like it would affect the medical care that I received if I needed medical care while I was positive."*<br><br>*"I don't think it affects people differently due to ethnicity."*<br><br>*"I don't think that race is a factor here. Anyone can get the virus."*

For peer review only

Critica Bulletin Board Discussion Guide:
Exploring Hesitancy to Vaccinate Among Latinx and African-American Communities
May 27, 2021

All questions will be partially masked (respondents can only see others' responses once they have submitted their own response). All questions will be text response (open-ended) questions unless otherwise indicated.

Introduction and Instructions:

[Multiple choice question] Welcome! My name is **Marsha**, and I will be guiding you through our online discussion over the next three days. Before we get started, please read the following information carefully and consent to participate in the discussion.

[INSERT CONSENT TEXT]

( ) Yes
( ) No [Terminate]

[Notice] Here's how the discussion will work…

Over the next three days, I will ask you questions related to the topic of vaccines. My job is to get your thoughts and opinions about this topic, and your job is to share them by typing your responses, and then submitting them. I am interested in your **honest** opinions, be they positive or negative, so please share freely and honestly throughout the discussion. There are no "right" or "wrong" answers, and you don't have to worry about your spelling or sentence structure. If you share your honest opinions and attitudes, then you will be providing the "right" answers.

For peer review only - http://bmjopen.bmj.com/site/about/guidelines.xhtml

Once you submit your response to a question, you will be able to see how the rest of the group has responded and you can "like" or comment on their posts if you have anything to add. We are all different individuals in this discussion, so I expect there will be a variety of different experiences and opinions. That's great! If it looks to you like you have a different opinion than most others, please be sure to share it! It is important that you all be honest, and respectful of others who might think differently than you do.

I'm hoping to learn as much from you as possible, and encourage you not only tell me **what** you think, but **WHY**. The more details you share to explain your opinions, the better! This is not like Twitter or other social media where there's a limit to how much you can write. In fact, it's just the opposite! Tell me as much as you want in response to each question, especially WHY your thoughts and opinions are what they are. Please share examples or experiences that you have had that help to explain your response. For example, "Just last week, I was going to the grocery store, and…." Or "My niece told me that one of her teachers said...." Stories or examples like this really help me to understand what shapes your opinions.

I will be posting new questions each day – once in the morning, and once in the early afternoon, so it is important that you visit the website at least twice every day. It will probably take you about 30 minutes per day to answer the questions. You can spend as much time on the site as you desire.

To answer a question or respond to a post, simply click on the button in the bottom left corner that says "You have not replied. Click here," type your response in the space provided, and click on the "Submit" button. Please completely answer each question fully and be as conversational as possible, details are great! Any questions that you have not answered will have a button stating "You have not replied. Click here". This way you can easily tell what you still need to answer.

Keep an eye out for follow-up questions from me. I may ask questions of you specifically or to the group as a whole. To easily see if you have follow-ups you can

For peer review only - http://bmjopen.bmj.com/site/about/guidelines.xhtml

look at your Project Alerts box on the left of your screen and if you see a number beside "Unanswered Follow Up Questions" you'll know I have put something in specifically for you. Just click on that number and it will be brought up on the screen for you.

Here are some more tips for you to make this process easy and enjoyable:

- Please watch the “Participant Intro Video” found on the “Dashboard (Home)” page of this site. There is a lot of really great information in there to help you out along the way!
- Please upload a photo or an avatar of yourself so that I can associate a face with your name. It just makes our discussion a little more personable. You can do this under “My Profile” in the upper left-hand corner of the site.
- You can click “Highlight Unanswered Questions” on the top of the Navigation Section to see if there are any questions from me that you may have missed. To make things even easier…on the left-hand side of the screen you’ll see the number of questions or follow ups that I may have left for you! Just look for the blue box titled “Project Alerts.”
- On the left of your screen you’ll see a tab titled “Message Center”. Click on that feature to see any emails that I might have sent to you that you might have missed in your personal email inbox.
- There are some really good help articles and “How-To’s” HERE if you run into questions about how to use QualBoard (you can also click “Help Desk” on the top right of your screen to get to this site).

If you have technical difficulties of any kind while participating in this discussion, please click “Chat with QualBoard Support” at the bottom left of your screen. A technical representative will reply promptly during normal business hours (within 24 hours of your request).

One other thing -- typos don’t matter, we all make them!

For peer review only - http://bmjopen.bmj.com/site/about/guidelines.xhtml

DAY 1:

Health and Well-being Concerns

- Please take a moment to introduce yourself. Your first name only is fine. I'd like to know where you live (city, state), and who all lives at home with you, if anyone.
  - Do you have any children who live with you?
  - Do you have any elderly people who live with you, or who you take care of?

- When it comes to your health, what sorts of things are you concerned about? Please describe all of the concerns that you think about, specific to your own health, from time to time.

- What kinds of actions or precautions do you take to address these concerns, if any? Please explain for each health concern that you named.

- What about your family's health – what heath concerns, if any, do you have for others in your family? Please explain in detail.

- What kinds of actions or precautions do you take to address these concerns, if any? Please explain for each health concern that you named.

Sources of Health and Medical Information and Advice

- When it comes to your personal health, where do you turn for information and advice? Please list all of the sources you turn to.

- Who or what do you trust when it comes to health or medical advice? What makes this source/these sources trustworthy? Please explain in detail for each one.

- Who, if anyone, do you trust about health and medical issues aside from doctors or nurses? Please explain what makes you trust each of these people.

- Do you ever do your own research on health topics? If so, where do you start and what sources do you like to use? Please walk me through your process, in detail.

- What happens when you come across health-related information that contradicts other sources of health information? How do you decide what to believe or what to act upon? Please explain in detail, and share examples that may come to mind.

- Do you have a personal or family physician that you go to? If so, what are your thoughts about your doctor? Please describe your level of comfort sharing health concerns with your doctor.

  - If you do not have a regular family doctor or primary care physician, where do you go for medical advice or for medical treatment?

For peer review only - http://bmjopen.bmj.com/site/about/guidelines.xhtml

- How would you describe your level of trust with your personal physician? Why is that? Please explain your answer.

- Are there times you find your doctor's advice convincing and other times you question what he or she tells you? Please describe these situations in detail.

- Can you think of a time when you questioned your doctor's advice? Please explain that situation in detail.
    - Did you talk to your doctor about your concerns about this advice?
    - Did you follow the advice anyway, or did you do something else?

- Are there products or home remedies you like to use to stay healthy or to recover from illness? If so, please share examples of these, including when and why you use them.
    - How did you decide that this product or remedy was right for you? Please explain in detail.

Experiences and Attitudes with Respect to Vaccines in General

Welcome back! I'd like to learn about your experiences with getting vaccines, and what you think of vaccines in general. We will talk specifically about the Covid vaccine later in this discussion. For now, I'm interested in **other kinds of vaccines, not the Covid vaccine**, starting with ones you may have gotten when you were a child.

- Think back to when you were a child. Do you recall your parents taking you to get vaccinations – such as vaccinations for measles, tetanus, diptheria, etc.? What do you remember about these experiences?
    - Were these vaccines required where you grew up?

- For those of you with young children, have you taken them to get the standard vaccines for their age group?
    - What are your thoughts about these recommended vaccines for children?
    - How important do you find such vaccines to be?

- As an adult, which vaccines have you received (other than the Covid vaccine)? Please list them, and explain why you chose to get each one.

- How necessary have you found flu vaccines to be for your own health and well-being during the flu season?

- How regularly have you gotten flu shots in recent years?

- If you get the flu shot some years, but not others, what is it that makes you decide to get it some years, but not others?

For peer review only - http://bmjopen.bmj.com/site/about/guidelines.xhtml

- If anything prevented you from getting a flu shot in past years, please explain what it was?

- How much do you typically worry about getting the flu?

- Is there anything that makes you hesitant to get a flu shot? If so, what? Please explain in detail.

- Have you ever had a bad experience with a vaccine? If so, please describe what happened in detail.

- Have you read or heard anything in the past about the safety of vaccines (not including the COVID vaccine) or the need for vaccines, in general? If so, please explain in detail what the safety concerns are that you have heard or read.

    - Where did you hear or read this information?

    - How credible did you find this information about the safety of vaccines or the need for vaccines? Why is that?

    - What, if anything, did you do to try to confirm what you heard or double-check the information?

- Have you ever heard contradictory messages on the safety of vaccines? For example, some sources saying that they are safe and others saying that they are unsafe? Please explain.

- Where or from whom do you usually get your news or information on vaccines? Please be as specific as possible.

    - How much do you trust these sources of information? Please explain why you may or may not trust each one.

- What social media platforms, if any, do you use regularly?

- What impact do you think these social media platforms have had on your feelings and concerns around vaccines? Please explain and use examples, if relevant.

- Do you know of or follow any social media groups that have been talking about vaccines, or sharing information about them?

    - If so, what do you think about the information shared in these groups? How do these groups make you feel?

- When it comes to the need for vaccines and the safety of vaccines, how do you decide which side of the debate to trust? Please explain in detail.

For peer review only - http://bmjopen.bmj.com/site/about/guidelines.xhtml

- How do you decide if someone talking about vaccines is an expert in this area or not?
    - What makes someone an expert in the area of vaccines and vaccine safety? Please explain your opinion in detail.
- For those of you with a primary care physician, do you consider that doctor to be an expert on vaccines? Why or why not?
- Are there some vaccines you trust and others you don't? If so, how do you decide? Please explain in detail.

That's all of the questions for today. Thank you for sharing your thoughts and opinions so far! I look forward to learning more from you in the next session!

DAY 2:

Welcome back! At this point, I'd like to turn our conversation to the COVID vaccines that are available.

When it comes to advice and information on Covid vaccines, you may be hearing different things from many different sources – from news stations, local health officials, national health officials, church leaders, community groups, community leaders, websites, and YouTube channels, among others. We'd like to explore which of these sources of information you rely on or trust more and which you rely on or trust less.

- What kinds of things have you heard about the COVID-19 vaccines that have been developed?
- Have you heard or read anything that makes you concerned regarding the safety or the effectiveness of any of the new COVID vaccines?
    - If so, what specifically have you heard that concerns you? Please explain in detail, and give examples.
    - Where did you hear or read this?
- What about the information or source made you trust it?
- What most frightens you about getting a COVID vaccine?
- Which to you is more frightening: getting a Covid vaccine or getting the COVID-19 virus itself?

For peer review only - http://bmjopen.bmj.com/site/about/guidelines.xhtml

- How frightened are you that you might pass Covid along to people you care about?

- Does what you've heard make you think you might want to get the vaccine eventually – if your concerns are addressed? Why or why not?

- MULTIPLE CHOICE. Based on what you have seen and heard about the Covid vaccines so far, which of the following describes your attitude towards getting a Covid vaccine?

  - ❑ I will definitely get it as soon as I can.
  - ❑ I will probably get it as soon as I can.
  - ❑ I will probably get it eventually but want to wait until I know it definitely works.
  - ❑ I will probably get it eventually but want to wait until I know it is safe.
  - ❑ I will probably not get it.
  - ❑ I will definitely not get it.

- How necessary is getting vaccinated in preventing you from getting sick or dying from Covid?

- How effective do you think the vaccine would be in protecting you from Covid?

- Are there other, practical reasons that are preventing you or people you know from getting a Covid vaccine, or making it harder to do so?

- Do you have people in your family or community who feel they may not be eligible to get a Covid vaccine? If so, why do they think they wouldn't be eligible?

- Who else is involved in your decision and ability to get a Covid vaccine? Would you need to get permission from anyone else in the family before you are could get a vaccine?

- Is there disagreement within your family – some family members who want to get a Covid vaccine and others who don't? If so, how will that be resolved?

- Have you ever discussed the Covid vaccine or expressed your concerns about it with a medical professional, such as a doctor or pharmacist? If not, why not? If so, did they advise you to get a vaccine? What reasons did they give?

- Do you know people who have been vaccinated against Covid? Do you have friends who have been vaccinated? If so, how has this affected your thinking about the vaccine?

For peer review only - http://bmjopen.bmj.com/site/about/guidelines.xhtml

- If you have a church you belong to, have church leaders made any recommendations about the Covid vaccine? If so, what have they recommended?
- How important to you is the advice of church leaders on this subject?
- How do you weigh their recommendations against those of health professionals if they contradict one another?
- What about local community groups? What kinds of messages have you heard from community centers or other local organizations about the vaccine?
- How important or relevant to you are their recommendations in this regard?
- What kind of things do you hear from people in your neighborhood/community? Are many people in your community outspoken about the Covid vaccine and whether or not it is safe?
- And what about social media, what do you hear about the Covid vaccine on the social media sites you use?
- How much do you trust things people are posting on social media about the Covid vaccine?
- Can you give an example of a source you trust on social media?
- What about the Pharmaceutical companies that developed the Covid vaccines – how much do you generally trust their products?
- Have you heard anything that makes you question their development of the Covid vaccines?
- What about your local or state public health officials? What has been their advice regarding the Covid vaccines?
- Do you find their statements trustworthy or re-assuring? Why or why not?
- Do you find advice from local health officials to be more relevant or compelling than advice from national health officials? Why or why not?
- How much do you trust the recommendations or advice of public health leaders in the government, such as Dr. Anthony Fauci, Director of the National Institute of Allergy and Infectious Diseases and member of the White House Coronavirus Task Force?
- What about the Centers for Disease Control (CDC)? How familiar are you with this organization?

For peer review only - http://bmjopen.bmj.com/site/about/guidelines.xhtml

- Had you heard of it before the Covid pandemic?
- Do you trust its recommendations on how to protect yourself against Covid?
- Has your trust in the recommendations of the CDC changed over the course of the pandemic? Is so, how?
- How important is it to you to know that the FDA (the U.S. Food and Drug Administration) had authorized the new vaccine to feel it would be safe for you to get?
  Does this reassure you as to the safety of the Covid vaccines?
- Have you heard anything that makes you question the FDA's authorization of the Covid vaccines?
- Are there any reasons why you find government reassurances about health issues like Covid to be unconvincing?

DAY 3:

- How has your community been affected by Covid?
- How has Covid affected the neighborhood you live in? Has it seen a lot of cases?
- Have you heard of people around you getting seriously ill or dying from Covid?
- How has your family been affected? Do you have family members or close friends who have gotten seriously ill from Covid? How does this affect your thinking about the value of getting a vaccine?
- How worried are you about catching Covid? Does the idea of getting Covid frighten you?
- How frightening is the idea of your parents or other older family members getting it?
- Have you caught Covid? If so, how bad was it? If so, how does this make you feel about getting vaccinated?
- How do you feel that being Black/African-American affects your risk of catching Covid or of having serious reactions to Covid?
- Do you know of people here who want to get a Covid vaccine but are having trouble getting it?

For peer review only - http://bmjopen.bmj.com/site/about/guidelines.xhtml

- How does this influence your thinking about the Covid vaccine?

- Does this make you feel lucky or privileged to live in a country and a time when it is possible to be vaccinated against a potentially deadly disease?

- How much do you feel that it is a privilege to be able to get vaccinated, given that there are many countries where people desperately want it and can't get it.

- Do you have any religious objections to the vaccines? Has any clergy person discouraged you from getting vaccinated? IF SO, PROBE RE: nature of the objection or the reason for the discouragement

- How important do you feel it is to sooner or later get a Covid vaccine for your own health?

- How important do you feel it is to get a vaccine to help protect your family members or others you come in contact with who might be very vulnerable to having a serious reaction to Covid?

- How important do you feel it is for people to get a Covid vaccine to help restore normalcy??

- Do you think certain people should be required to get them? Why or why not?

- Do you think everyone should be required to get them? Why or why not?

- Who or what do you think would convince you that a Covid vaccine is safe?

- What are the best arguments you have heard for getting the vaccine?

- Even if you don't think you personally need it, are there other reasons you might want to get it? If so, please explain.

- To what extent should it be a matter of individual need or choice versus a matter of community responsibility?

- What would make you feel better or more confident of the safety of Covid vaccines?

- Whose endorsement of vaccination would you need to see?

- Would it have to be a leader in your community? Someone in your family? Someone in your church?

For peer review only - http://bmjopen.bmj.com/site/about/guidelines.xhtml

- As an African American, whose endorsement of a Covid vaccine would carry the most weight for you? Is there an individual or a group or organization that place a great deal of trust in with regards to this issue?

- Now I'm going to present some facts about the Covid vaccine, and I'd like to get your reaction acts and hear how these facts might affect your thinking.

- How does it affect your thinking about Covid vaccines that it helps protect the most vulnerable family members and community members?

- How does it affect your thinking about Covid vaccines that doctors and healthcare workers trust the vaccine and are getting them.

- How does it affect your thinking that hundreds of millions have now gotten the vaccine safely?

- How does it affect your thinking to see that getting vaccinated is helping us get our normal lives back, and ending social isolation?

- What part do you think vaccination should play in helping us open up and return to normal, and eliminating ongoing fear of Covid.

Thank you all for your participation and for sharing your thoughts. Is there anything else about the Covid vaccine that you would like to share before we close?

For peer review only - http://bmjopen.bmj.com/site/about/guidelines.xhtml

Standards for Reporting Qualitative Research (SRQR)*

http://www.equator-network.org/reporting-guidelines/srqr/

Title and abstract

	Page/line no(s).
**Title** - Concise description of the nature and topic of the study Identifying the study as qualitative or indicating the approach (e.g., ethnography, grounded theory) or data collection methods (e.g., interview, focus group) is recommended	1
**Abstract** - Summary of key elements of the study using the abstract format of the intended publication; typically includes background, purpose, methods, results, and conclusions	2

Introduction

**Problem formulation** - Description and significance of the problem/phenomenon studied; review of relevant theory and empirical work; problem statement	4-6
**Purpose or research question** - Purpose of the study and specific objectives or questions	4-6

Methods

**Qualitative approach and research paradigm** - Qualitative approach (e.g., ethnography, grounded theory, case study, phenomenology, narrative research) and guiding theory if appropriate; identifying the research paradigm (e.g., postpositivist, constructivist/ interpretivist) is also recommended; rationale**	10-11
**Researcher characteristics and reflexivity** - Researchers' characteristics that may influence the research, including personal attributes, qualifications/experience, relationship with participants, assumptions, and/or presuppositions; potential or actual interaction between researchers' characteristics and the research questions, approach, methods, results, and/or transferability	7-9
**Context** - Setting/site and salient contextual factors; rationale**	7
**Sampling strategy** - How and why research participants, documents, or events were selected; criteria for deciding when no further sampling was necessary (e.g., sampling saturation); rationale**	7-9
**Ethical issues pertaining to human subjects** - Documentation of approval by an appropriate ethics review board and participant consent, or explanation for lack thereof; other confidentiality and data security issues	7
**Data collection methods** - Types of data collected; details of data collection procedures including (as appropriate) start and stop dates of data collection and analysis, iterative process, triangulation of sources/methods, and modification of procedures in response to evolving study findings; rationale**	7-9

For peer review only - http://bmjopen.bmj.com/site/about/guidelines.xhtml

**Data collection instruments and technologies** - Description of instruments (e.g., interview guides, questionnaires) and devices (e.g., audio recorders) used for data collection; if/how the instrument(s) changed over the course of the study	7-9
**Units of study** - Number and relevant characteristics of participants, documents, or events included in the study; level of participation (could be reported in results)	7-9, Table 1
**Data processing** - Methods for processing data prior to and during analysis, including transcription, data entry, data management and security, verification of data integrity, data coding, and anonymization/de-identification of excerpts	7-9
**Data analysis** - Process by which inferences, themes, etc., were identified and developed, including the researchers involved in data analysis; usually references a specific paradigm or approach; rationale**	10-11
**Techniques to enhance trustworthiness** - Techniques to enhance trustworthiness and credibility of data analysis (e.g., member checking, audit trail, triangulation); rationale**	10-11

Results/findings

**Synthesis and interpretation** - Main findings (e.g., interpretations, inferences, and themes); might include development of a theory or model, or integration with prior research or theory	11-14
**Links to empirical data** - Evidence (e.g., quotes, field notes, text excerpts, photographs) to substantiate analytic findings	Table 3

Discussion

**Integration with prior work, implications, transferability, and contribution(s) to the field** - Short summary of main findings; explanation of how findings and conclusions connect to, support, elaborate on, or challenge conclusions of earlier scholarship; discussion of scope of application/generalizability; identification of unique contribution(s) to scholarship in a discipline or field	15-19
**Limitations** - Trustworthiness and limitations of findings	19-20

Other

**Conflicts of interest** - Potential sources of influence or perceived influence on study conduct and conclusions; how these were managed	1
**Funding** - Sources of funding and other support; role of funders in data collection, interpretation, and reporting	1

*The authors created the SRQR by searching the literature to identify guidelines, reporting standards, and critical appraisal criteria for qualitative research; reviewing the reference lists of retrieved sources; and contacting experts to gain feedback. The SRQR aims to improve the transparency of all aspects of qualitative research by providing clear standards for reporting qualitative research.

For peer review only

For peer review only - http://bmjopen.bmj.com/site/about/guidelines.xhtml

**The rationale should briefly discuss the justification for choosing that theory, approach, method, or technique rather than other options available, the assumptions and limitations implicit in those choices, and how those choices influence study conclusions and transferability. As appropriate, the rationale for several items might be discussed together.

Reference:

O'Brien BC, Harris IB, Beckman TJ, Reed DA, Cook DA. **Standards for reporting qualitative research: a synthesis of recommendations.** *Academic Medicine*, Vol. 89, No. 9 / Sept 2014 DOI: 10.1097/ACM.0000000000000388

For peer review only
